# Cytoplasmic p21 promotes stemness of colon cancer cells via activation of the NFκB pathway

**DOI:** 10.1002/1878-0261.70150

**Published:** 2025-11-03

**Authors:** Arnatchai Maiuthed, Kerstin Huebner, Katharina Erlenbach‐Wuensch, Chuanpit Hampel, Daniela Thalheim, Adriana Vial‐Roehe, Bodee Nutho, Susanne Merkel, Arndt Hartmann, Pithi Chanvorachote, Regine Schneider‐Stock

**Affiliations:** ^1^ Department of Pharmacology, Faculty of Pharmacy Mahidol University Bangkok Thailand; ^2^ Centre of Biopharmaceutical Science for Healthy Ageing, Faculty of Pharmacy Mahidol University Bangkok Thailand; ^3^ Department of Experimental Tumor Pathology Universitätsklinikum, Friedrich‐Alexander Universität Erlangen‐Nürnberg (FAU) Erlangen Germany; ^4^ Institute of Pathology Universitätsklinikum, Friedrich‐Alexander Universität Erlangen‐Nürnberg (FAU) Erlangen Germany; ^5^ Department of Pathology Federal University of Health Sciences of Porto Alegre (UFCSPA) Porto Alegre Brazil; ^6^ Department of Pharmacology, Faculty of Science Mahidol University Bangkok Thailand; ^7^ Department of Surgery Universitätsklinikum, Friedrich‐Alexander Universität Erlangen‐Nürnberg (FAU) Erlangen Germany; ^8^ Center of Excellence in Cancer Cell and Molecular Biology, Faculty of Pharmaceutical Sciences Chulalongkorn University Bangkok Thailand; ^9^ Department of Pharmacology and Physiology, Faculty of Pharmaceutical Sciences Chulalongkorn University Bangkok Thailand; ^10^ Adjunct Professor, Faculty of Pharmacy Mahidol University Bangkok Thailand

**Keywords:** BCL‐xL, CAM model, CD133, IκB, NFκB, stem cells

## Abstract

Cancer stem cells (CSCs) drive tumor initiation, metastasis, and therapy resistance. The role of cytoplasmic cyclin‐dependent kinase inhibitor 1A (CDKN1A, p21) in CSC biology remains unclear. Since cytoplasmic p21 correlated with advanced stage and metastasis in colorectal cancer (CRC) patients, we investigated its causal role in CSC features *in vitro* and *in vivo*. Cytoplasmic p21 increased spheroid formation and CD133 expression in a mechanism partly dependent on AKT activation. Phosphomimetic p21 (p21^T145D^) enhanced spheroid growth, CD133, and stemness factors (Oct3/4, Nanog, Sox2), whereas nuclear p21 (p21^T145A^) reduced them. Immunoprecipitation, proximity ligation assays, and *in silico* modeling demonstrated that cytoplasmic p21 interacts with the NFκB–IκB complex, promoting NFκB release and activation. Consequently, NFκB targets BCL‐xL and COX2 were upregulated in p21^T145D^‐ and AKT^T308D,S473D^ CRC cells *in vitro* and in a chorioallantoic membrane (CAM) model, supporting their role as downstream effectors of cytoplasmic p21. Our findings uncover a new function of cytoplasmic p21 in regulating CSC properties through NFκB modulation. Screening p21 subcellular localization may stratify CRC patients with high metastatic risk providing a basis for CSC‐targeted therapeutic strategies.

## Graphical abstract

Cytoplasmic p21 promotes colorectal cancer stem cell (CSC) features by destabilizing the NFκB–IκB complex, activating NFκB signaling, and upregulating BCL‐xL and COX2. In contrast to nuclear p21, cytoplasmic p21 enhances spheroid formation and stemness transcription factor CD133. Our findings identify cytoplasmic p21 as a driver of CSC traits and a potential predictor of metastasis risk.

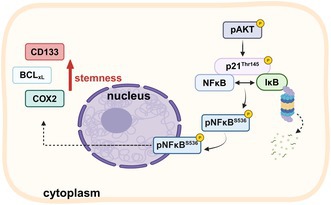

**Abbreviations**
2D: 2 dimension
3D: 3 dimension
5‐FU: 5‐Fluorouracil
AKT: protein kinase B
AKT^T308D,S473D^
: phosphorylated mimic form of AKT at T308 and S473
APF: antiproliferative factor
ATCC: American Type Culture Collection
BCL‐xl: B‐cell lymphoma‐extra large
BRAF: B‐Raf proto‐oncogene, serine/threonine kinase
BSA: bovine serum albumin
CAM: chorioallantoic membrane
CD133: Prominin‐1
CD44: CD44 antigen
CDK: cyclin‐dependent kinase
CDKN1A, p21: cyclin‐dependent kinase inhibitor 1A
CHX: cycloheximide
CIP/Kip: CDK interacting protein/kinase inhibitory protein
CMS4: consensus molecular subtype 4
CO_2_
: carbon dioxide
Co‐IP: coimmunoprecipitation
COX2: cyclooxygenase‐2
CRC: colorectal cancer
CSC: cancer stem cell
DAPI: 4',6‐diamidino‐2‐phenylindole
DMEM: Dulbecco's modified Eagle medium
E2F: E2F transcription factor
E‐cadherin: epithelial cadherin
EDD8: embryonic development day 8
ERK2: extracellular signal‐regulated kinase 2
FFPE: formalin‐fixed paraffin‐embedded
FOXP3: forkhead box P3
GAPDH: glyceraldehyde‐3‐phosphate dehydrogenase
GATA4: GATA‐binding protein 4
GSC: homeobox protein goosecoid
HCG: human chorionic gonadotropin
HIF‐1α: hypoxia‐inducible factor
HNF‐3beta/FoxA2: hepatocyte nuclear factor 3‐beta/forkhead box protein A2
HRP: horseradish peroxidase
IF: immunofluorescence
IgG: immunoglobulin G
IL22RA1: interleukin 22 receptor subunit alpha 1
IκB: inhibitor of kappa B
MCLD: multicellular limiting dilution
Met: patients with liver metastasis
Nanog: homeobox protein NANOG
NCK2: NCK adaptor protein 2
NFκB: nuclear factor kappa‐light‐chain‐enhancer of activated B cells
NO: nitric oxide
Oct3/4: octamer‐binding transcription factor 3/4
Otx2: orthodenticle homeobox 2
P21^T145A^
: unphosphorylated mimic form of p21 at Threonine 145
P21^T145D^
: phosphorylated mimic form of p21 at Threonine 145
P53: tumor protein p53
p‐AKT^S473^
: phosphorylated form of AKT at serine 473
PBS: phosphate‐buffered saline
p‐Chk2: phosphorylated checkpoint kinase 2
PDB: Protein Data Bank
PDX1: pancreatic and duodenal homeobox 1
PDX‐1/IPF1: pancreas/duodenum homeobox protein 1
pH: potential of hydrogen
PI3K: phosphatidylinositol 3‐kinase
PLA: proximity ligation assay
p‐NFκB^S536^
: phosphorylated form of NFκB serine 536
p‐p21^T145^
: phosphorylated form of p21 at threonine 145
PRPF6: pre‐mRNA‐processing factor 6
PT‐M0: primary tumors without metastasis
PT‐M1: primary tumors with synchronous metastasis
RAS: rat sarcoma
Rb: retinoblastoma protein
RCSB: research collaboratory for structure bioinformatics
RPMI 1640: Roswell Park Memorial Institute
SD: standard deviation
SDHD: membrane‐anchoring subunit of succinate dehydrogenase
SDS/PAGE: sodium dodecyl sulfate polyacrylamide gel electrophoresis
Snail: snail family transcriptional repressor 1
SNAP: S‐nitroso‐*N*‐acetylpenicillamine
Sox17: SRY‐box transcription factor 17
Sox2: SRY‐box transcription factor 2
TBST: Tris‐buffered saline with Tween 20
TMAs: tissue microarrays
TP63/TP73L: tumor protein p63
TRS‐buffer: target‐retrieval solution buffer
ULA: ultra‐low attached
VEGF R2/KDR/Flk‐1: vascular endothelial growth factor receptor 2/kinase insert domain receptor/fetal liver kinase 1
YAP: Yes‐associated protein

## 1 Introduction

Cancer stem cells (CSCs), also referred to as ‘cancer‐initiating cells’, constitute a small but distinct subpopulation of cancer cells that share key characteristics with normal stem cells, including self‐renewal, multidirectional differentiation potential, and symmetric cell division [1, 2]. CSCs have been identified in various cancer types, including colorectal cancer (CRC), and are considered major drivers of cancer progression, resistance to chemo‐ and radiotherapy, metastasis, and disease relapse [3].

Research has shown that CSCs are regulated by both oncoproteins and tumor suppressor proteins, such as NFκB [4], cyclooxygenase‐2 (COX2) [5], B‐cell lymphoma‐extra‐large (BCL‐xL) [6], and p21 [7]. Notably, recent studies highlight that the activation of NFκB in cancer cells promotes CSC‐like properties across various cancer types, including CRC [4]. This phenotype is driven by NFκB directly enhancing the expression of CSC‐associated proteins as well as its downstream targets [4], such as COX2 [5] and BCL‐xL [6, 8].

BCL‐xL has been observed to be highly expressed in CRC specimens with aggressive phenotypes, supporting the expression of CSC‐associated proteins such as SDHD, CD44, PDX1, NCK2, IL22RA1, and PRPF6 [9, 10]. Similarly, COX2 expression is markedly elevated in CRC tissues compared to adjacent noncancerous tissue [11], with its levels correlating with cancer progression [12]. COX2 appears to expand the CSC population in conjunction with transcription factors such as YAP [13].

The regulation of NFκB is primarily mediated by its interaction with IκB, which forms a complex with NFκB and inhibits its activity. Disruption of this IκB‐NFκB complex leads to IκB ubiquitination and degradation, allowing NFκB to translocate to the nucleus and activate gene expression. This destabilization can be triggered by interactions with various proteins, both enzymatic and nonenzymatic [14, 15].

P21, a member of the CIP/Kip family of cyclin‐dependent kinase (CDK) inhibitors, plays a critical role in cellular processes such as cell cycle regulation, senescence, and apoptosis modulation [16]. Despite being nonenzymatic, p21 exerts its diverse effects through its scaffolding function, interacting with and inhibiting CDKs to prevent Rb phosphorylation and the release of E2F transcription factors [17, 18]. Furthermore, p21 contributes to cellular senescence via protein–protein interactions in both p53‐dependent and independent pathways [19]. Importantly, the regulation of apoptosis by p21 is mediated not only by transcriptional mechanisms but also by its interactions with other proteins and its subcellular localization.

The subcellular localization of p21 plays a significant role in normal cellular physiology and disease states, particularly in cancer. Indeed, contrasting functions of p21 in cancer progression are closely tied to its localization: nuclear p21 inhibits cell cycle progression and promotes apoptosis [20, 21], whereas cytoplasmic p21 is associated with oncogenic activities such as chemoresistance [22], proliferation, and metastasis [23]. These aggressive cancer phenotypes driven by cytoplasmic p21 have been linked to CSC properties, although the precise mechanisms by which cytoplasmic p21 regulates CSCs remain unclear.

In our study, we identified cytoplasmic p21 as a driver of CSC characteristics in CRC by mediating the destabilization of the NFκB‐IκB complex through protein–protein interactions, leading to IκB degradation and subsequent activation of the NFκB pathway.

## 2 Materials and methods

### 2.1 Cell culture

The mycoplasma‐free human colorectal cancer cell lines HCT116 (RRID:CVCL_0291), HT29 (RRID:CVCL_0320), and SW837 (RRID:CVCL_1729) were purchased from ATCC (Manassas, VA, USA). The cells were cultured in RPMI 1640 (Gibco Life Technologies, Carlsbad, CA, USA) supplemented with 10% fetal bovine serum (PAN Biotech, Aidenbach, Germany), penicillin (100 U·mL^−1^) and streptomycin (100 μg·mL^−1^) (PAN Biotech, Aidenbach, Germany). HCT116 p21^−/−^ cells (RRID:CVCL_HD73) were cultured in DMEM (Gibco Life Technologies) supplemented with 10% fetal bovine serum (PAN Biotech), penicillin (100 U·mL^−1^), streptomycin (100 μg·mL^−1^) (PAN Biotech), 1% l‐glutamine (PAN Biotech), and 1% essential amino acids (Gibco Life Technologies). All cell lines were maintained in 5% CO_2_ at 37 °C in a cell culture incubator. The cell lines were routinely used or subcultured at 70% confluence. All experiments were performed within 15 passages of the cells. All cell lines were genotyped using Multiplex Cell Authentication by Multiplexion (Heidelberg, Germany). All cell lines were regularly tested negative for mycoplasma contamination.

### 2.2 Multicellular limiting dilution spheroid assay (MCLD)

About 2000 cells and six sequential twofold dilutions of HT29, HCT116, or SW837 were seeded into a well of a 96‐well ULA plate (Corning Inc., Corning, NY, USA). The culture medium used was RPMI 1640 (Gibco Life Technologies) supplemented with 100 U·mL^−1^ penicillin and 100 μg·mL^−1^ streptomycin (PAN Biotech) and without fetal bovine serum (PAN Biotech). Spheroids were cultured at 37 °C in an atmosphere of 5% CO_2_ for 5 or 10 days. After the incubation period (5 or 10 days), spheroid images were captured under a microscope for measuring number and size using the imagej program (National Institutes of Health, Bethesda, MD, USA). Thereafter, spheroids were collected and fixed with 4% formaldehyde for 30 min and stained with relevant antibodies.

### 2.3 Patients

Formalin‐fixed paraffin‐embedded (FFPE) colon tumor tissue samples were available at the Universitätsklinikum Erlangen, Germany. The requirement for formal ethics approval was exempted by the ethics committee of the Universitätsklinikum of the Friedrich‐Alexander Universität Erlangen‐Nürnberg (23‐323‐Br). A written informed consent of patients was waived by the ethics committee since all clinical data were used completely anonymously in this retrospective study. All procedures were performed in accordance with the Declaration of Helsinki. Clinical data on age, sex, diagnosis, tumor histology, and tumor stage can be found in the Table S1.

### 2.4 Reagents

To study AKT dependency, HCT116 cells were treated with the two PI3K inhibitors Wortmannin (0.1 and 1 μm; Cell Signaling Technology, Danvers, MA, USA; #9951) and LY294002 (5 and 50 μm; Cell Signaling Technology; #99001) for 72 h. For NO‐mediated effects on CSC capacity, HCT116 and HCT116 p21^−/−^ cells were treated with the nitric oxide donor SNAP (50 and 100 μm; Abcam, Cambridge, UK; ab120014) for 5 days. Further, HCT116 cells were treated with the protein synthesis inhibitor cycloheximide (CHX; Cell Signaling Technology; #2112) and with the proteasome inhibitor MG132 (Sigma‐Aldrich, St. Louis, MO, USA; M7449) for the indicated durations (30 min, 1 h, 3 h, and 6 h). Cells were then further processed for either western blot, immunofluorescence staining, or subcellular fractionation.

### 2.5 Subcellular fractionation

Subcellular fractionation into cytoplasmic and nuclear protein extracts was performed for cells grown in 2D and in 3D (as spheroids) using the Subcellular Protein Fractionation Kit for Cultured Cells (Thermo Fisher Scientific, Waltham, MA, USA; 78840) according to the manufacturer's instructions. For 3D spheroids, spheroids were initially collected and incubated in TrypLE Express Enzyme (Thermo Fisher Scientific; 12604013) at 37 °C for 30 min to generate a single cell suspension. All incubation times of the protocol were doubled for spheroid‐based fractionation. Lamin A/C and GAPDH were used as loading controls for nuclear and cytoplasmic fractions, respectively.

### 2.6 Western blot analysis

Cells pellets were collected and lysed, and western blotting was performed as previously described [22]. Briefly, after collecting the cells, cell lysates were prepared by adding lysis buffer containing a protease inhibitor cocktail (Merck, Darmstadt, Germany) to cell pellets for 90 min on ice. After SDS/PAGE, the proteins were transferred onto nitrocellulose membranes (GE Healthcare Chalfont, St. Giles, UK). After blocking for 1 h in 5% nonfat dry milk (Carl Roth, Karlsruhe, Germany) in TBST (25 mm Tris/HCl pH 7.5, 125 mm NaCl; Carl Roth, Karlsruhe, Germany) and 0.05% Tween 20 (SERVA Electrophoresis GmbH, Heidelberg, Germany), membranes were incubated with a primary antibody at 4 °C overnight. Membranes were washed three times with TBST for 5 min and incubated with horseradish peroxidase‐labeled isotype‐specific secondary antibodies (anti‐mouse or anti‐rabbit IgG peroxidase conjugated; Pierce, Rockford, IL, USA) for 2 h at room temperature. The immune complexes were detected by enhancement with a chemiluminescent substrate (Merck). The level of immunoreactivity was measured as peak intensity using either the CoolSNAP HQ2 CCD camera (Photometrics, Tucson, AZ, USA) in combination with a DeVision DBOX and the Gel‐Pro^®^ Analyzer Version 6.0 software (Media Cybernetics, Cambridge, UK) or the Invitrogen™ iBright™ FL1500 Imaging System (Thermo Fisher Scientific). Antibodies used in the present study were as follows: AKT (#9272, rabbit, 1 : 10 000 dilution; Cell Signaling Technology), p‐AKT (S473) (#4060, rabbit, 1 : 1000 dilution; Cell Signaling Technology), BCL‐xL (ab32370, rabbit, 1 : 1000 dilution; Abcam, Cambridge, UK), CD133 (W6B3C1, mouse, 1 : 250, Miltenyi Biotec, Bergisch‐Gladbach, Germany), COX2 (#D5H5 (XP), rabbit, 1 : 1000 dilution; Cell Signaling Technology), Histone H3 (#4499 (XP), rabbit, 1 : 2000 dilution; Cell Signaling Technology), IκB (#4812, rabbit, 1 : 1000 dilution; Cell Signaling Technology), NFκB (#D14E12 (XP), rabbit, 1 : 2000 dilution; Cell Signaling Technology), p‐NFκB (S536) (#3036, mouse, 1 : 1000, Cell Signaling Technology), p21 Waf1/Cip1 (#12D1, mouse, 1 : 2000 dilution; Cell Signaling Technology, Danvers, MA, USA), Lamin A/C (ab108595, rabbit, 1 : 10 000 dilution, Abcam), FLAG (#8146, 1 : 2000 dilution, Cell Signaling Technology). HRP‐conjugated anti‐GAPDH (1 : 50 000–1 : 200 000, BSA, Abnova, Taipei, Taiwan) was used to control equal loading and protein quality.

### 2.7 Immunofluorescence

After the specific experimental procedure, the colorectal cancer cell lines were fixed with 4% formaldehyde for 30 min and then permeabilized with 0.1% Triton X for 10 min. Thereafter, the cells were incubated with 3% bovine serum albumin (BSA; Sigma‐Aldrich, St. Louis, MO, USA) for 30 min to prevent nonspecific binding. The cells were washed and incubated with specific primary antibodies (see figure legend for details); p21 Waf1/Cip1 (#12D1, rabbit, 1 : 500 dilution; Cell Signaling Technology), CD133 (W6B3C1, mouse, 1 : 250, Miltenyi Biotec, Bergisch‐Gladbach, Germany), phosphorylated NFκB (Ser536) (93H1, rabbit, 1 : 1000, Cell Signaling Technology) at 4 °C for 24 h. The primary antibody was removed, and the cells were washed with phosphate‐buffered saline (PBS) and subsequently incubated with a specific secondary antibody conjugated with a suitable fluorescent dye (described in the figure) (Invitrogen, Carlsbad, CA, USA) for 1 h at room temperature. F‐actin was stained with Alexa Fluor 670‐conjugated Phalloidin (Invitrogen, Carlsbad, CA, USA). Nuclei were counterstained with DAPI (Sigma‐Aldrich). The samples were washed with PBS and then visualized and imaged by fluorescence microscopy.

### 2.8 Plasmids and transfection

Flag p21 T145D was a gift from Mien‐Chie Hung (Addgene plasmid #16242; http://n2t.net/addgene:16242; RRID:Addgene_16242) [24], Flag p21 T145A was a gift from Mien‐Chie Hung (Addgene plasmid #16241; http://n2t.net/addgene:16241; RRID:Addgene_16241) [24], HA PKB T308D S473D pcDNA3 was a gift from Jim Woodgett (Addgene plasmid #14751; http://n2t.net/addgene:14751; RRID:Addgene_14751) [25], and the mock vector FNpCDNA3 was a gift from Robert Oshima (Addgene plasmid #45346; http://n2t.net/addgene:45346; RRID:Addgene_45346).

Transient transfection was performed for 24–48 h by using Lipofectamine 3000 Transfection Reagent according to the manufacturer's instructions.

### 2.9 Coimmunoprecipitation

HCT116 cells were seeded in 60‐mm cell culture dishes at a concentration of 5 × 10^5^ cells per dish for 24 h. After that, the cell lysates of the remaining cells were collected, prepared, and subjected to co‐IP as previously described [22]. The levels of proteins involved in IP‐protein interactions were determined by western blotting.

### 2.10 Chorioallantoic membrane assay (CAM assay)

The CAM assay, an alternative xenograft model, was performed as previously described [22]. Briefly, fertilized chicken eggs were incubated at 37 °C with 70% humidity until EDD8. Then, a small hole was pricked into the eggshell of the more rounded pole, where the air sac resides. After dropping of the egg content, a small window (Ø 1–1.5 cm) was carefully cut at the more rounded pole for inoculation of the tumor cells. After another day of incubation (Day 9 of embryonic development), 1 × 10^6^ SW837‐transfected (p21^145D^, AKT^T308D,S473D^) cells or control SW837 cells in a 1 : 1 mixture of Matrigel and medium were implanted onto the CAM, and the eggs were incubated for 5 days. Finally, the microtumors were harvested, fixed in 4% phosphate‐buffered formalin, and embedded in paraffin for histological and immunohistochemical analysis.

### 2.11 Proximity ligation assay *in situ* (PLA)

HCT116 cells were grown on 8‐well chamber slides to 50–60% confluence. The cells were then fixed in 4% paraformaldehyde in phosphate‐buffered saline (PBS) for 30 min, permeabilized in 0.1% Triton X‐100 for 20 min, and blocked with Duolink II blocking solution for 1 h. After blocking, the cells were incubated with two primary antibodies, anti‐p21 and anti‐IκB or anti‐p21 and anti‐NFκB, at 4 °C overnight, following the manufacturer's instructions for proximity ligation. Fluorescence images were acquired using a fluorescence microscope, ligation signals were visualized by red dots, and cell nuclei were visualized by Hoechst 33342 (blue).

### 2.12 Immunohistochemistry (IHC)

CAM tumors were paraffin‐embedded, subjected to serial sectioning (2 μm) and mounted on precoated slides for immunohistochemical analysis. All formalin‐fixed paraffin‐embedded (FFPE) whole CAM tissue sections and tissue microarrays (TMAs) were deparaffinized with xylene and rehydrated with graded ethanol. Antigen retrieval was performed by 1 min of steam cooking in target‐retrieval solution buffer (TRS‐Buffer) at pH 9. Slides were incubated at 4 °C overnight with primary monoclonal antibodies against p21 Waf1/Cip1 (#12D1, mouse, 1 : 2000 dilution; Cell Signaling Technology), BCL‐xL (rabbit, 1 : 2000 dilution, Cell Signaling Technology), or COX2 (rabbit, 1 : 2000 dilution, Cell Signaling Technology). Antibody binding was visualized using an Anti‐Rabbit/ABC Kit (Vector) or Polymer Kit (AP, Zytomed Systems, Berlin, Germany).

### 2.13 Human pluripotent stem cell Array

The human pluripotent stem cell array (ARY010, R&D Systems/Bio‐Techne, MN, USA) was performed according to the manufacturer's instructions. For this, SW837 cells were transfected with either mock, AKT^T308D,S473D^ or p21^T145D^ plasmids (see Section 2.8) for 48 h using Lipofectamine 3000 Transfection Reagent according to the manufacturer's instructions. Cell lysates were prepared with the buffers provided in the kit and incubated overnight at 4 °C on the array membranes. After overnight incubation and subsequent washing steps, array membranes were probed with the Detection Antibody Cocktail for 2 h at room temperature. Membranes were washed again and incubated with Streptavidin‐HRP solution for 30 min at room temperature. Upon washing the array membranes, signals were detected using the provided Chemi Reagent Mix and the GeneGnome detection system (Syngene, Bangalore, India). Obtained signals were quantified by densitometry using imagej.

### 2.14 Computational study

The X‐ray crystal structure of the IκB/NFκB p50/p65 heterodimer complex was obtained from the RCSB Protein Data Bank (PDB ID: 1IKN) [26]. The full‐length structure of p21 (164 amino acids) was retrieved from the alphafold Protein Structure Database (https://alphafold.ebi.ac.uk/) [27] using the UniProt accession code P38936. Phosphorylation at threonine 145 (p‐p21T145) and its phosphomimetic mutant (p21T145D) were modeled using the Macromolecules tools in Discovery Studio Visualizer (BIOVIA, San Diego, CA, USA). Protein–protein docking was performed using cluspro 2.0 [28], with default parameters and a balanced coefficient. The structures of p‐p21T145 and p21T145D were docked to the IκB/NFκB p50/p65 heterodimer. For further analysis, the centroid of the top‐ranked cluster from each docking run was selected. All docked complexes were subjected to energy minimization using 5000 iterations of steepest descent and conjugate gradient algorithms in AMBER24, applying the AMBER ff19SB force field [29]. Total binding energy was calculated using the Molecular Mechanics/Poisson‐Boltzmann Surface Area (MM/PBSA) method [30] to evaluate the binding affinity of the NFκB p50/p65 heterodimer with IκB in the absence and presence of p‐p21T145 and p21T145D. All docked complexes were visualized using ucsf chimerax [31].

### 2.15 Statistics

All treatment data were normalized to the corresponding nontreated or transfection controls. Statistical analyses were performed with graphpad prism v. 9.5.1 (GraphPad, San Diego, CA, USA) and IBM^®^ SPSS^®^ Statistics software v. 24.0 (IBM, Armonk, NY, USA). Statistical testing, *n* numbers and *P* values for statistical significance are outlined in the figure legends with statistical significance being defined as *P* < 0.05.

## 3 Results

### 3.1 Cytoplasmic p21 correlates with tumor progression in colorectal cancer patient specimens

**Fig. 1:**
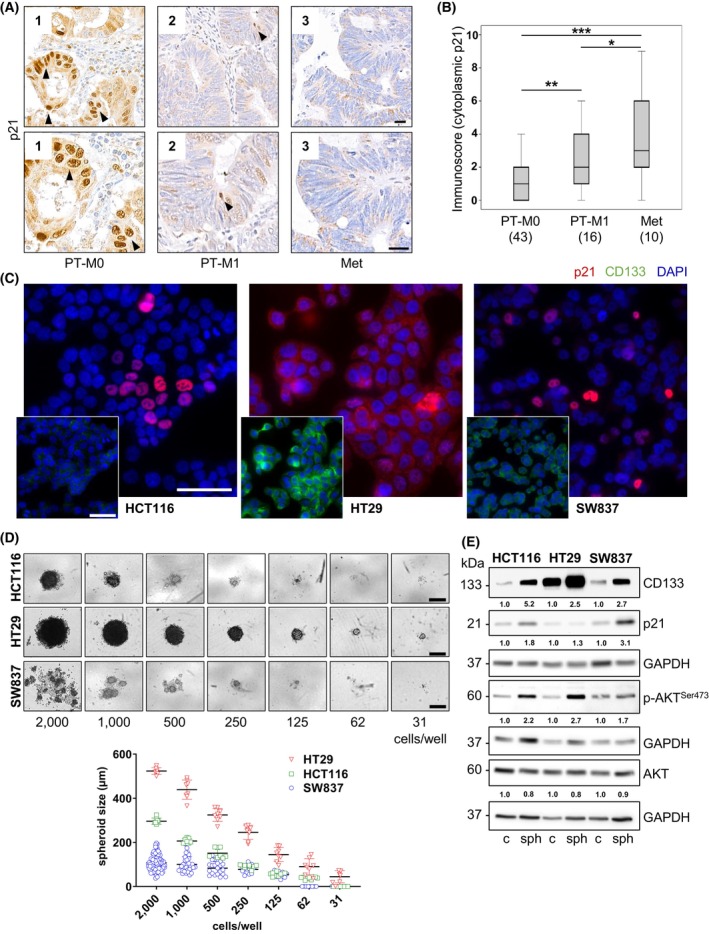
Correlations between cytoplasmic p21 and cancer stem cell characteristics. (A) Immunohistochemistry of p21 in colorectal cancer specimens revealed that the cytoplasmic localization of p21 correlated with cancer stage. PT‐M0 (*n* = 43) indicates primary tumor without metastasis, PT‐M1 (*n* = 16) indicates primary tumor with synchronous metastasis, and Met indicates liver metastasis (*n* = 10). The lower panels are the enlarged versions of the upper panels. Scale bars are 20 μm (B) The immunoscore of p21 localized in the cytoplasm of colorectal cancer specimens was evaluated. The data are presented as boxplots showing median and interquartile ranges (PT‐M0, *n* = 43; PT‐M1, *n* = 16; and Met, *n* = 10; *P < 0.05, **P < 0.01, ***P < 0.001, unpaired *t*‐test). (C) The expression of p21 and CD133 in HCT116, HT29, and SW837 cells was determined by immunofluorescence staining with a rabbit anti‐p21 antibody and mouse anti‐CD133 antibody followed by an Alexa Fluor 555‐conjugated anti‐rabbit antibody to visualize p21 (red) and Alexa Fluor 488‐conjugated anti‐mouse antibody to visualize CD133 (green). The contrast for p21 signals was adjusted manually. The cell nuclei were visualized by staining with DAPI (blue). The representative scale bars are 50 μm. Representative images of two independent experiments are shown. (D) The cancer stem cell capacity of HCT116, HT29, and SW837 cells was determined by a multicellular limiting dilution (MCLD) spheroid assay for 10 days in serum‐free medium. The representative scale bars are 250 μm. The diameter and number of spheroids were determined by imagej. Representative images of two independent experiments are shown. Statistical testing using a two‐way ANOVA revealed that HT29 spheroids were significantly larger than HCT116 and SW837 spheroids for all conditions (*P* values were ≤ 0.05). (E) The expression of CD133, p21, AKT, and p‐AKT^S473^ in 2D cultures and 3D cultures of HCT116, HT29, and SW837 cells. Cancer spheroids were generated from these colorectal cancer cell lines by 3D culture with a MCLD spheroid assay for 10 days. The spheroids (sph) and the cells in the 2D culture system (c) of these cell lines were collected, and the expression of proteins of interest was analyzed by western blot analysis. The blots were reprobed with GAPDH (1 : 50 000) to confirm equal loading of the samples. Representative blots of two independent experiments are shown.

To determine whether cytoplasmic p21 expression is correlated with tumor progression, we performed immunohistochemical staining for p21 in the colorectal cancer tissues of 69 patients [43 patients with primary tumors without metastasis (PT‐M0), 16 patients with primary tumors with synchronous metastasis (PT‐M1), and 10 patients with liver metastases (Met)]. Information of the patient cohort is given in Table S1. The cytoplasmic localization of p21 increased with disease progression, and nuclear localization of p21 was rarely observed in the metastatic patients' tissues. The PT‐M1 cytoplasmic p21 levels were significantly greater than those of the PT‐M0 group, and the highest levels were detected in the liver metastases (Fig. 1A,B).

### 3.2 Correlation of cytoplasmic p21 and cancer stem cell characteristics in colorectal cancer cell lines

Next, we investigated whether cancer stem cell characteristics in colorectal cancer cell lines correlated with cytoplasmic p21. Only p21 phosphorylated by the serine/threonine kinase AKT at residue Thr145 has been shown to accumulate in the cytoplasm [32]. Therefore, we first investigated the correlation between the cellular localization of p21 and the expression of the CSC marker CD133 (Fig. 1C), and second, we studied the spheroid formation ability of three colorectal cancer cell lines, HCT116, HT29, and SW837 (Fig. 1D). Western blot analysis revealed that the CSC marker CD133 exhibited the same trend as phosphorylated AKT (p‐AKT^s473^) (Fig. 1E), which is phosphorylated at a crucial site for AKT activation [33]. The p‐p21^T145^ protein could not be detected by western blot analysis due to the large number of unspecific bands [22]. Thus, we investigated the subcellular localization of the p21 protein in these cell lines by immunofluorescence (IF). Nuclear p21 levels as detected by IF (Fig. 1C) were inversely correlated with CD133 expression in western blot analysis (Fig. 1E), with HT29 cells showing the highest CD133 expression (Fig. 1C,E) and lowest p21 signals in the nucleus but highest p21 signals in the cytoplasm (Fig. 1C). In line with these findings, HT29 cells exhibiting the highest levels of CD133 and cytoplasmic p21 demonstrated the greatest spheroid formation potential in a multicellular limiting dilution (MCLD) assay (Fig. 1D). The levels of CD133, p21, and p‐AKT^S473^ were further elevated in the 3D tumor spheroids (Fig. 1E), indicating an enhanced manifestation of CSC characteristics. The subcellular fractionation of nucleus versus cytoplasm supported our data (Fig. S1). The upregulation of cytoplasmic p21 was more pronounced in HCT116 cells, suggesting a saturation effect in already stemness‐enriched HT29 cells. Therefore, the following mechanistic experimental approaches were conducted with only HCT116 and SW837 cells to more clearly identify changes in stemness capacity. In summary, our findings suggest that the subcellular localization of p21 might affect CSC characteristics both in human CRC tissue and *in vitro*.

### 3.3 AKT promotes the CSC phenotype and cytoplasmic p21 accumulation in colorectal cancer cell lines

**Fig. 2:**
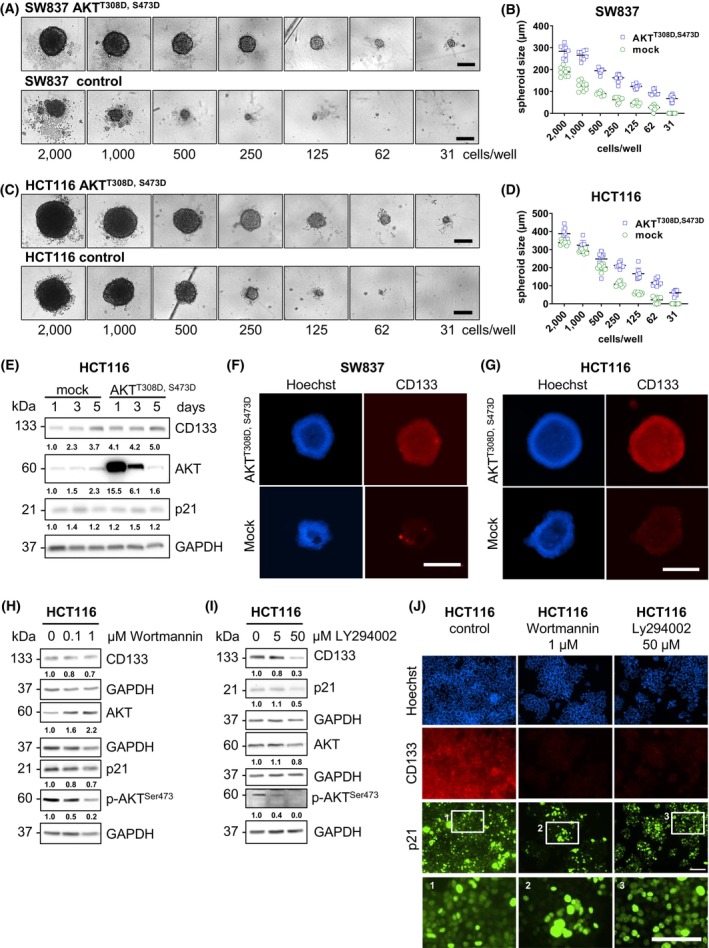
AKT activates cancer stem cell characteristics in colorectal cancer cell lines. (A, B) SW837 and (C, D) HCT116 cells were transfected with hyperphosphorylated AKT^T308D,S473D^ and then subjected to a MCLD spheroid assay in serum‐free medium for 10 days, after which the spheroid diameter was determined. The representative scale bars are 250 μm. Representative images and data of two independent experiments are shown. Statistical testing using a two‐way ANOVA revealed that AKT^T308D,S473D^ spheroids were significantly larger than mock‐transfected spheroids for both cell lines and for all conditions (*P* values were ≤ 0.05). (E) After HCT116 cells were transfected with hyperphosphorylated AKT^T308D,S473D^ for 1, 3, or 5 days, the cells were collected, and the expression levels of CD133, AKT, p‐p21^T145^, and p21 were evaluated by western blot analysis. The blots were reprobed with GAPDH (1 : 50 000) to confirm equal loading of the samples. Representative blots of two independent experiments are shown. (F) CD133 expression in SW837 and (G) HCT116 spheroids from hyperphosphorylated AKT^T308D,S473D^‐transfected cells and mock control cells were evaluated by an immunofluorescence staining assay. After 10 days of spheroid formation by limiting dilution with a MCLD spheroid assay, the cells were fixed with 4% formaldehyde and stained with a mouse anti‐CD133 antibody followed by an anti‐mouse Alexa Fluor 555‐conjugated antibody to visualize CD133 (red) and stained with Hoechst 33342 to visualize the nuclei (blue). The representative scale bars are 500 μm. Representative images of two independent experiments are shown. (H, I) HCT116 cells were treated with (H) Wortmannin (0.1 and 1 μm) or (I) LY294002 (5 and 50 μm) for 72 h. The cells were collected, and the expression levels of CD133, AKT, p‐AKT^S473^, and p21 were examined by western blotting. The blots were reprobed with GAPDH (1 : 50 000) to confirm equal loading of the samples. Representative blots of two independent experiments are shown. (J) After HCT116 cells were treated with 1 μm Wortmannin or 50 μm LY294002 for 72 h, the cells were fixed with 4% formaldehyde and stained with rabbit anti‐p21 antibody and mouse anti‐CD133 antibody followed by anti‐rabbit Alexa Fluor 488‐conjugated antibody and anti‐mouse Alexa Fluor 555‐conjugated antibody to visualize p21 as green and CD133 as red. The cell nuclei were visualized by staining with Hoechst 33342 (blue). The white boxes indicate the enlarged areas given below. The representative scale bars are 100 μm. Representative images of two independent experiments are shown.

Recently, AKT activity was shown to promote the CSC phenotype in various cancer types [34], and the shuttling of p21 from the nucleus to the cytoplasm [24]. To mechanistically examine the link between AKT activation and the regulation of CSC characteristics in our CRC cell lines, we transfected HCT116 and SW837 cells with hyperphosphorylated AKT^T308D,S473D^ (an active form of AKT) [25]. Despite seeding constant cell numbers for all conditions, we observed that the AKT^T308D,S473D^‐transfected cells formed larger tumor spheroids on day 10 than the corresponding controls (Fig. 2A–D), whereas on day 5 of the MCLD assay, the spheroid sizes of the transfected and control groups only showed minor to no differences (Fig. S2A–D). Correspondingly, the AKT^T308D,S473D^‐transfected HCT116 cells exhibited increased CD133 levels over time, as shown by western blot analysis (Fig. 2E). Moreover, higher CD133 levels were detected in spheroids of the AKT^T308D,S473D^‐transfected HCT116 and SW837 cells using immunofluorescence staining (Fig. 2F,G). Next, we treated HCT116 cells with two phosphoinositide 3‐kinases (PI3K) inhibitors, Wortmannin (0.1 and 1 μm) and LY294002 (5 and 50 μm), for 72 h. Western blot analysis revealed a strong decrease in p‐AKT^S473^, CD133, and p21 levels in a dose‐dependent manner (Fig. 2H,I). Reduced p‐AKT^S473^ and CD133 expression was accompanied by an increase in nuclear p21 signals, as shown by immunofluorescence (Fig. 2J) and subcellular fractionation (Fig. S3). These findings suggested that AKT triggered cytoplasmic p21 localization, which was associated with increased CSC potential.

### 3.4 Nitric oxide‐induced CSC characteristics are independent of p21 in CRC cell lines

As previously proven, the short‐lived free radical nitric oxide (NO) promotes pluripotency and stem cell differentiation in various cancer cell lines [35, 36]. To determine the role of NO in increasing the CSC capacity of HCT116 cells, we treated the cells with 50 and 100 μm SNAP, a NO donor. Next, the ability of the cells to form spheroids in a MDLC assay as a functional test for self‐renewal capacity was analyzed after 3 and 5 days. As expected, treatment with 50 μm SNAP for 5 days increased spheroid formation compared to untreated controls (Fig. S4A,B). Western blotting and IF revealed that SNAP promoted the expression of CD133 in a time‐ and dose‐dependent manner (Fig. S4C,D). Interestingly, this increase in CD133 levels was not accompanied by higher cytoplasmic p21 signals in IF (Fig. S4D). Indeed, CD133 expression was completely lost in HCT116 p21^−/−^ cells (Figs S4E,F and S5A,B) and was also not inducible by AKT^T308D,S473D^ transfection (Fig. S4F). Mesenchymal HCT116 p21^−/−^ cells failed to generate spheroids in the MDLC assay (Fig. S5C,D). Moreover, upon treatment with 50 or 100 μm SNAP for 5 days, p21^−/−^ HCT116 cells exhibited upregulated p‐AKT^S473^ expression without increasing CD133 levels (Fig. S4E). These data suggest that the stemness promoter NO regulates stemness and CD133 independent of the p‐AKT/p21 axis.

### 3.5 Cytoplasmic p21 promoted CSC characteristics in colorectal cancer cell lines

**Fig. 3:**
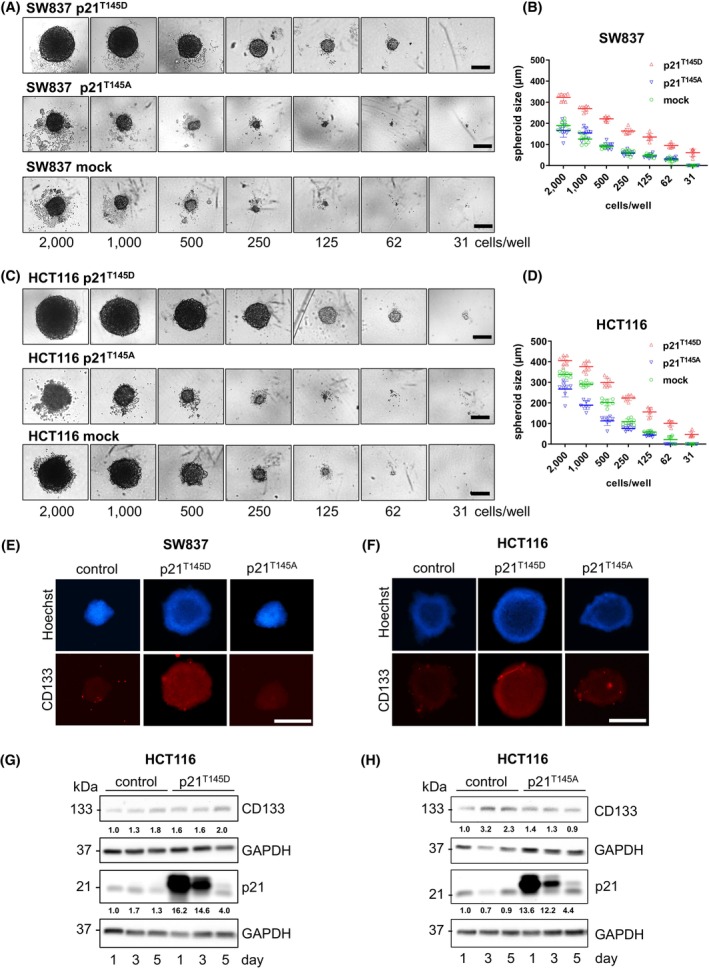
Cytoplasmic p21 promotes cancer stem cell phenotypes in colorectal cancer cell lines. (A, B) SW837 and (C, D) HCT116 colorectal cancer cell lines were transfected with hyperphosphorylated p21^T145D^ (cytoplasmic localized form of p21) or unphosphorylated p21^T145A^ (nuclear localized form of p21) and then subjected to a MCLD spheroid assay in serum‐free medium for 10 days, after which the diameter and number of spheroids were determined. The representative scale bars are 250 μm. Representative images and data of two independent experiments are shown. Statistical testing using a two‐way ANOVA revealed that p21^T145D^ spheroids were significantly larger than mock and p21^T145A^ transfected spheroids for both cell lines and for all conditions (*P* values were ≤ 0.05). (E) CD133 expression in SW837 and (F) HCT116 cells with hyperphosphorylated p21^T145D^ or unphosphorylated p21^T145A^ was evaluated by an immunofluorescence staining assay. After transfection with p21^T145D^ or p21^T145A^, 3D spheroids were generated by MCLD spheroid assay in serum‐free medium for 10 days. Then, the spheroids were fixed with 4% formaldehyde and stained with a mouse anti‐CD133 antibody, followed by an anti‐mouse Alexa Fluor 555‐conjugated antibody to visualize CD133 (red), and the nuclei were stained with Hoechst 33342 (blue). The representative scale bars are 500 μm. Representative images of two independent experiments are shown. (G, H) The expression of CD133 and p21 proteins after transfection of HCT116 cells with (G) hyperphosphorylated p21^T145D^ or (H) unphosphorylated p21^T145A^ was examined by western blotting. HCT116 cells were transfected with p21^T145D^ or p21^T145A^ for 1, 3, or 5 days, after which the expression levels of CD133 and p21 in the cells were evaluated and compared to those in the mock control cells. The blots were reprobed with GAPDH (1 : 50 000) to confirm equal loading of the samples. Representative blots of two independent experiments are shown.

To further analyze the special role of cytoplasmic p21 in maintaining and promoting the CSC phenotype in CRC, we transfected SW837 and HCT116 cells with two different plasmids, the phosphomimetic p21 form (p21^T145D^; which mimics the phosphorylated p21 wild‐type and results in cytoplasmic p21) or the unphosphorylated p21 form (p21^T145A^; nuclear p21) and determined their CSC characteristics. We confirmed our previous results [22] showing that the p21^T145D^ plasmid was mostly found in the cytoplasm while the p21^T145A^ plasmid was predominantly evident in the nuclear fraction as shown by western blot using a highly specific anti‐FLAG antibody (Fig. S6). The results of the MDCL assay (after 10 days) revealed that transfection with the p21^T145D^ construct increased the spheroid‐forming ability of both HCT116 and SW837 cells, whereas transfection with the p21^T145A^ construct decreased spheroid formation only in HCT116 cells (Fig. 3A–D); however, by day 5, these differences were no longer detected in SW837 cells, while minor differences persisted in HCT116 spheroids (Fig. S7A–D). Correspondingly, IF and western blot analyses indicated that transfection with the p21^T145D^ construct increased CD133 expression, while transfection with the p21^T145A^ construct decreased CD133 expression both in 3D spheroids (Fig. 3E,F) and in 2D culture (Fig. 3G,H). Notably, only the cytoplasmic form of p21 promoted the CSC phenotype in CRC cell lines.

To further identify cancer stem cell signaling pathways, we transfected SW837 cells with cytoplasmic p21 (p21^T145D^) or active AKT^T308D,S473D^ and analyzed stem cell markers using a Proteome Profiler Human Pluripotent Stem Cell array (Fig. S7E,F). First, p21^T145D^‐ and AKT^T308D,S473D^‐transfected cells promoted or suppressed the expression of stemness proteins in a very similar manner (Fig. S7E,F). In more detail, Oct3/4, Nanog, Sox2, E‐cadherin, APF, GATA4, HNF‐3β/FoxA2, PDX‐1/IPF1, Snail, VEGF R2/KDR/Flk‐1, and HCG were upregulated, while Otx2, TP63/TP73L, and GSC were downregulated in both transfected groups. Compared to the mock control group, Sox17 expression was specifically increased following transfection with AKT^T308D,S473D^, while it was reduced after transfection with p21^T145D^. These results underscore the critical role of the AKT‐cytoplasmic p21 axis in regulating CRC stemness.

### 3.6 The AKT/cytoplasmic p21 axis activates the NFκB pathway both *in vitro* and *in vivo*

Previous studies revealed that the activation of the NFκB pathway in cancer was associated with stemness both *in vitro* and *in vivo* [37]. Moreover, increased NFκB activity was correlated with increased tumor stage and poor prognosis in various types of cancer, including CRC [37]. Thus, we hypothesized that the CSC‐promoting role of cytoplasmic p21 might be associated with activation of the NFκB pathway.

**Fig. 4:**
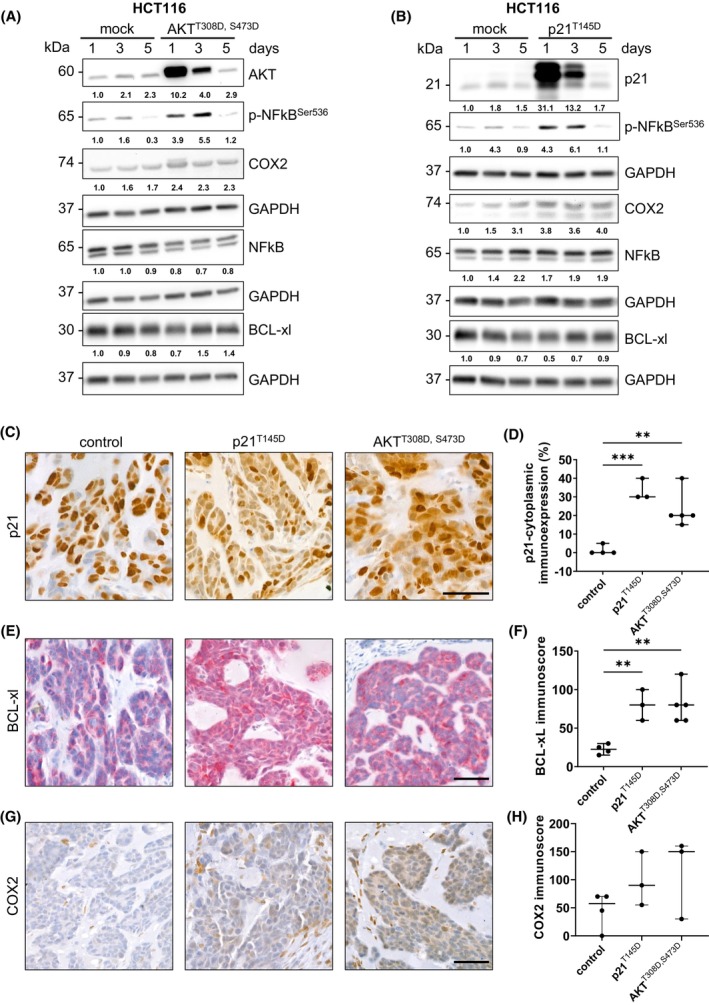
The AKT/cytoplasmic p21 axis activates the NFκB pathway both *in vitro* and *in vivo*. HCT116 cells were transfected with (A) hyperphosphorylated AKT^T308D,S473D^ or (B) hyperphosphorylated p21^T145D^ for 1, 3, or 5 days, after which the expression levels of AKT (A), p21 (B), NFκB, p‐NFκB (Ser536), COX2, and BCL‐xL were evaluated and compared to those of the mock control. (A, B) The blots were re‐probed with GAPDH (1 : 50 000) to confirm equal loading of the samples. Representative blots of two independent experiments are shown. (C–H) After transfection with hyperphosphorylated p21^T145D^ or hyperphosphorylated AKT^T308D,S473D^, SW837‐transfected cells were subjected to a chorioallantoic membrane (CAM) assay. Microtumors were harvested, and the expression of (C) cytoplasmic p21, (E) BCL‐xL and (G) COX2 was evaluated by immunohistochemistry. Representative images are shown (control, *n* = 4; p21^T145D^, *n* = 3; AKT^T308D,S473D^, *n* = 5). The representative scale bar is 50 μm for each marker. The immunoscores were determined for (D) cytoplasmic p21, (F) BCL‐xL and (H) COX2 (control, *n* = 4; p21^T145D^, *n* = 3; AKT^T308D,S473D^, *n* = 3 for COX2 and *n* = 5 for p21 and BCL‐xL; **P < 0.01, ***P < 0.001, parametric unpaired *t*‐test). Data are shown as median with 95% confidence interval.

To determine whether cytoplasmic p21 promoted NFκB activity, we transfected HCT116 cells with AKT^T308D,S473D^ or p21^T145D^ plasmids and examined NFκB‐related proteins by western blotting. We showed that both activated AKT and cytoplasmic p21 increased the expression of phosphorylated NFκB (S536) (p‐NFκB^Ser536^), while total NFκB levels were not altered (Fig. 4A,B). After 5 days, these differences disappeared (Fig. 4A,B). Similarly, the levels of COX2, a key downstream target of the NFκB pathway, were significantly elevated at all time points compared to the mock‐transfected cells (Fig. 4A,B). Furthermore, BCL‐xL, another downstream target of the NFκB pathway, showed a time‐dependent increase in the AKT^T308D,S473D^, and p21^T145D^ transfection groups, whereas its levels decreased over time in the mock‐transfected cells.

Next, SW837 cells were transfected with p21^T145D^ or AKT^T308D,S473D^ and subjected to CAM assays. Immunohistochemical staining for p21 revealed a substantial increase in cytoplasmic p21 signals in the cells transfected with p21^T145D^ and AKT^T308D,S473D^ (Fig. 4C,D). In parallel, the levels of the NFκB targets BCL‐xL and COX2 were greater in the transfected cells than in the control cells (Fig. 4E–H). These data suggest that cytoplasmic p21 plays an important role in NFκB pathway activation, which in turn promotes CSCs.

**Fig. 5:**
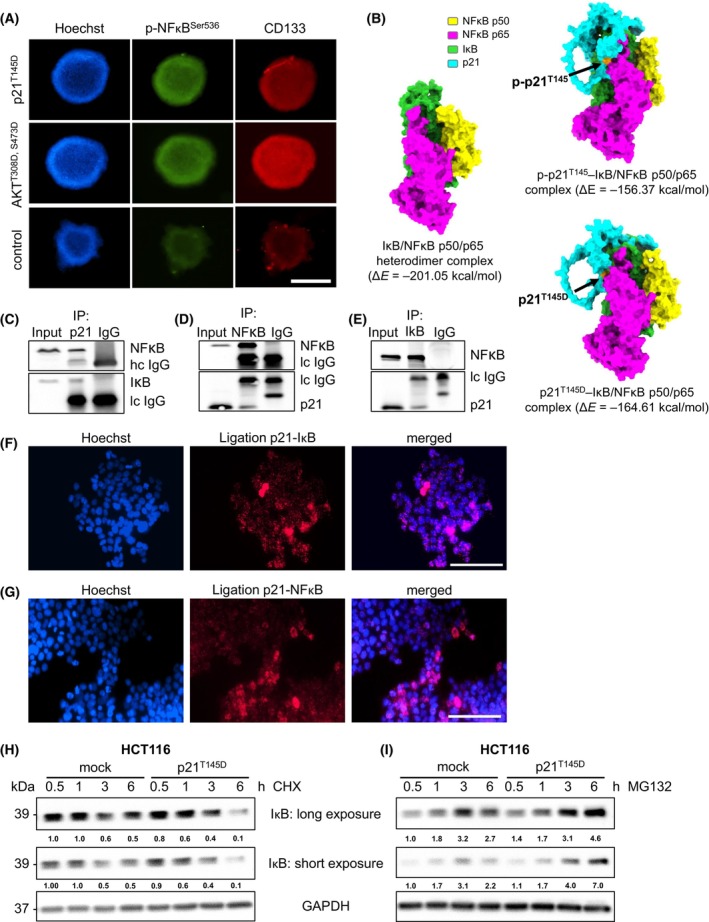
Interaction and destabilization of the NFκB‐IκB complex by p21. (A) p‐NFκB^S536^ and CD133 signals in HCT116 cells transfected with hyperphosphorylated p21^T145D^ or hyperphosphorylated AKT^T308D,S473D^ were assessed using an immunofluorescence staining assay. After transfection with p21^T145D^ or AKT^T308D,S473D^, 3D spheroids were generated via the MCLD spheroid assay in serum‐free medium for 10 days. The spheroids were then fixed with 4% formaldehyde and stained with rabbit anti‐p‐NFκB^S536^ antibody and mouse anti‐CD133 antibody, followed by Alexa Fluor 555‐conjugated anti‐rabbit antibody for CD133 (red) and anti‐mouse Alexa Fluor 488‐conjugated antibody for p‐NFκB^S536^ (green). Cell nuclei were visualized using Hoechst 33342 (blue). Representative scale bars are 500 μm. Representative images of two independent experiments are shown. (B) Computational modeling of protein–protein interactions between IκB/NFκB and p21 showed an increase in the free energy of the NFκB‐IκB complex (Δ*E* = −201.05 kcal·mol^−1^) upon interaction with the phosphorylated p21 molecule (p‐p21^T145^: Δ*E* = −156.37 kcal·mol^−1^; p21^T145D^: Δ*E* = −164.61 kcal·mol^−1^). Coimmunoprecipitation results (C) for p21, (D) NFκB, and (E) IκB demonstrated protein–protein interactions between p21 and NFκB, p21 and IκB, and NFκB and IκB, respectively. Representative blots of two independent experiments are shown. (F, G) Proximity ligation assays between (F) p21 and IκB, and (G) p21 and NFκB in the HCT116 cell line showed direct protein–protein interactions, indicated in red. Nuclei were visualized by Hoechst 33342 staining (blue). Representative scale bars are 100 μm. Representative images of two independent experiments are shown. (H) After transfection of HCT116 cells with p21^T145D^, cells were treated with 100 μg·mL^−1^ cycloheximide (CHX) for the indicated times. Cell lysates were collected for western blot analysis to evaluate IκB expression levels, with GAPDH (1 : 50 000) used as a loading control. Representative blots of two independent experiments are shown. (I) After transfection with p21^T145D^, HCT116 cells were treated with 10 μm MG132 for the indicated times. IκB expression levels were assessed by western blot analysis, with GAPDH (1 : 50 000) used to confirm equal loading. Representative blots of two independent experiments are shown.

Using 3D spheroids, AKT^T308D,S473D^ and p21^T145D^ transfection also led to simultaneous increases in p‐NFκB^Ser536^ and CD133 signals, as shown by immunofluorescence staining (Fig. 5A).

### 3.7 P21 destabilizes the NFκB‐IκB complex and promotes proteasomal degradation of IκB

As previously demonstrated, p21 functions as a scaffold protein, engaging in various protein–protein interactions that influence the activity and stability of its binding partners [22]. We modeled the p50 and p65 subunits to study protein–protein interactions with p21 and the corresponding binding energies as these subunits form the physiological dimer that interacts with IκB [38]. Through modeling the interaction between p21 and the IκB/NFκB complex, we observed that the total energies of the p21‐IκB/NFκB p65 complexes (p‐p21^T145^: Δ*E* = −156.37 kcal·mol^−1^; p21^T145D^: Δ*E* = −164.61 kcal·mol^−1^) were higher than that of the IκB/NFκB p65 complex alone (Δ*E* = −201.05 kcal·mol^−1^) (Fig. 5B). This suggests that p21 binding destabilizes the IκB/NFκB p65 heterodimer, thereby promoting NFκB activation (Fig. 5B). Since ERK2 is also known to phosphorylate cytoplasmic p21, we modeled the binding of p21 Thr57 and Ser130 into the IκB/NFκB complex (Fig. S8). However, the peripheral localization of the two phosphorylation sites in this complex make it very unlike that the ERK2 pathway interacts with the IκB/NFκB pathway.

As suggested by the *in silico* prediction model, we investigated protein–protein interactions using coimmunoprecipitation (co‐IP) and proximity ligation assays. Western blot analysis of the co‐IP experiments demonstrated that p21 interacts with NFκB (Fig. 5C,D) as well as with IκB (Fig. 5C,E). Furthermore, the proximity ligation assay confirmed that p21 interacts with the IκB‐NFκB complex (Fig. 5F,G). These results suggest that cytoplasmic p21 destabilizes the IκB‐NFκB complex through direct protein–protein interactions, thereby enhancing NFκB signaling. The activation of the NFκB pathway is strongly dependent on the proteasomal degradation of IκB [11]. To determine whether cytoplasmic p21 affects the control of IκB stability, we transfected HCT116 cells with the phosphorylated/cytoplasmic p21^T145D^ construct and simultaneously treated the transfected cells with 100 μg·mL^−1^ of the protein synthesis inhibitor cycloheximide (CHX) for the indicated durations (30 min, 1 h, 3 h, and 6 h). The IκB protein levels were determined by western blotting. Indeed, in transfected cells after CHX treatment for 6 h, the IκB protein signal nearly disappeared compared to that in the mock control group (Fig. 5H). This finding indicated that cytoplasmic p21 promoted the degradation of IκB. To investigate whether cytoplasmic p21 supports IκB degradation through the proteasomal pathway, we treated the transfected cells with 10 μm of the proteasome inhibitor MG132 (proteasome inhibitor) for the indicated durations (30 min, 1 h, 3 h, and 6 h). Western blotting revealed that after MG132 exposure for 6 h, the p21^T145D^‐transfected cells expressed more IκB than did the control cells (Fig. 5I). Cytoplasmic p21 significantly accelerated the degradation of IκB. By directly interacting with the IκB‐NFκB complex, cytoplasmic p21 promoted the complex's instability, leading to enhanced IκB degradation via the proteasomal pathway. This, in turn, activated the NFκB signaling pathway, thereby driving the CSC phenotype in CRC.

## 4 Discussion

Cancer stem cells (CSCs) are known to be the cause of all aggressive phenotypes of cancer, including chemoresistance, metastasis, and disease relapse, in multiple types of cancer [1, 2]. Thus, further elucidation of the molecular mechanisms regulating or promoting CSC phenotypes may lead to new therapeutic strategies to eradicate CSCs and overcome disease progression. Although cytoplasmic p21 has already been linked to drug resistance and survival [22, 39, 40], the cytoplasmic interacting partners or consequences of binding partner interactions are still unclear. For the first time, we identified a mechanism by which cytoplasmic p21 controls CSCs via upregulation of CD133 in CRC.

Activated AKT mediates the shuttling of p21 from the nucleus to the cytoplasm via the phosphorylation of p21 at threonine 145 (p‐p21^T145^) [24]. In particular, phosphorylation of p21 at T145 showed an increased p21 protein stability and promoted cell survival [41, 42]. Similarly, methylation at arginine 156 of p21 increased the cytoplasmic retention of p21 by facilitating its phosphorylation at the Thr145 position [43]. In parallel, ERK2 both phosphorylates p21 at Thr57 and Ser130 and thereby degrades p21 [44]. However, our *in silico* modeling did not support any involvement of the ERK2‐cytoplasmic p21 axis in CD133 stemness in colon cancer cells.

We confirmed that the co‐expression of activated AKT and p‐p21^T145^ in CRC cell lines was associated with high CSC phenotypes under 2D and 3D conditions. Our *in vitro* data were strongly supported by the immunohistochemical analysis of colon cancer patients, in which the cytoplasmic localization of p21 was correlated with advanced cancer stage and metastasis. Moreover, upon ectopic overexpression of activated AKT (AKT^T309D,S473D^) or inhibition of AKT phosphorylation by AKT inhibitors, we found that p‐p21^T145^ expression and spheroid formation capacity were strongly dependent on AKT activation. Interestingly, our data suggest that cytoplasmic p21 can also play an AKT‐independent role in CSC functions. Transfection with two different forms of p21, hyperphosphorylated p21^T145D^ and unphosphorylatable p21^T145A^, showed that p‐p21^T145D^ conferred CSC phenotypes, while unphosphorylatable p21 had the opposite effects. Accordingly, as recently reported, cytoplasmic p21 is a major hub in polyploid giant cancer cells and is one of the sources of CSCs [40]. When treating HCT116 cells with the stemness promoter NO, we observed an upregulation of CD133, but in a p21/p‐AKT‐independent manner. Surprisingly, SNAP treatment did not increase CD133 stemness in HCT116 p21^−/−^ cells, which might be explained by the fact that the HCT116 p21^−/−^ cell line is mesenchymal in nature and categorized as CMS4 subtype [45]. Obviously, the loss of p21 led to a significantly different gene signature and cannot be used for stemness induction via SNAP.

The second major part of our study was the identification of interacting candidate proteins to elucidate the signaling pathway downstream of cytoplasmic p21 involved in CSC promotion. In a previous study, we identified pChk2 as a direct interacting partner of cytoplasmic p21 that confers resistance to the damaging therapeutic agent 5‐FU [22]. We focused on NFκB since its pathway is involved in proliferation and survival, linking tumorigenesis with inflammation. Thus, the NFκB pathway should have an essential function in stemness, and the inhibition of NFκB signaling would effectively reduce stemness and self‐renewal capacity. NFκB is negatively regulated by IκB in an inhibitory complex and can be activated by IκB kinase‐dependent (IκK‐dependent) phosphorylation and subsequent degradation of IκB proteins [46]. We discovered that cytoplasmic p21 promoted the degradation of IκB through the proteasomal pathway. Although p21 has no enzymatic activity, *in silico* modeling revealed that the p21^T145D^ form exhibited a direct interaction with the NFκB‐IκB complex, destabilizing the complex by increasing the total binding energy. This *in silico* prediction was verified by coimmunoprecipitation and proximity ligation assays. We suggest that the p21^T145D^ form can promote the proteasomal degradation of IκB, leading to an increase in NFκB activity. Activation of the NFκB pathway increased the expression of downstream targets of NFκB, such as BCL‐xL and COX2, *in vitro* and *in vivo* in CAM ovografts. BCL‐xL‐mediated apoptosis resistance is one of the major hallmarks of CSCs, and colon tumor cells generally exhibit high basal levels of this pro‐survival protein [47]. Accordingly, cytoplasmic p21 in trophoblastic giant cells was shown to protect against cell death [39]. In breast cancer, BCL‐xL regulates the CSC phenotype via the RAS signaling pathway [48]. The inhibition of BCL‐xL has been suggested to be a promising target for eliminating quiescent CSCs in lung cancer [49]. Recently, the benefit of combination therapy with BCL‐xL inhibitors and BRAF inhibitors in CRC patients was demonstrated [50]. Furthermore, cytoplasmic re‐localization of p21 increased the expression of BCL‐xL in doxorubicin‐resistant breast cancer cells [51]. Similarly, inhibition of COX2 expression by the transcriptional repressor FOXP3 decreased CSC self‐renewal in CRC [52]. COX2 mediates HIF‐1α‐dependent rebuilding of the inflammatory tumor microenvironment [53].

## 5 Conclusion

The subcellular localization of p21 is crucial in both normal cell function and cancer. Nuclear p21 inhibits cell cycle progression and promotes apoptosis, while cytoplasmic p21 is linked to oncogenic activities such as chemoresistance, proliferation, and metastasis. These cancer traits driven by cytoplasmic p21 are associated with CSC properties, although the underlying mechanisms remain unclear. In our study, we identified cytoplasmic p21 as a key regulator of CSC characteristics in CRC, promoting NFκB pathway activation by destabilizing the NFκB‐IκB complex through protein–protein interactions, leading to IκB degradation. Two NFκB targets, BCL‐xL and COX2, were identified as novel downstream effectors of cytoplasmic p21, closely associated with the CSC phenotype in CRC. Our mechanistic study offers new insights into how cytoplasmic p21 may drive the CSC phenotype in colorectal cancer. Screening for the subcellular localization of p21 could help identify patients at high risk for metastasis.

## Conflict of interest

The authors declare no conflict of interest.

## Author contributions

AM, PC, and RS‐S initiated and conceptualized the study. AM and RS‐S designed the experiments. AM, CH, PC, and KH performed the experiments. BN performed the structural modeling analysis. AV‐R, KE‐W, and AH performed and analyzed the immunohistochemical staining. AM, KH, and RS‐S analyzed, interpreted, and discussed the results. AM prepared the first draft of the manuscript. AM, DT, and KH performed the additional experiments for the revision. KH integrated the data into the manuscript and ensured all requirements for the figures and the text were met. AM, PC, KH, and RS‐S revised the manuscript. AM and KH controlled original data archiving. All authors read and approved the final manuscript.

## Supporting information


**Fig. S1.** Subcellular fractionation of HCT116 and HT29 cells grown in 2D and 3D conditions.
**Fig. S2.** Multicellular limiting dilution spheroid assay of transfected hyperphosphorylated AKT cell for 5 days of assay duration.
**Fig. S3.** Wortmannin treatment increased nuclear p21.
**Fig. S4.** Nitric oxide promoted cancer stem cell phenotypes in HCT116 cells in a p21‐dependent manner.
**Fig. S5.** Cancer stem cell properties of HCT116 p21^−/−^ cells.
**Fig. S6.** Transfection of HCT116 cells with p21^T145D^ and p21^T145A^ induced cytoplasmic and nuclear localization of p21, respectively.
**Fig. S7.** Effect of hyperphosphorylated p21^T145D^ and unphosphorylated p21^T145A^ on cancer stem cell properties.
**Fig. S8.** Computational modelling of ERK2‐mediated phosphorylation of p21 and its interaction with IκB/NFκB p50/p65 complex.
**Table S1.** Patient characteristics–comparison with cytoplasmic p21.

## Data Availability

The original western blot data of this study are available in the Supporting Information of this article. All data regarding the modeling are available upon request. The structural data that support these findings are openly available in the wwPDB at https://doi.org/10.2210/pdb1IKN/pdb. More detailed data about Materials and methods are available upon request of the authors.
