# Supplementary material for: Cytoplasmic p21 promotes stemness of colon cancer cells via activation of the NFκB pathway
Source: Mol Oncol. 2025 Nov 3;20(4):1022–40. doi: 10.1002/1878-0261.70150 (PMC13060645; doi:10.1002/1878-0261.70150)

Fig 1E-western blot in manuscript

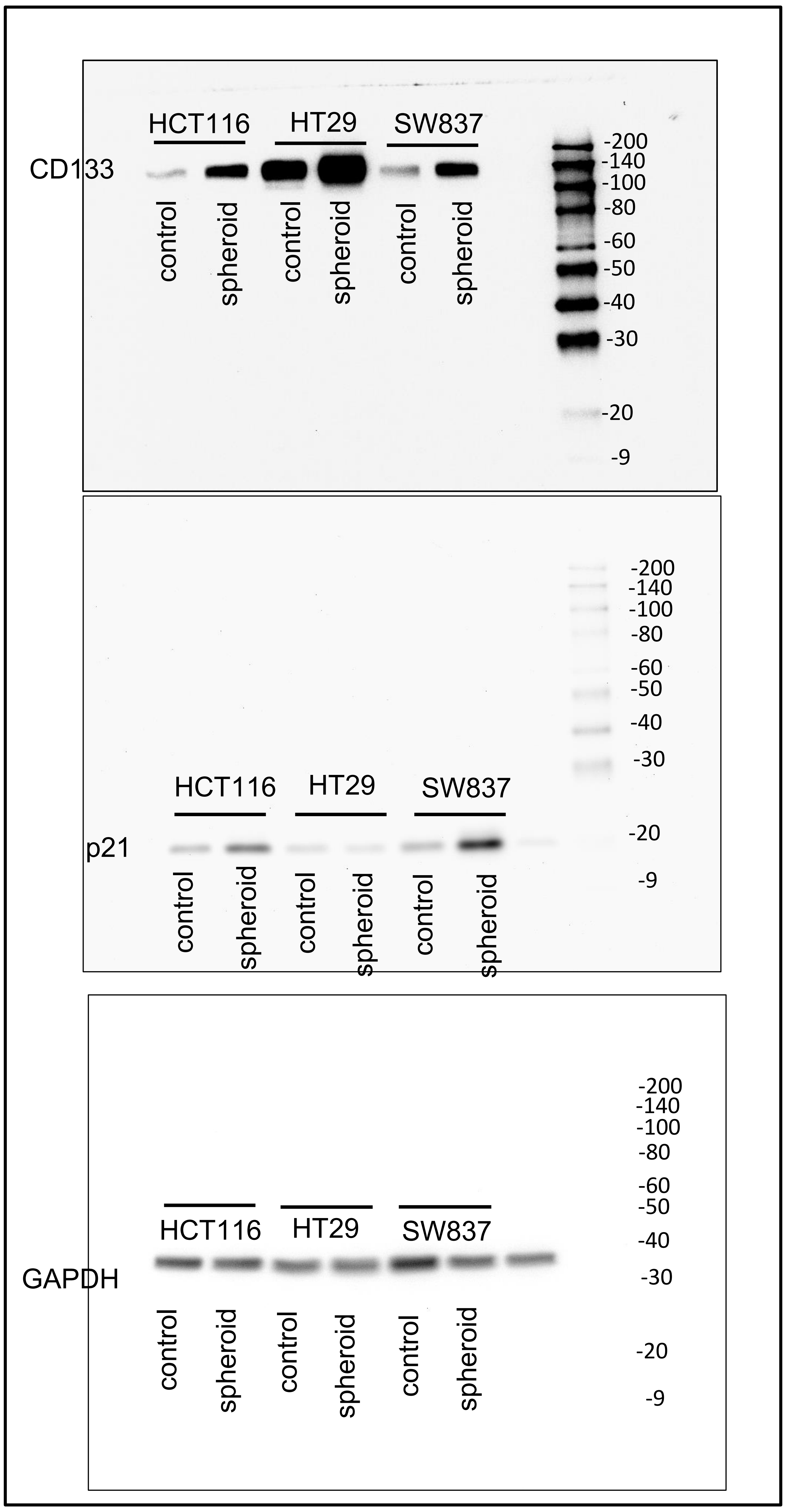

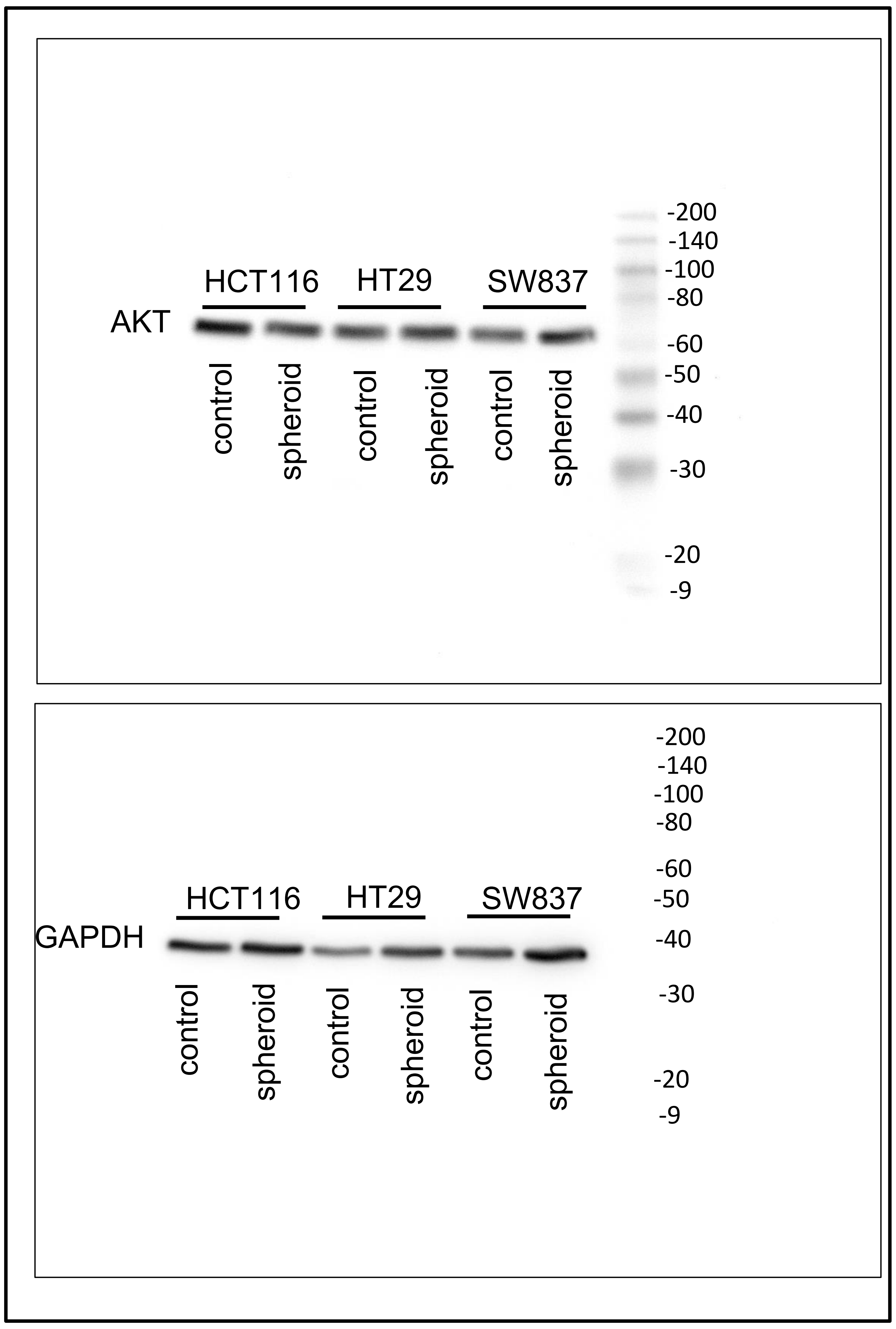

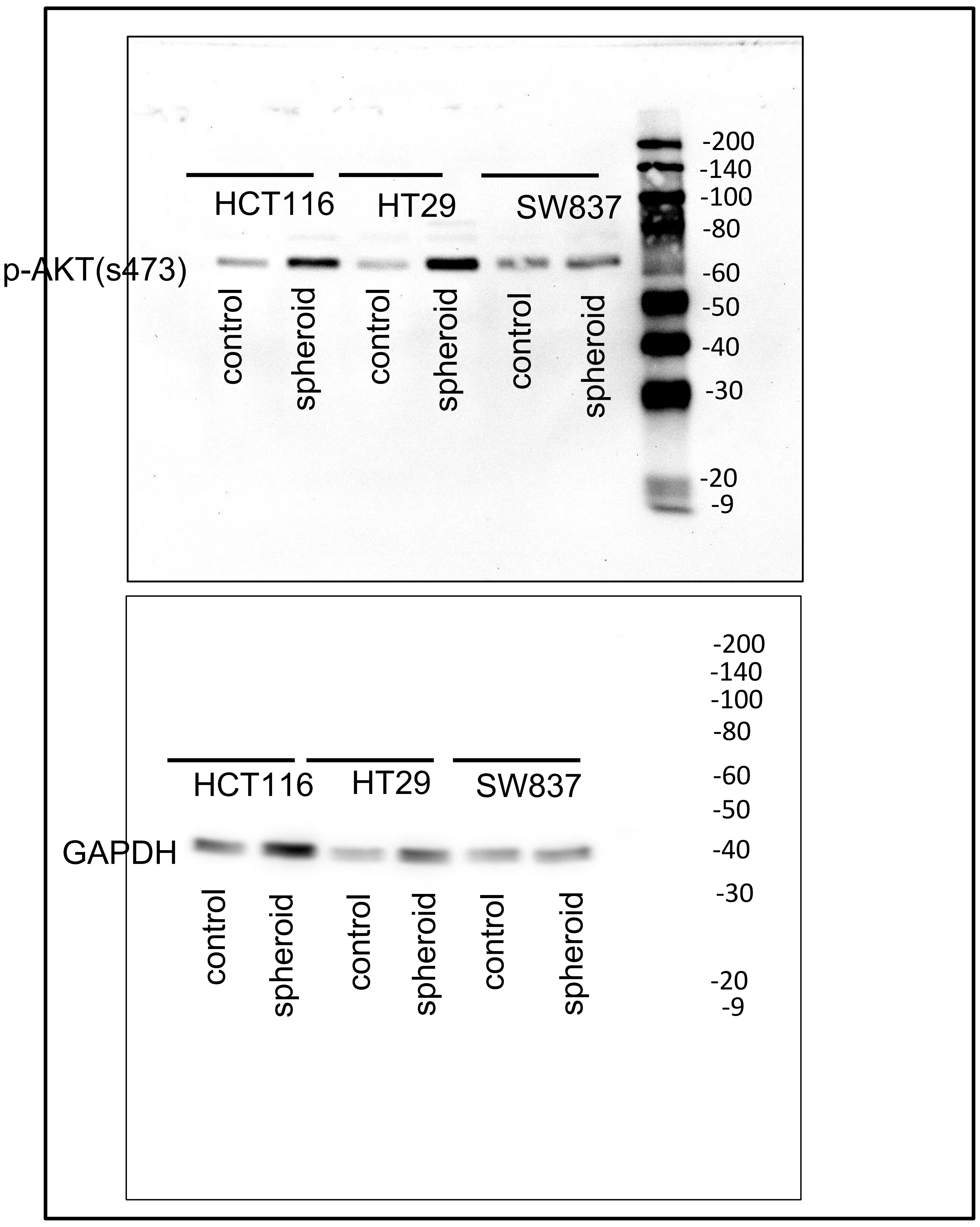

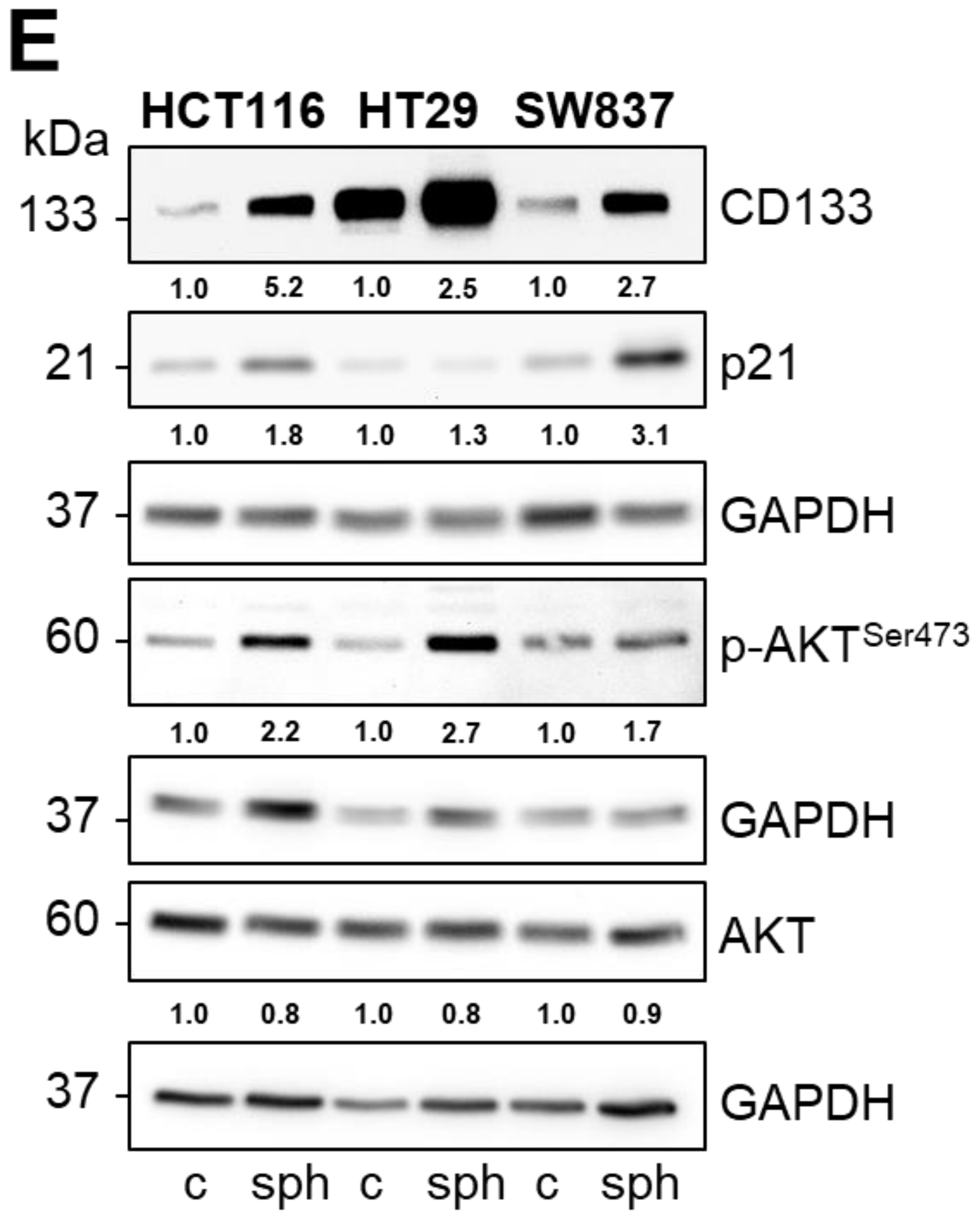

Fig 2E-western blot in manuscript

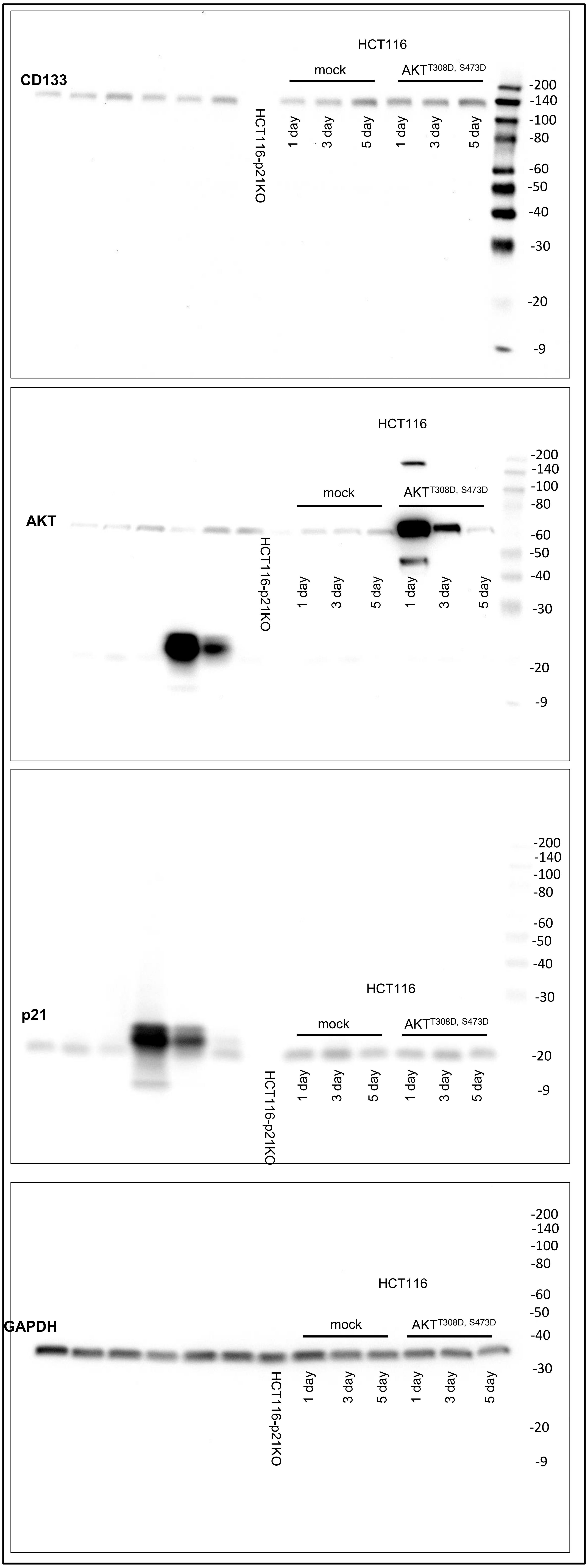

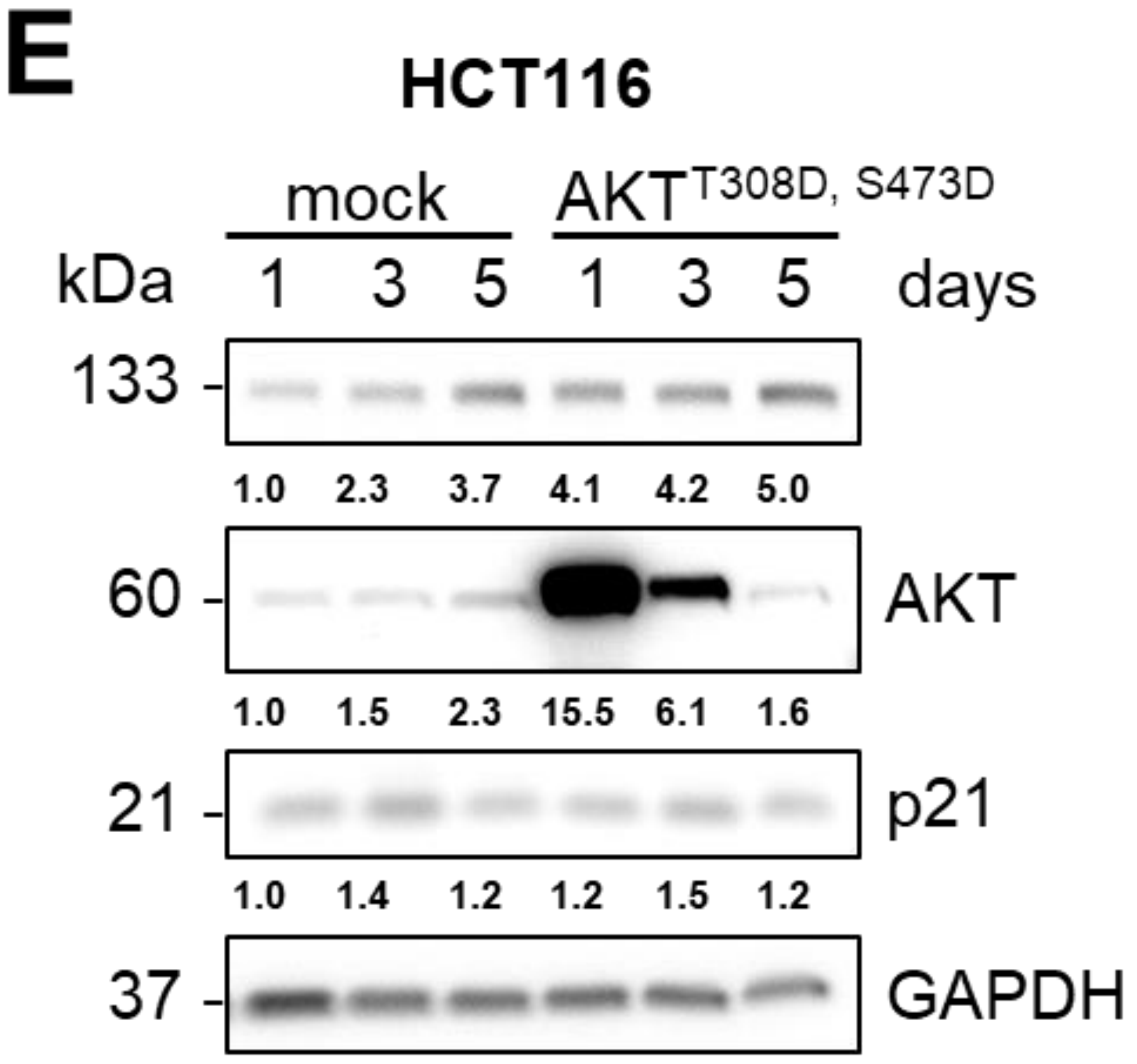

Fig 2H-western blot in manuscript

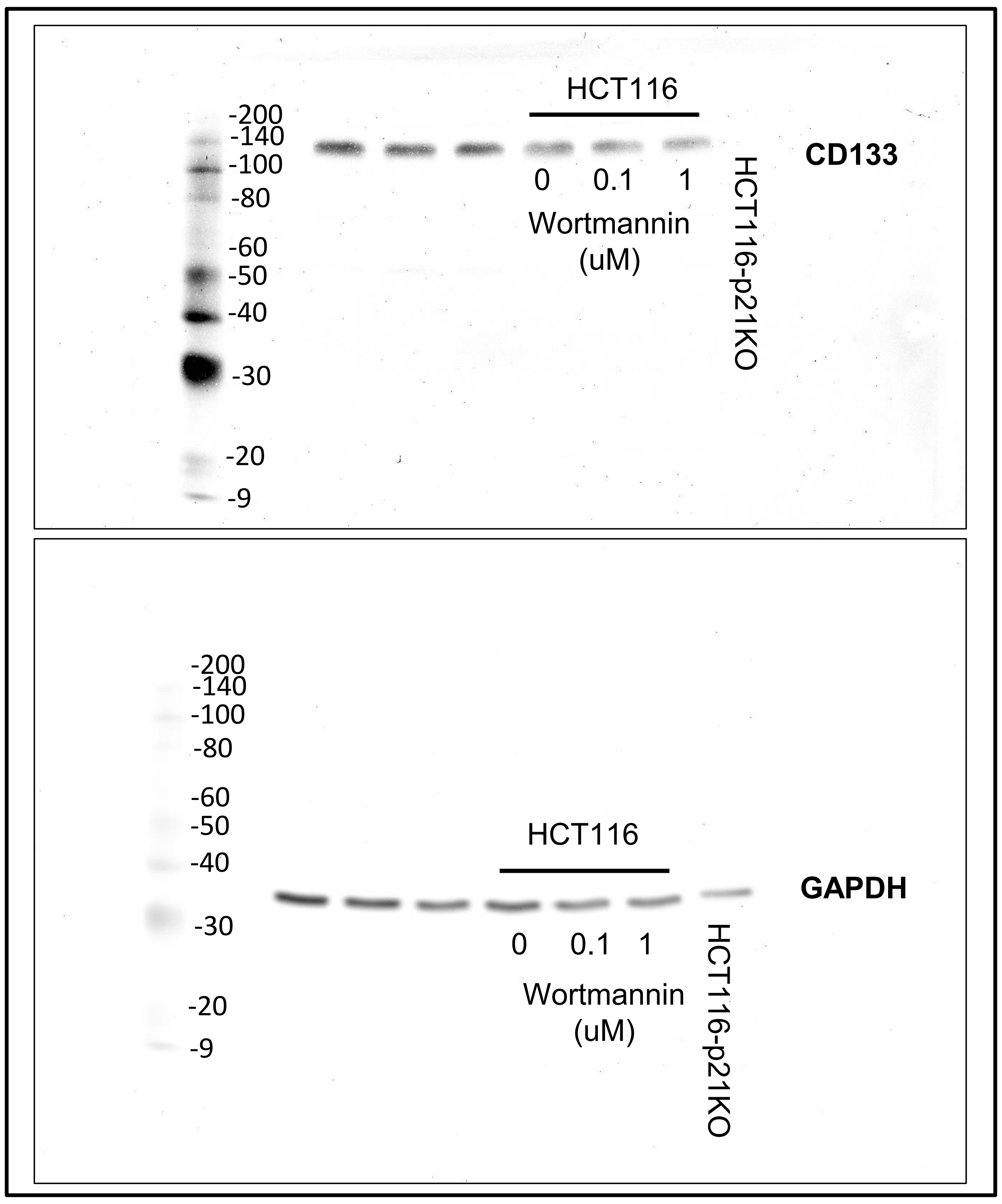

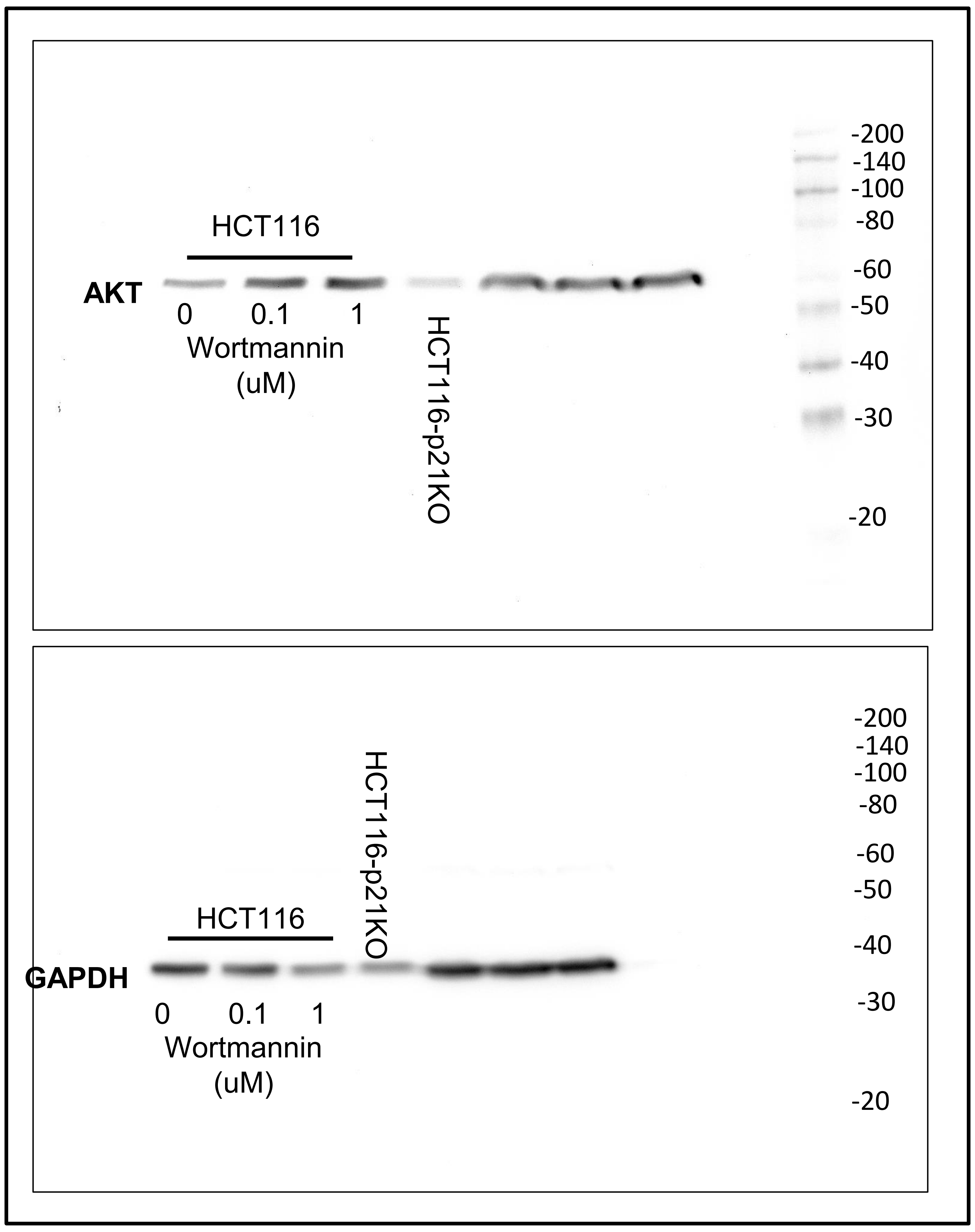

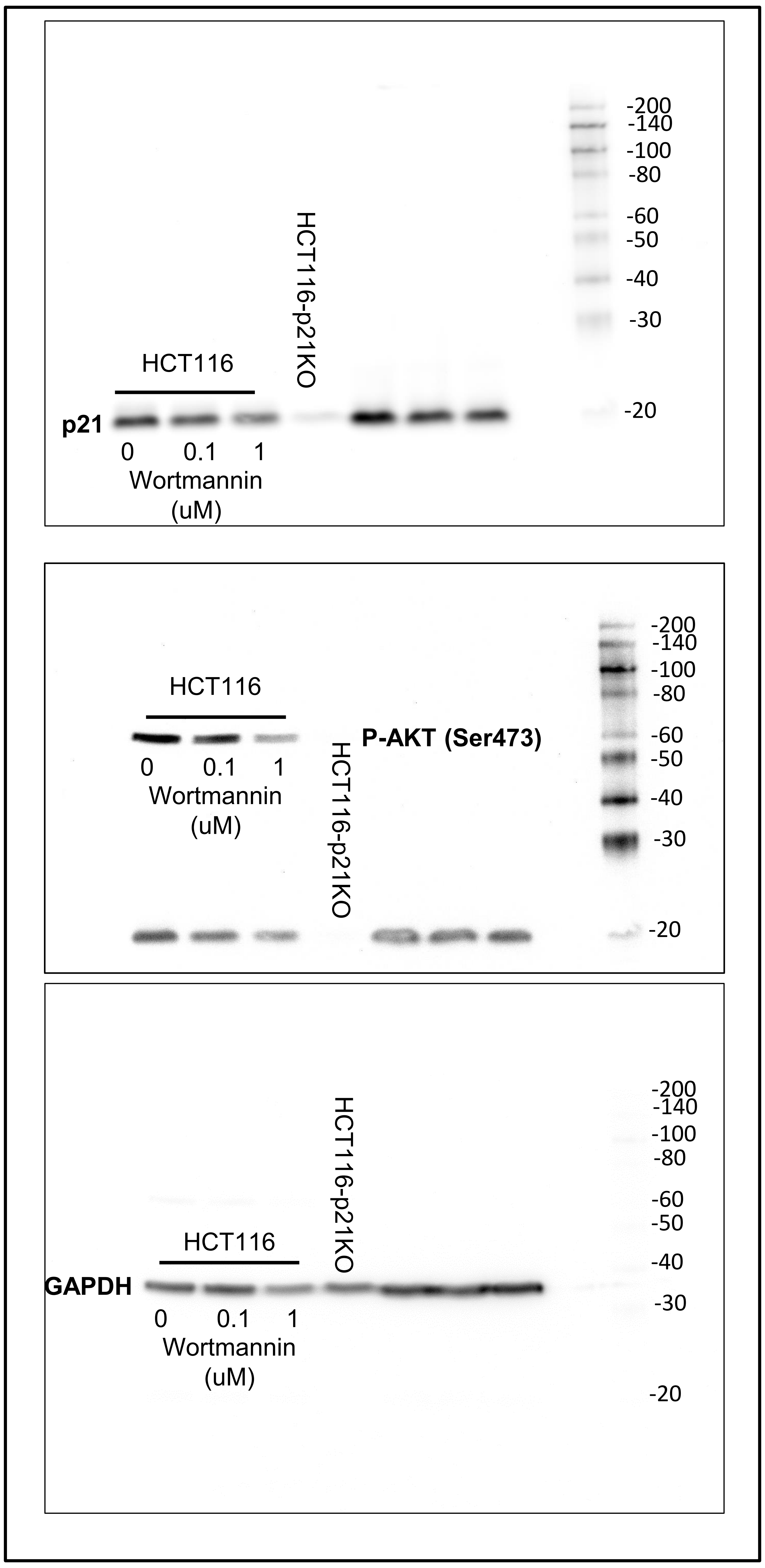

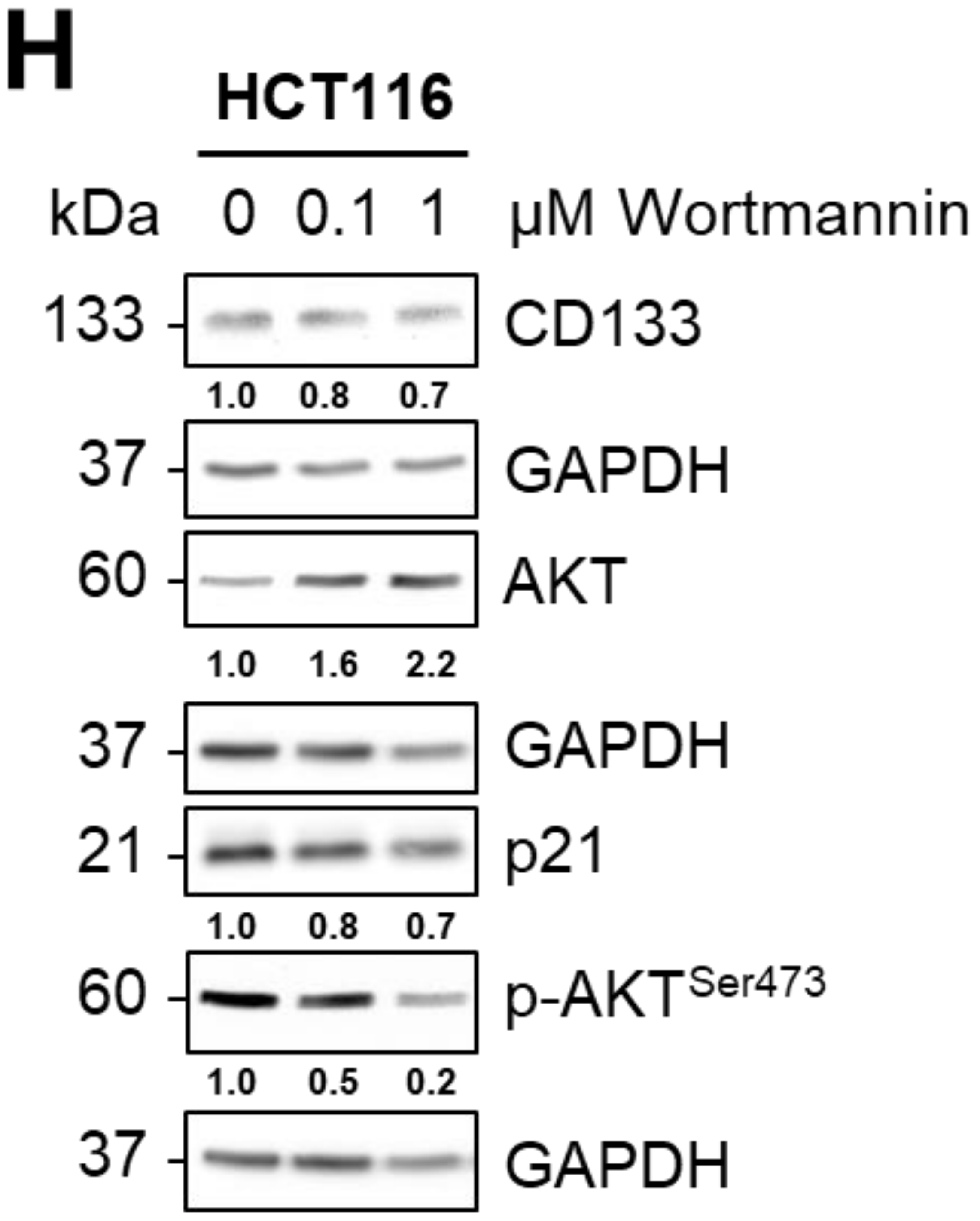

Fig 2I-western blot in manuscript

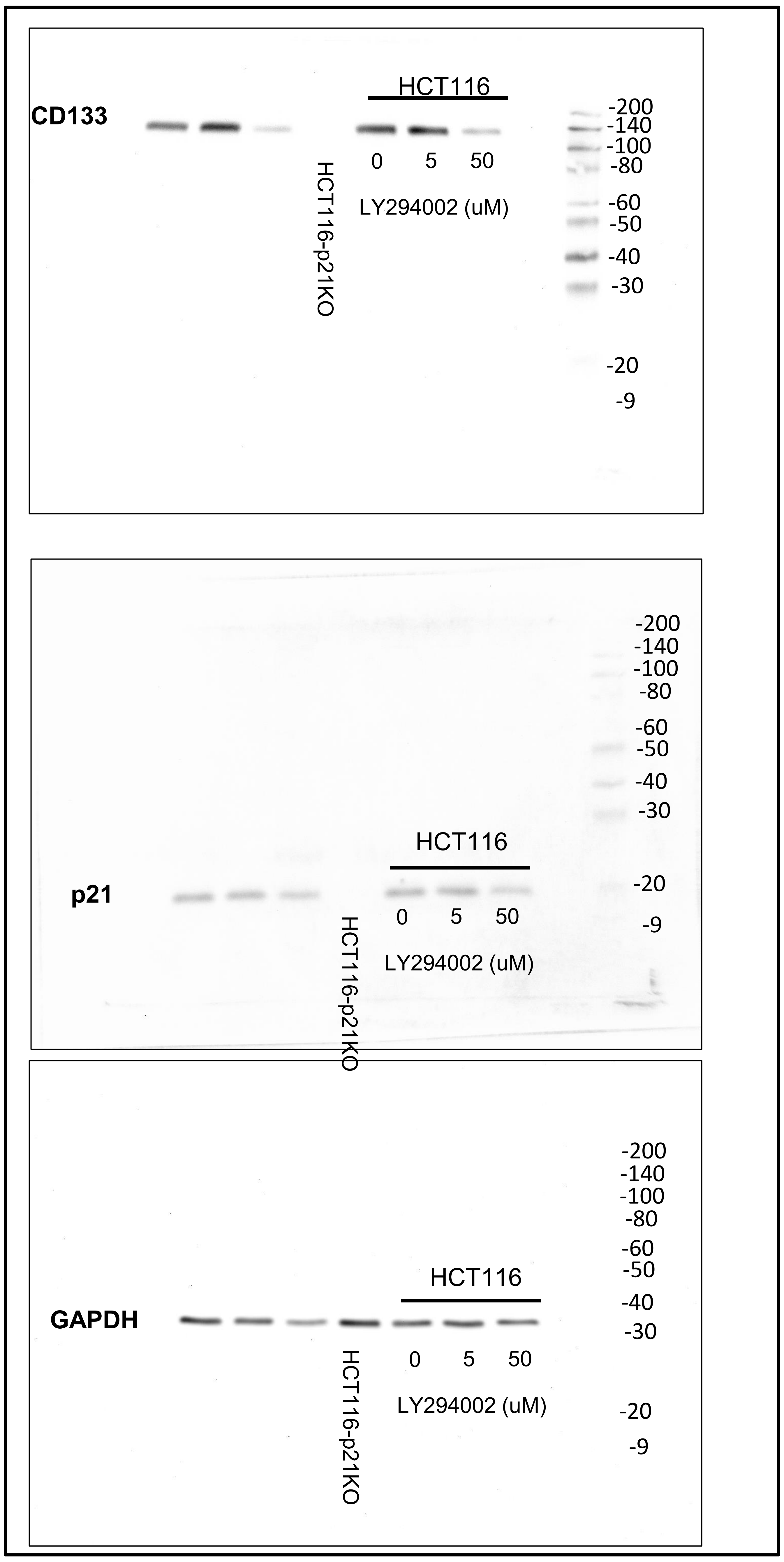

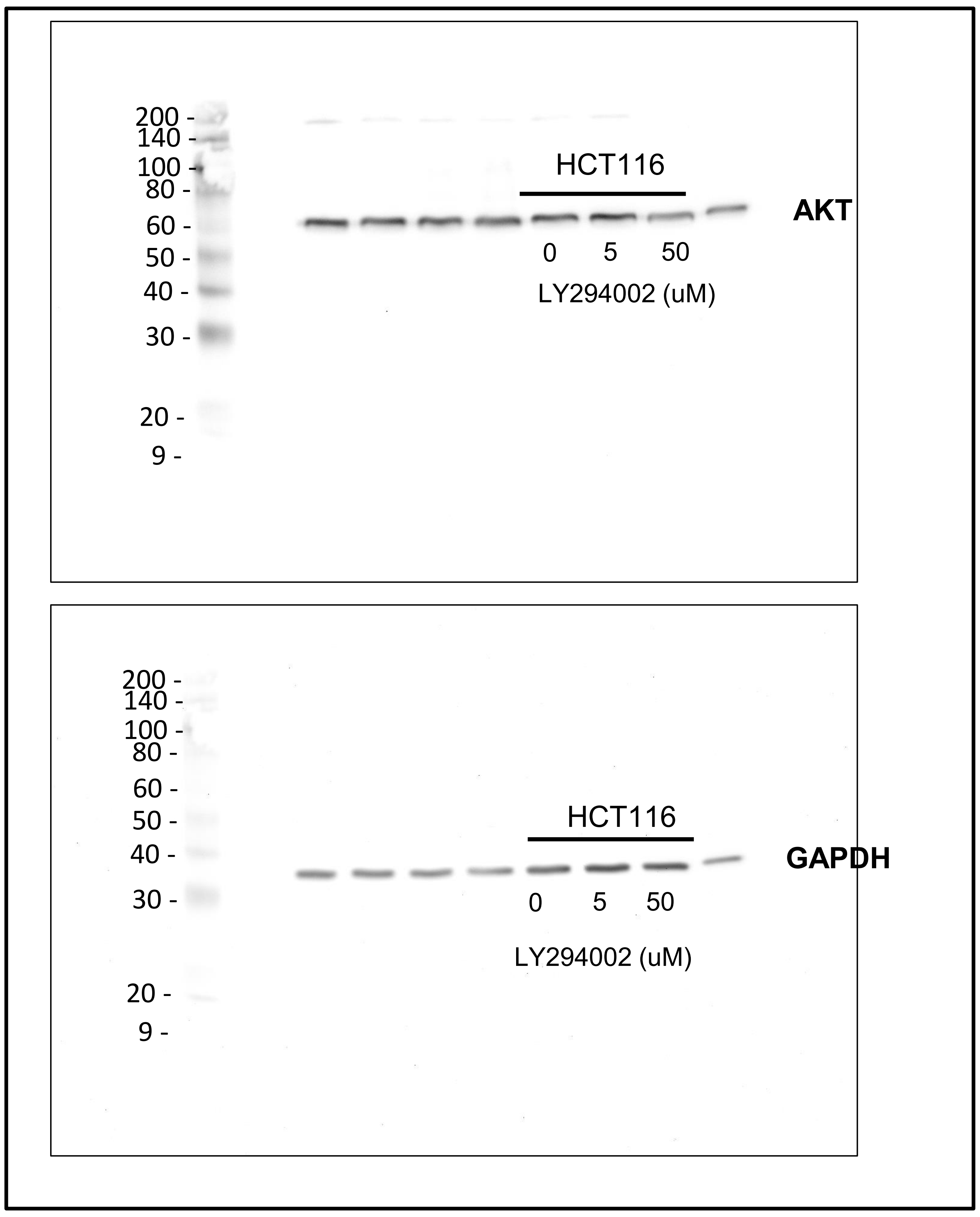

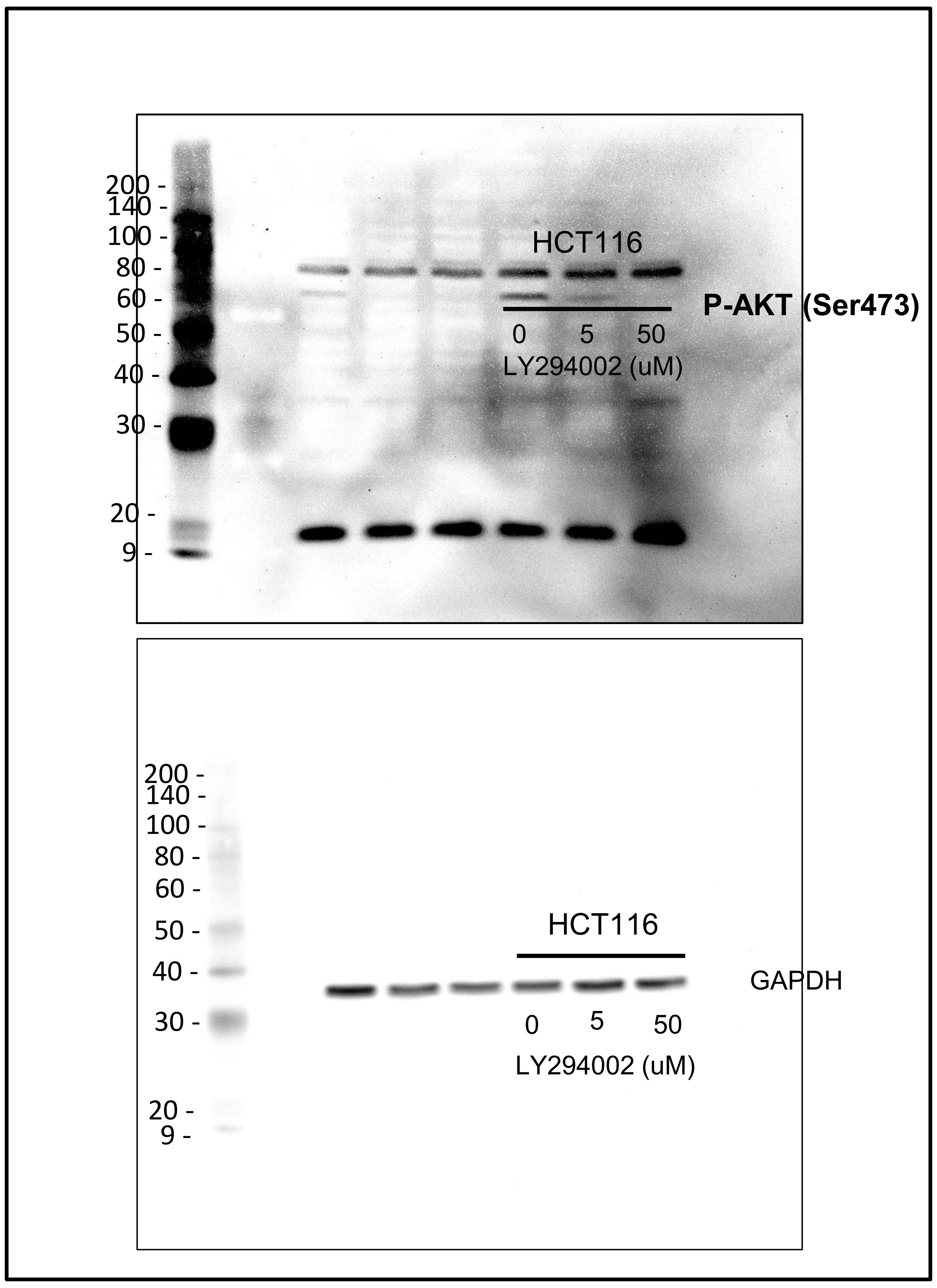

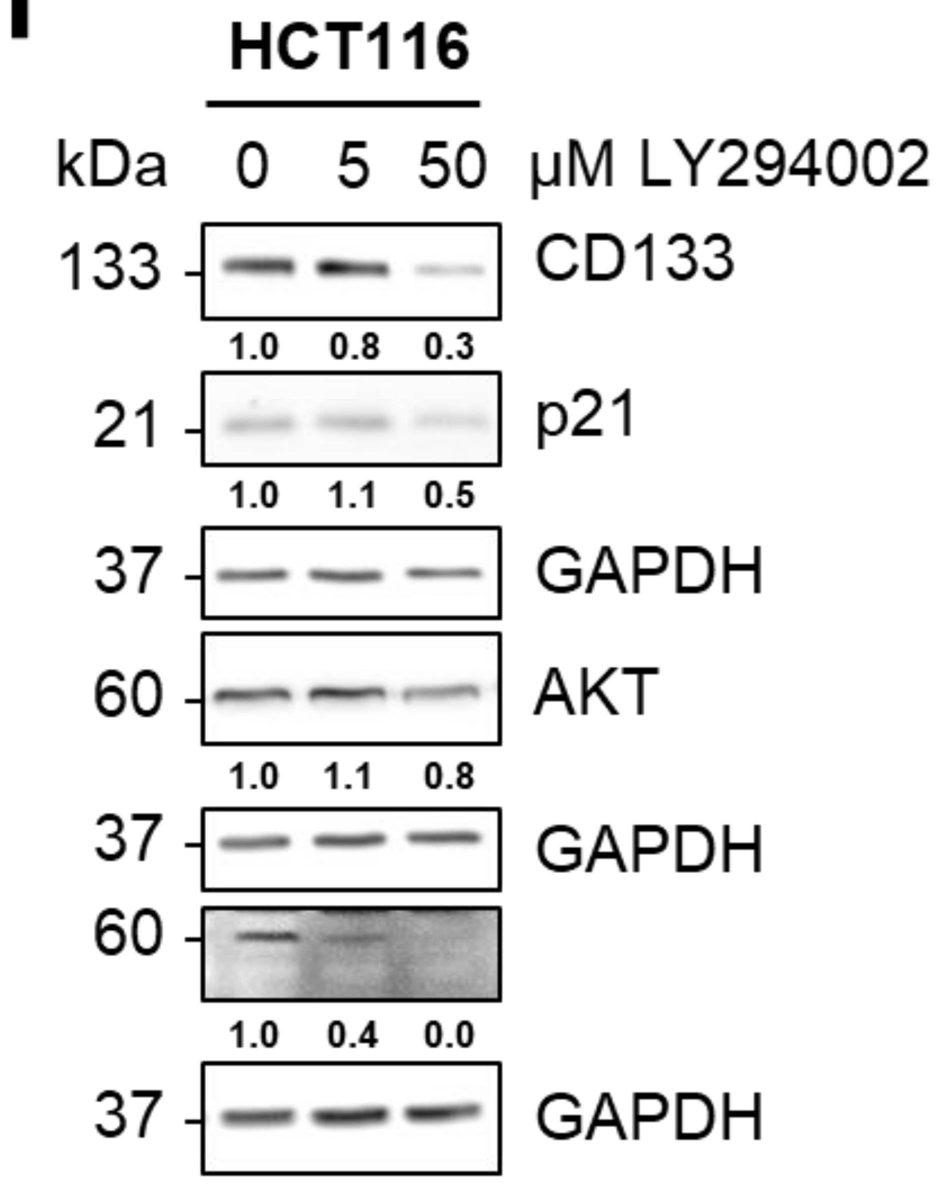

Fig 3G-western blot in manuscript

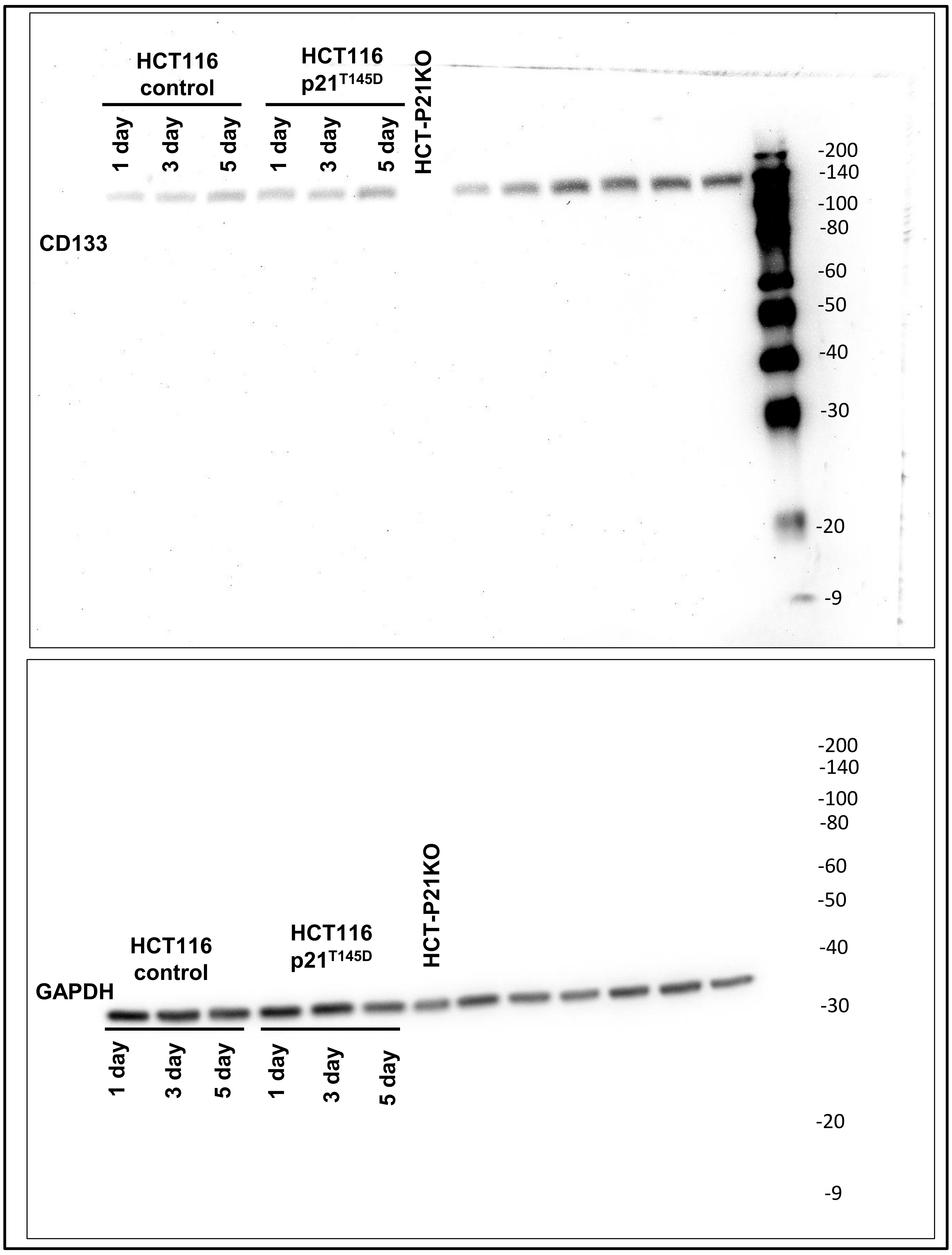

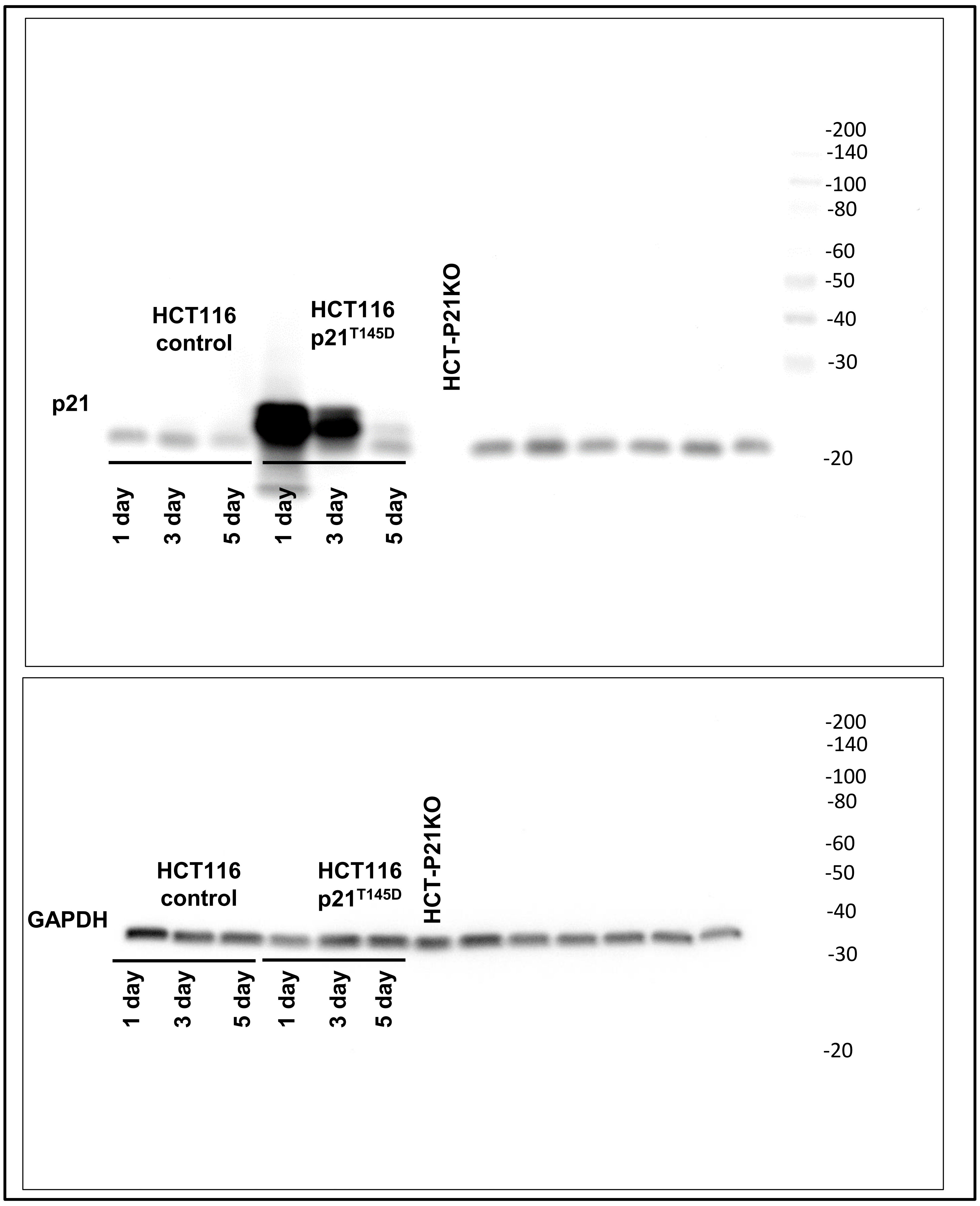

G

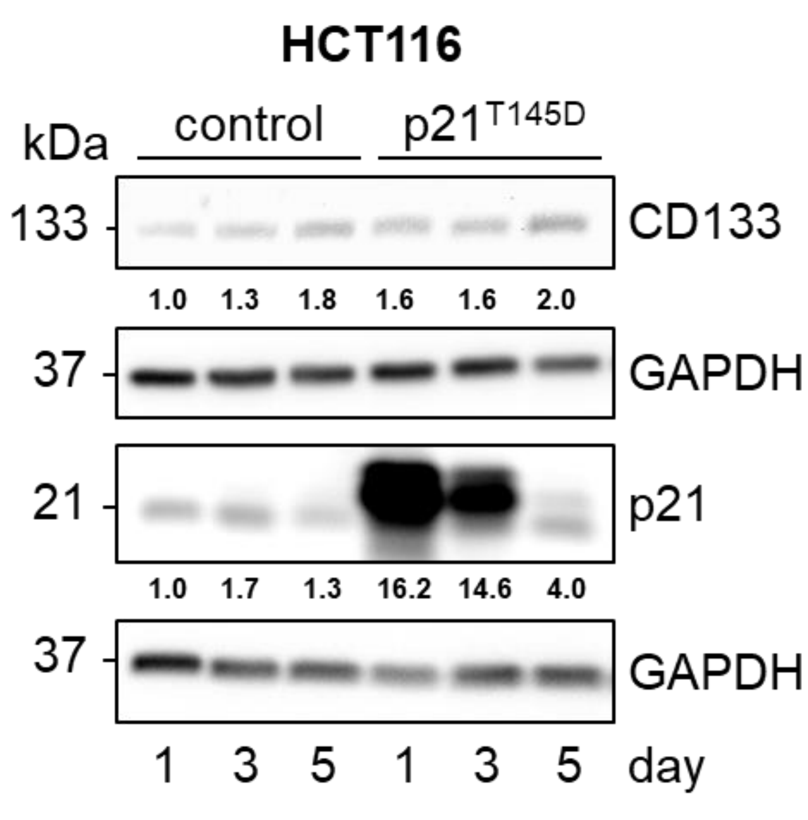

Fig 3H-western blot in manuscript

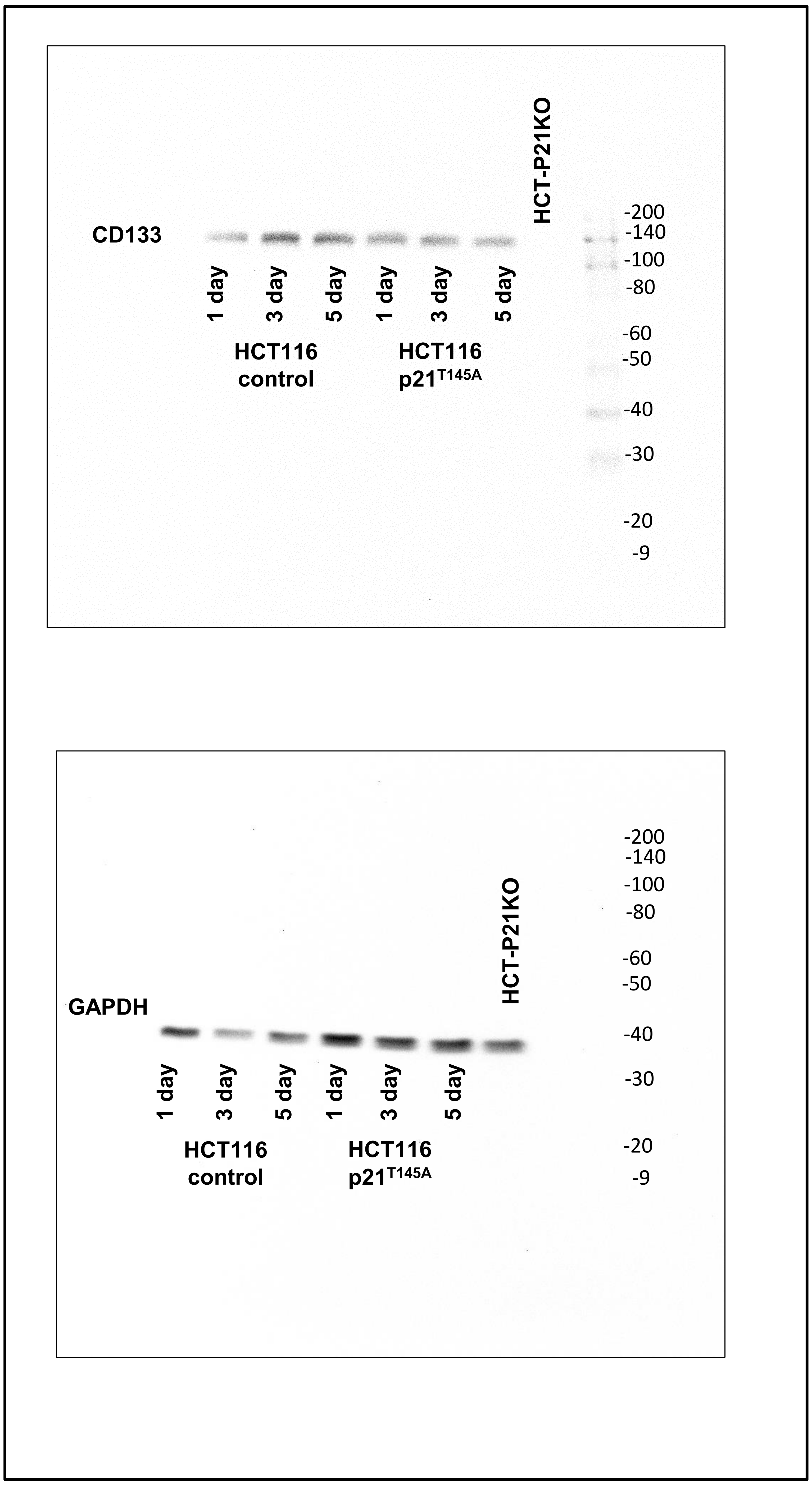

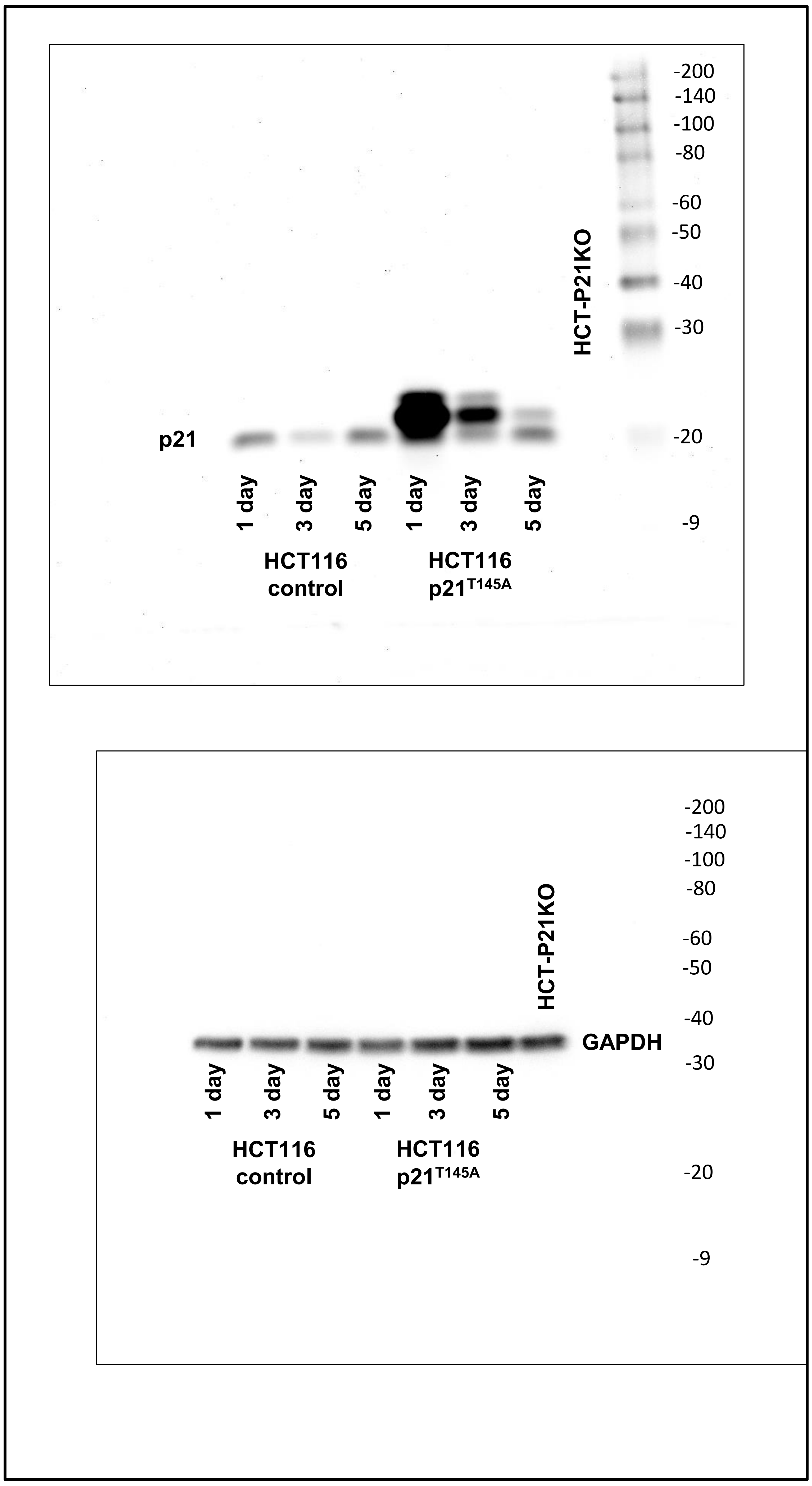

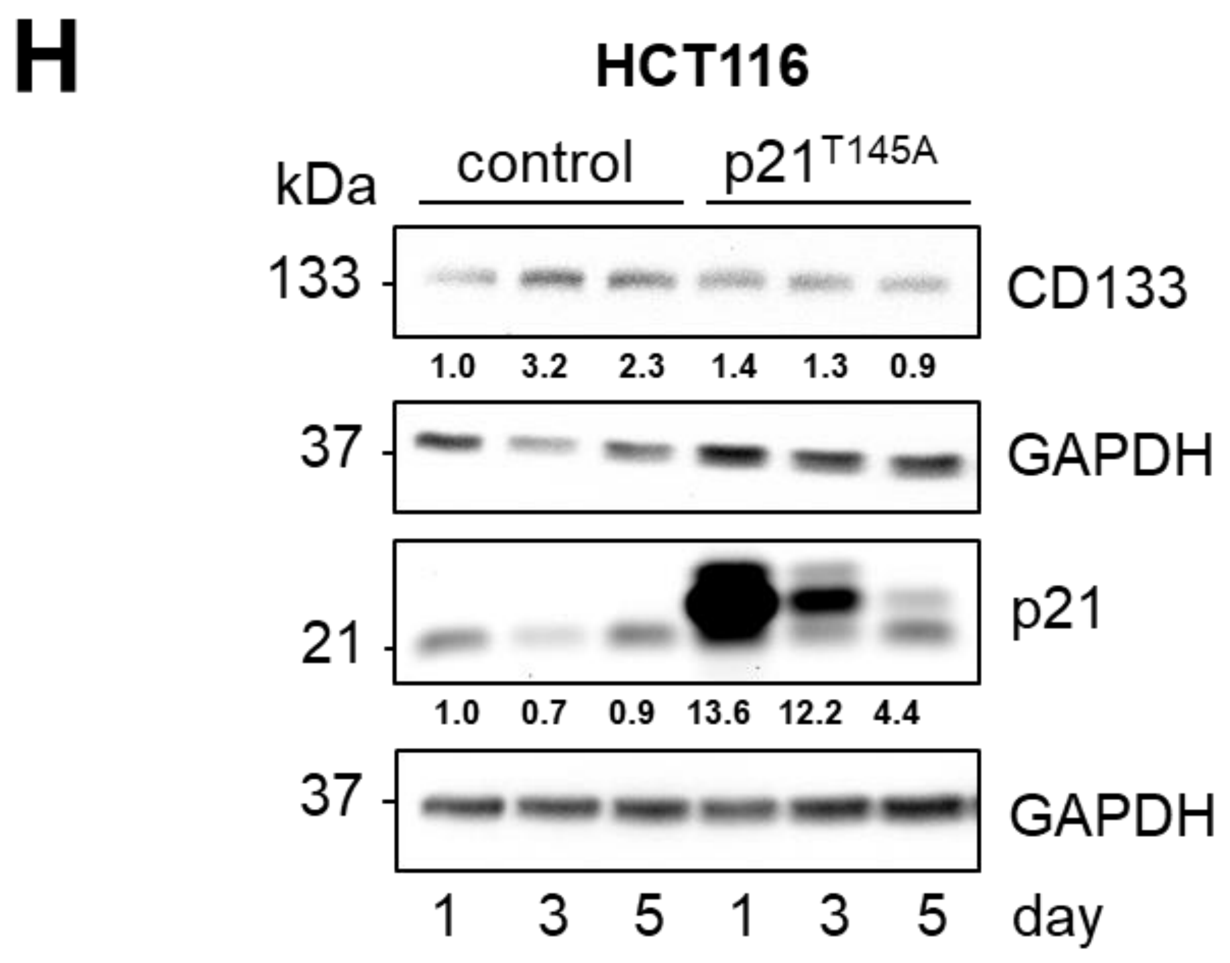

Fig 4A-western blot in manuscript 1/2

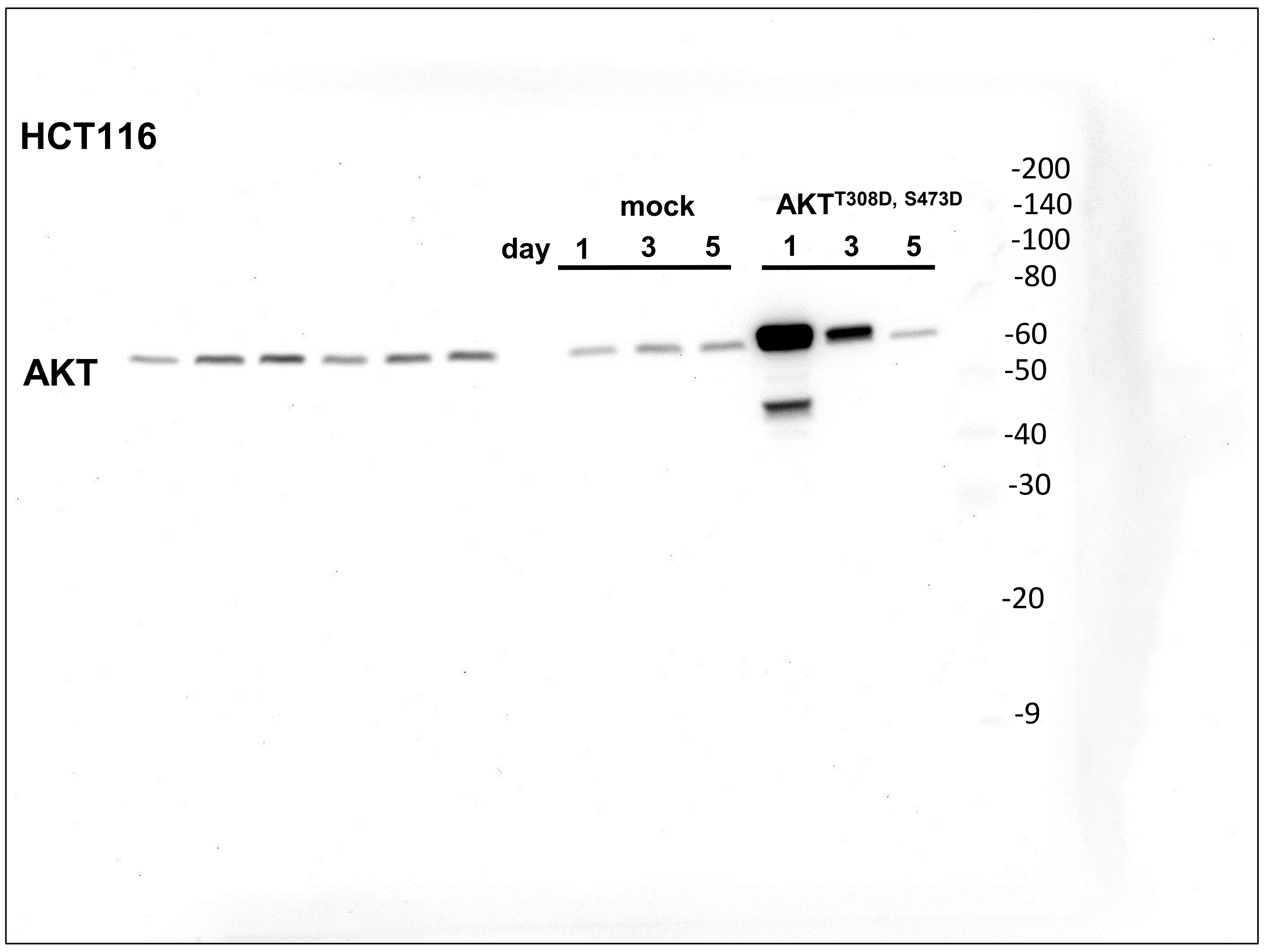

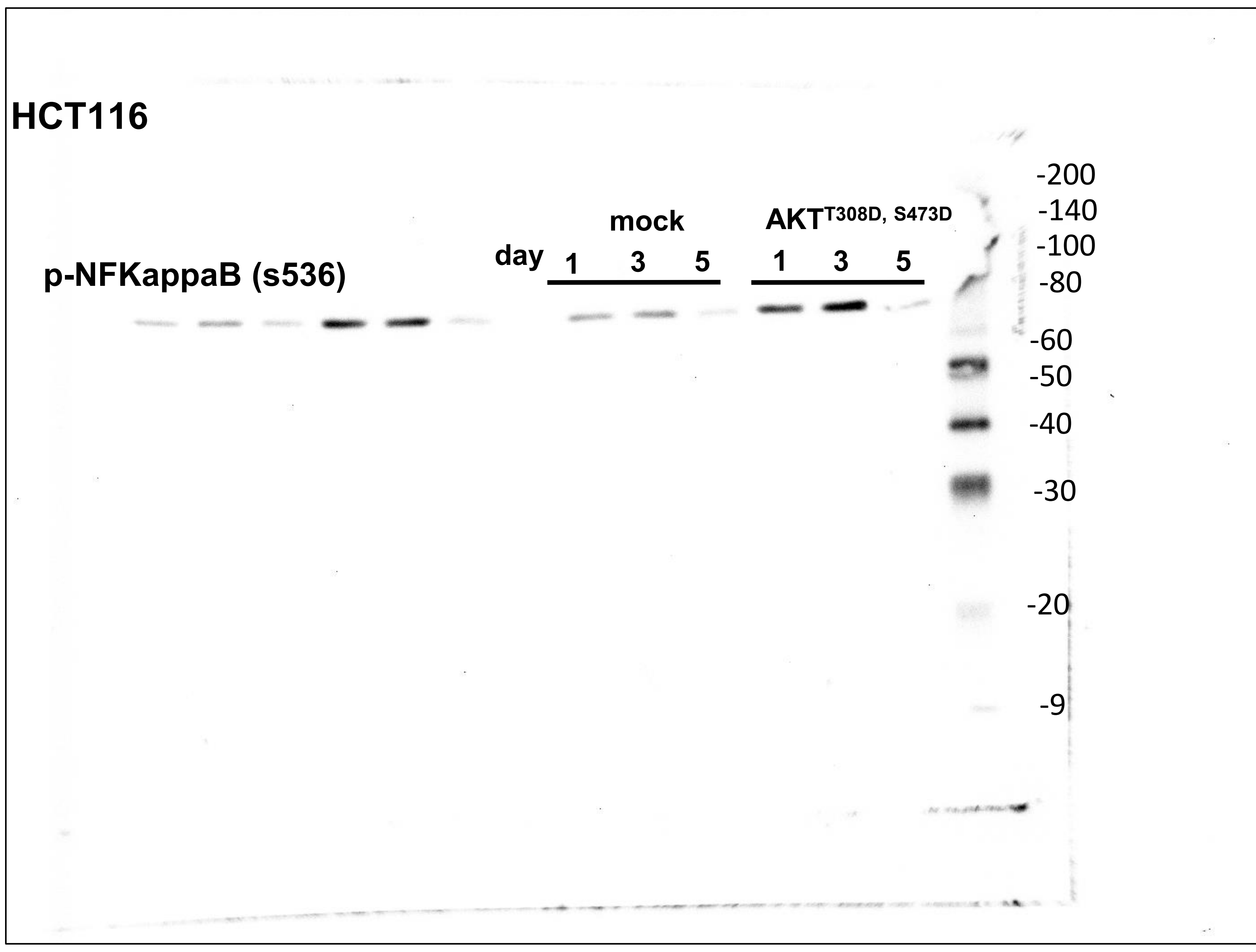

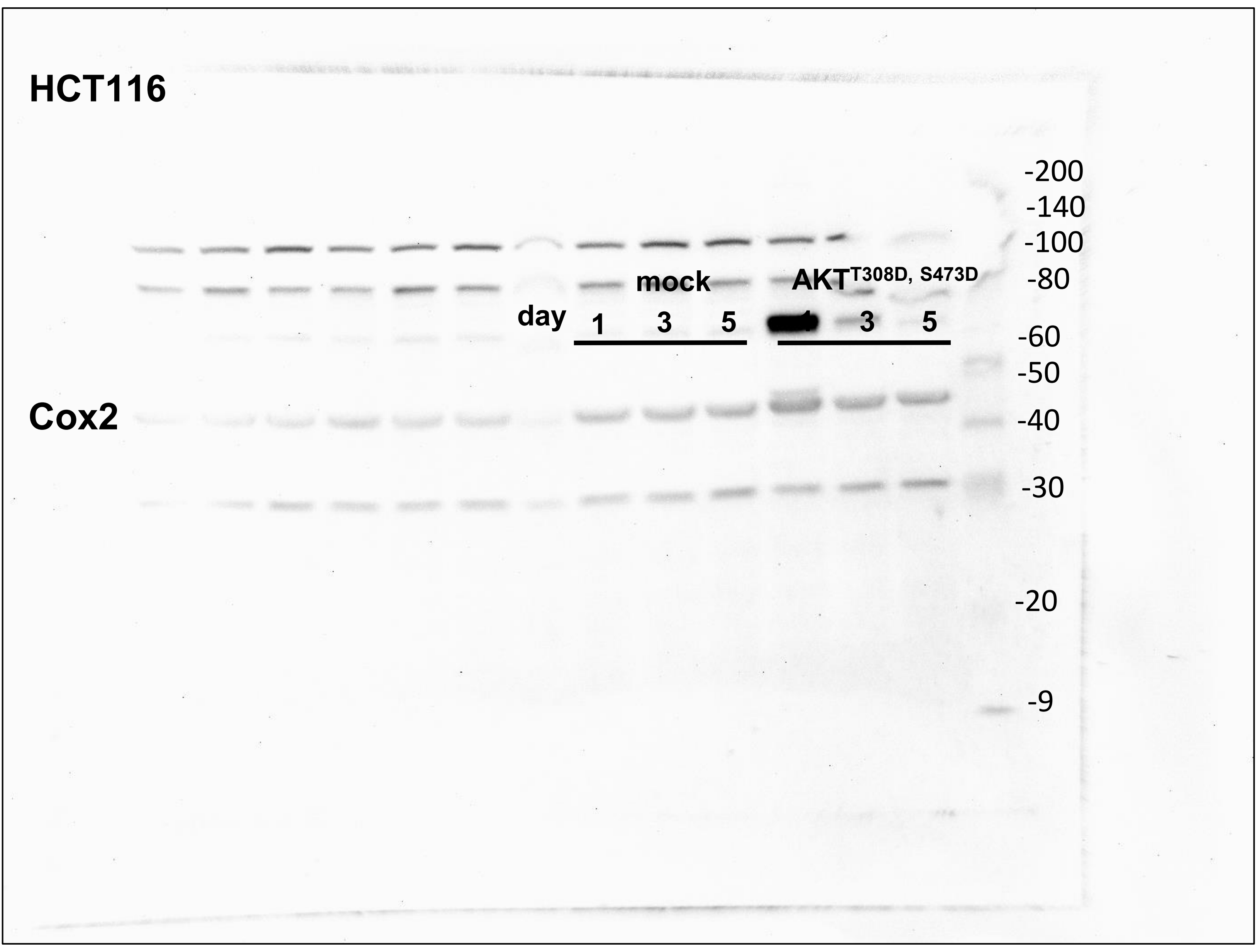

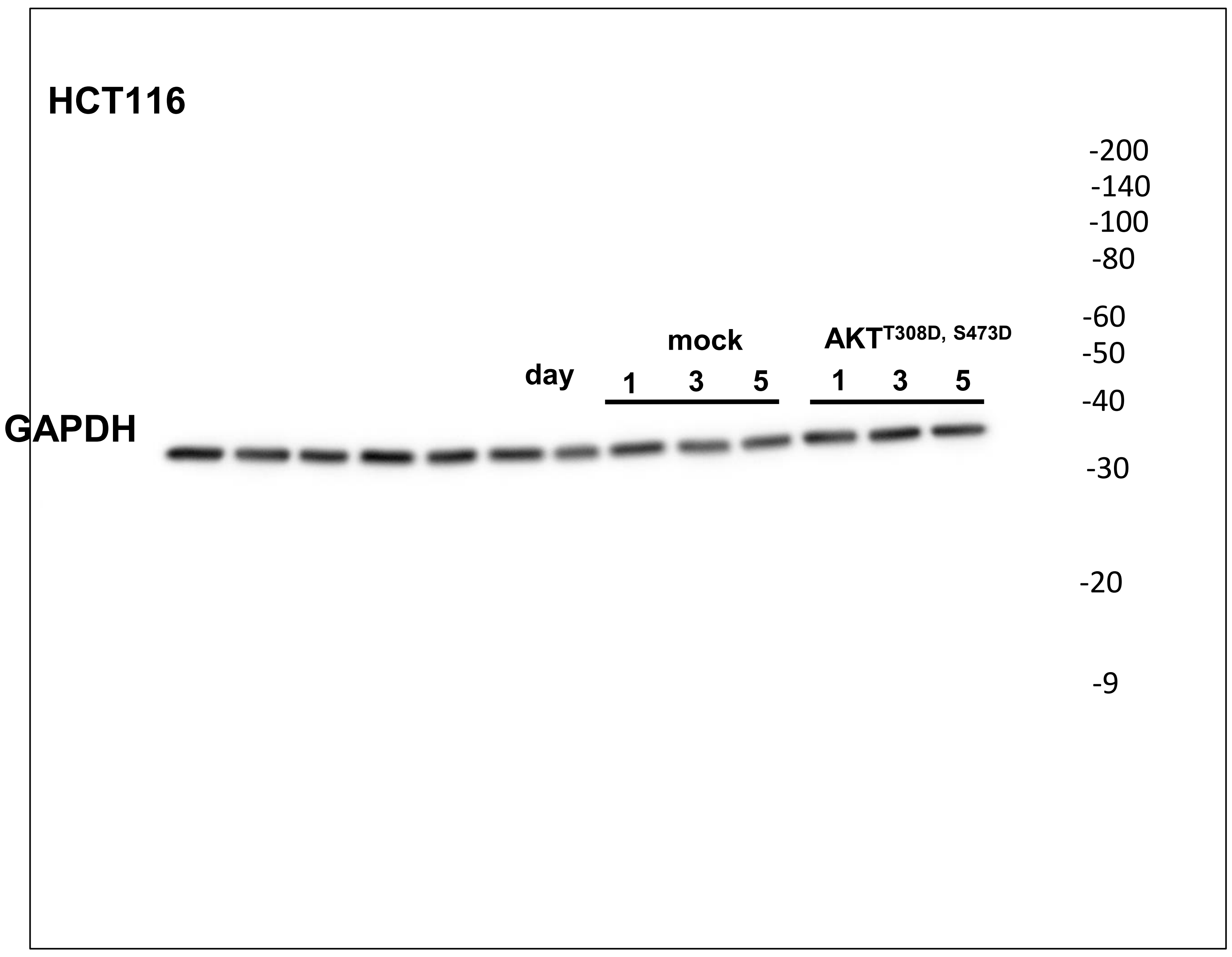

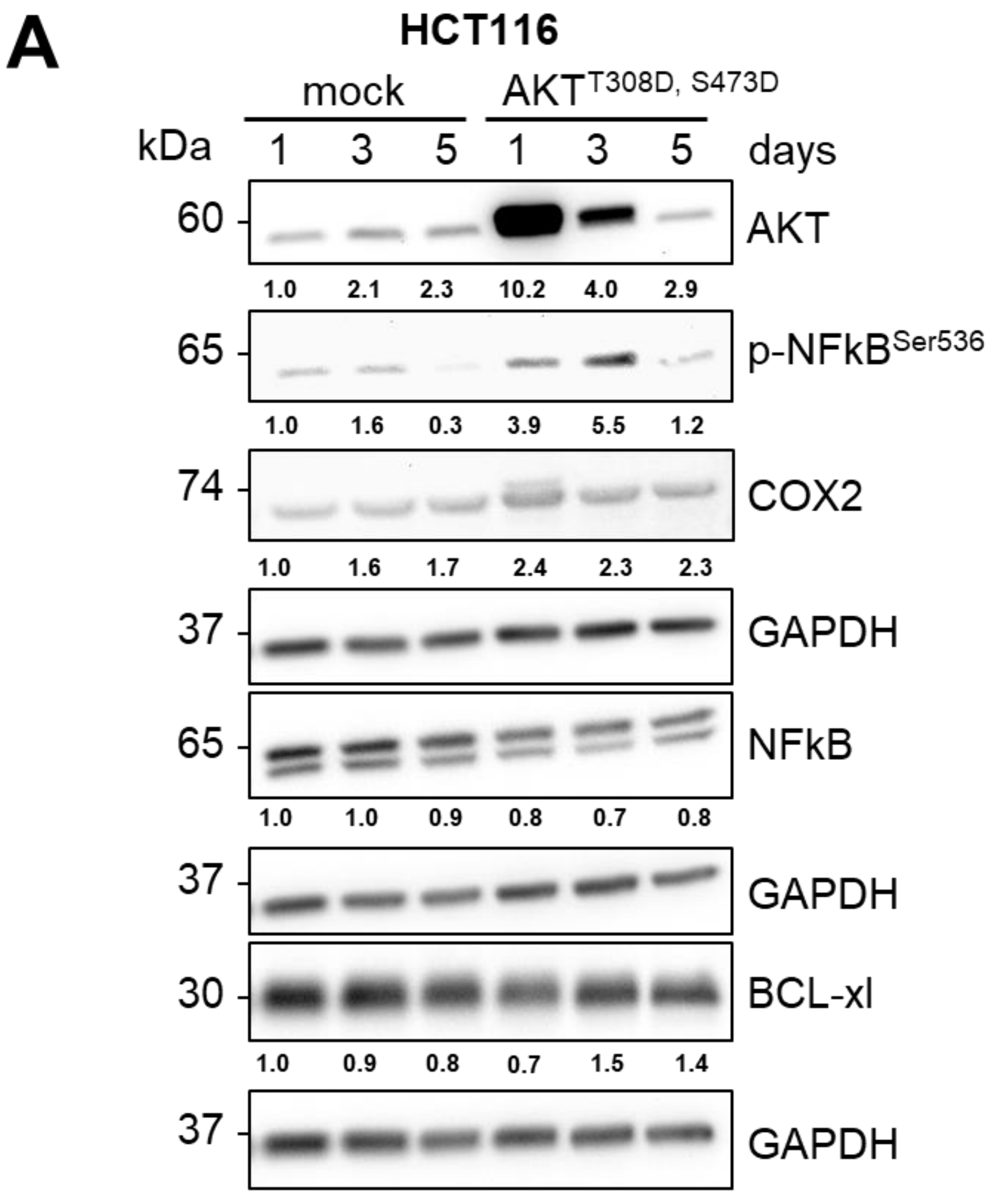

Fig 4A-western blot in manuscript 2/2

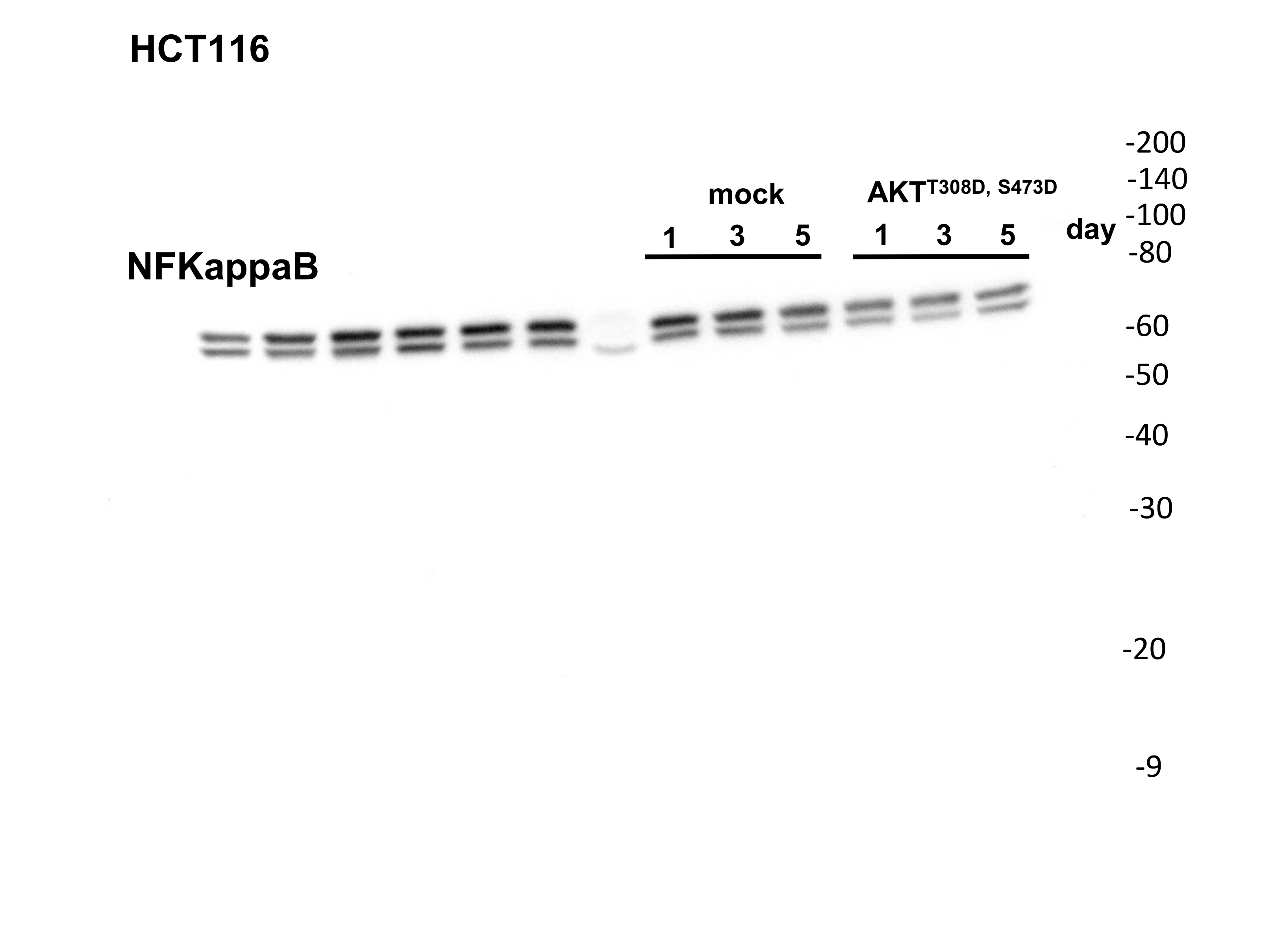

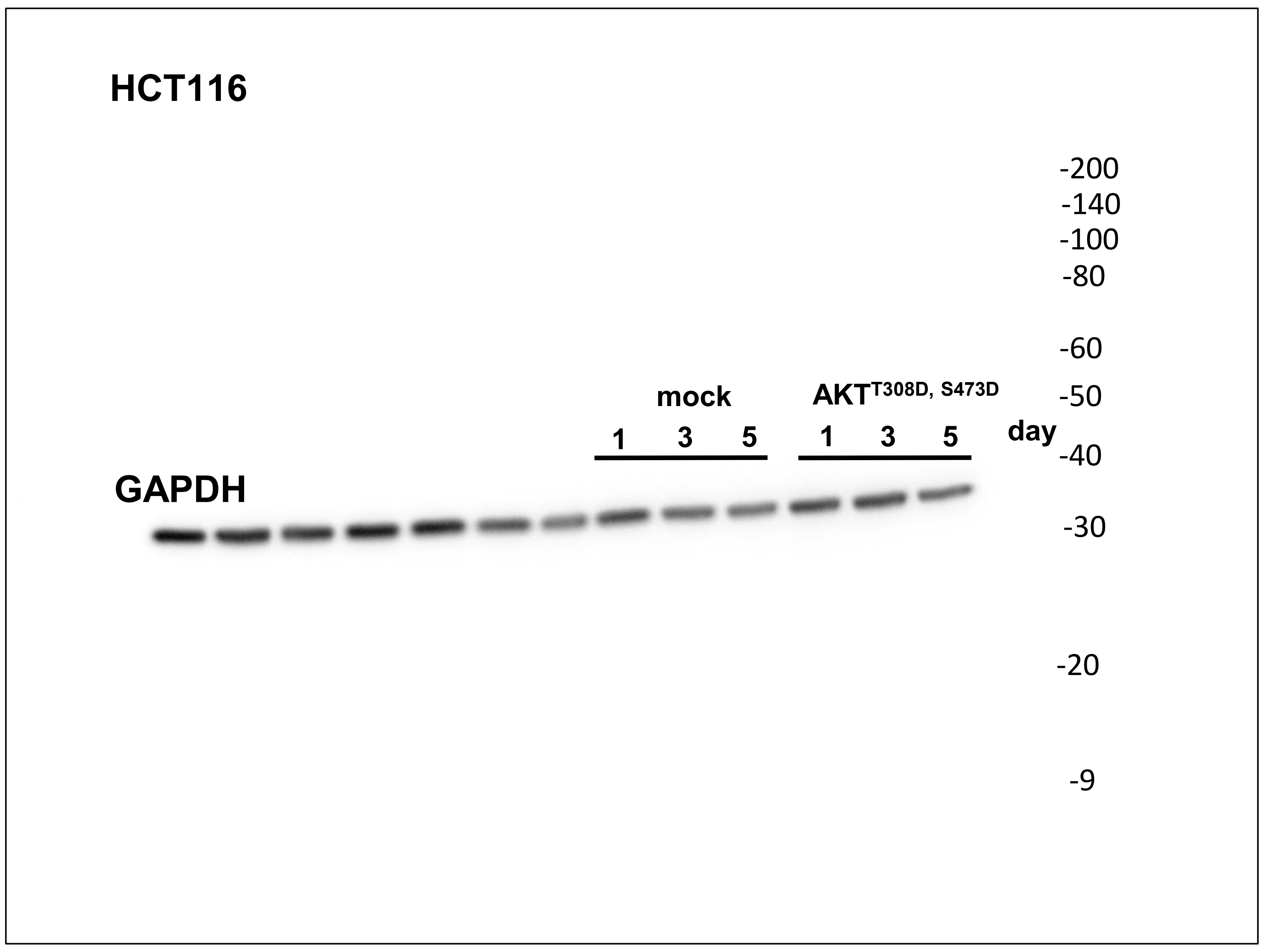

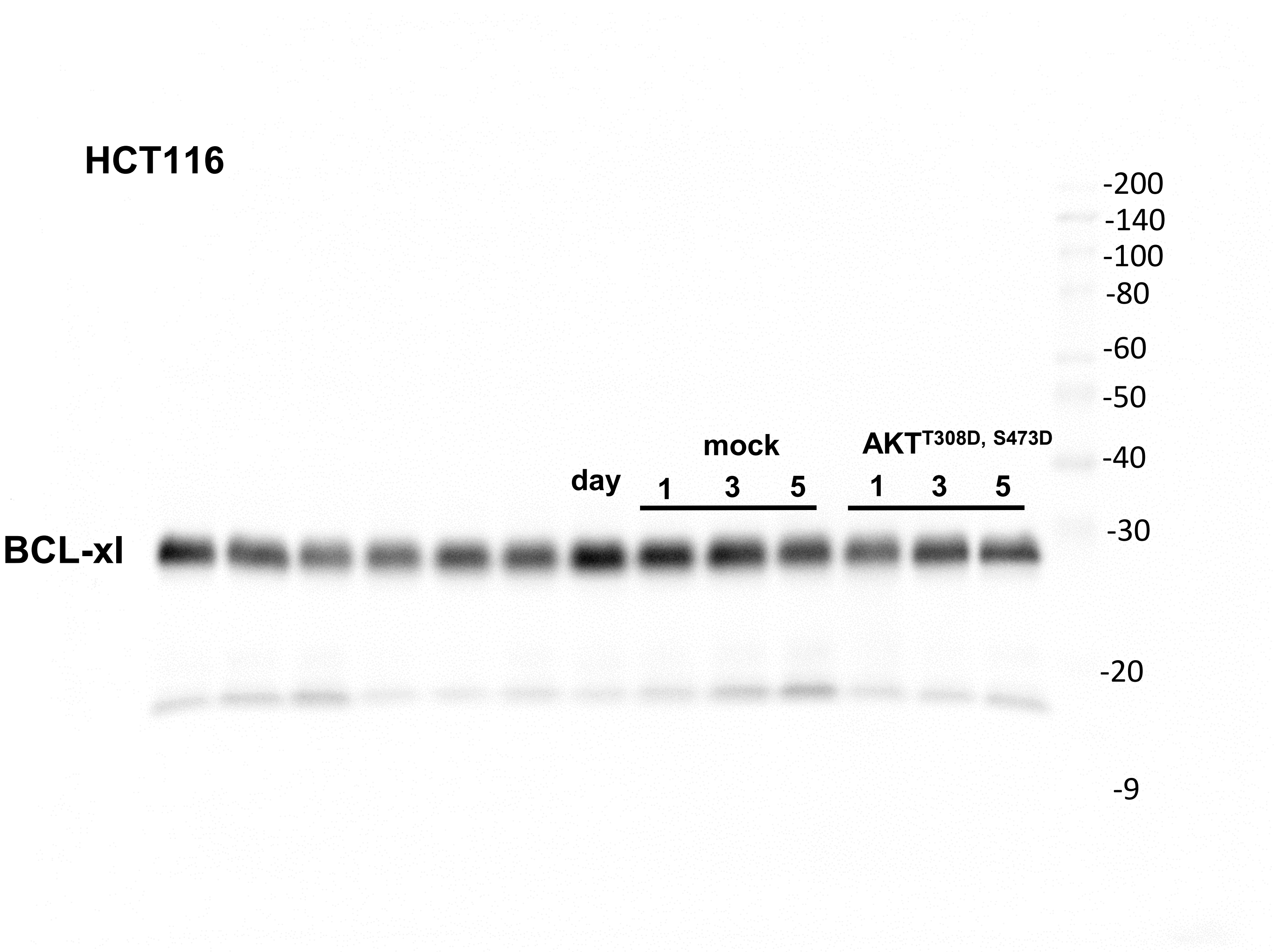

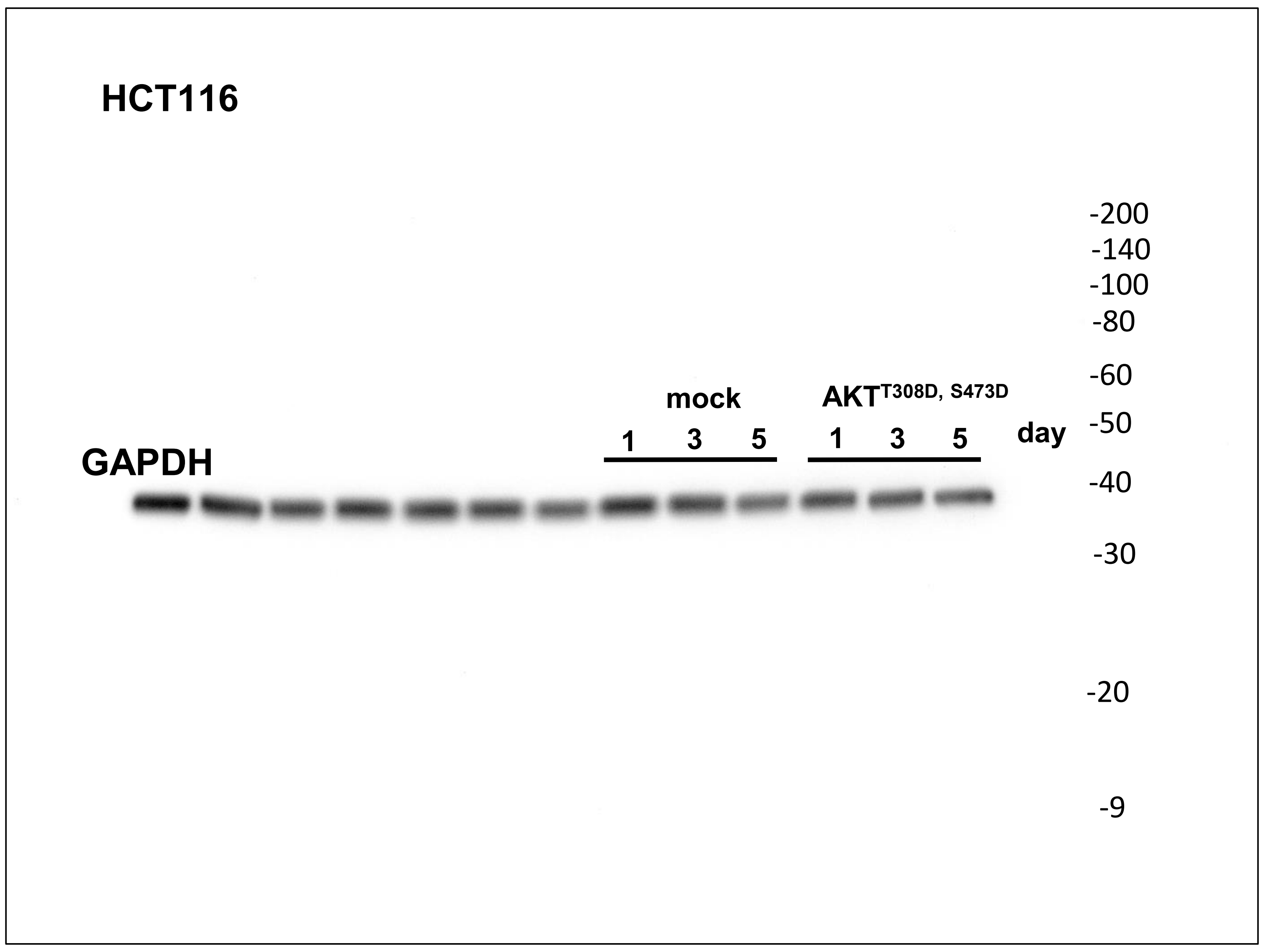

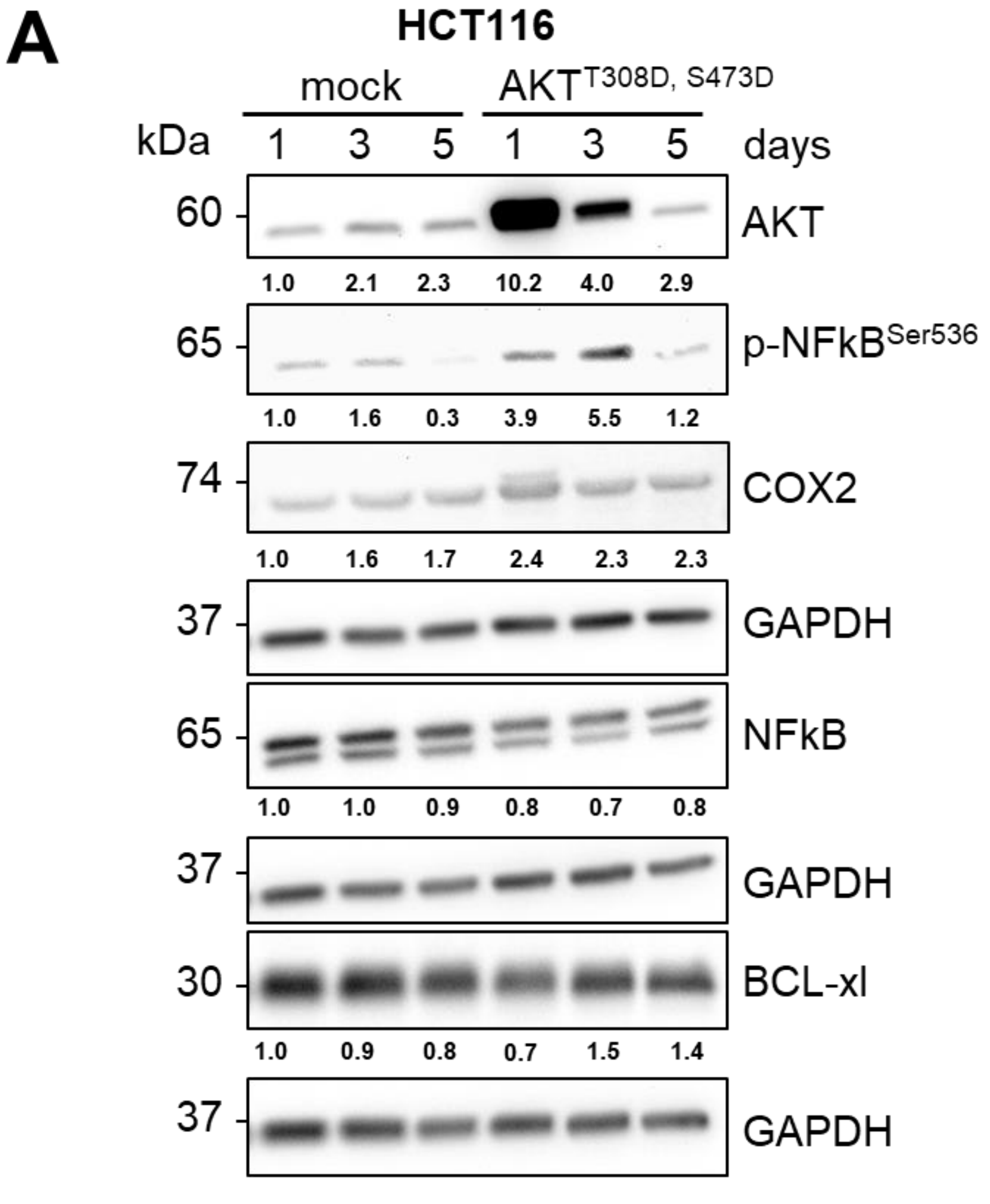

Fig 4B-western blot in manuscript 1/2

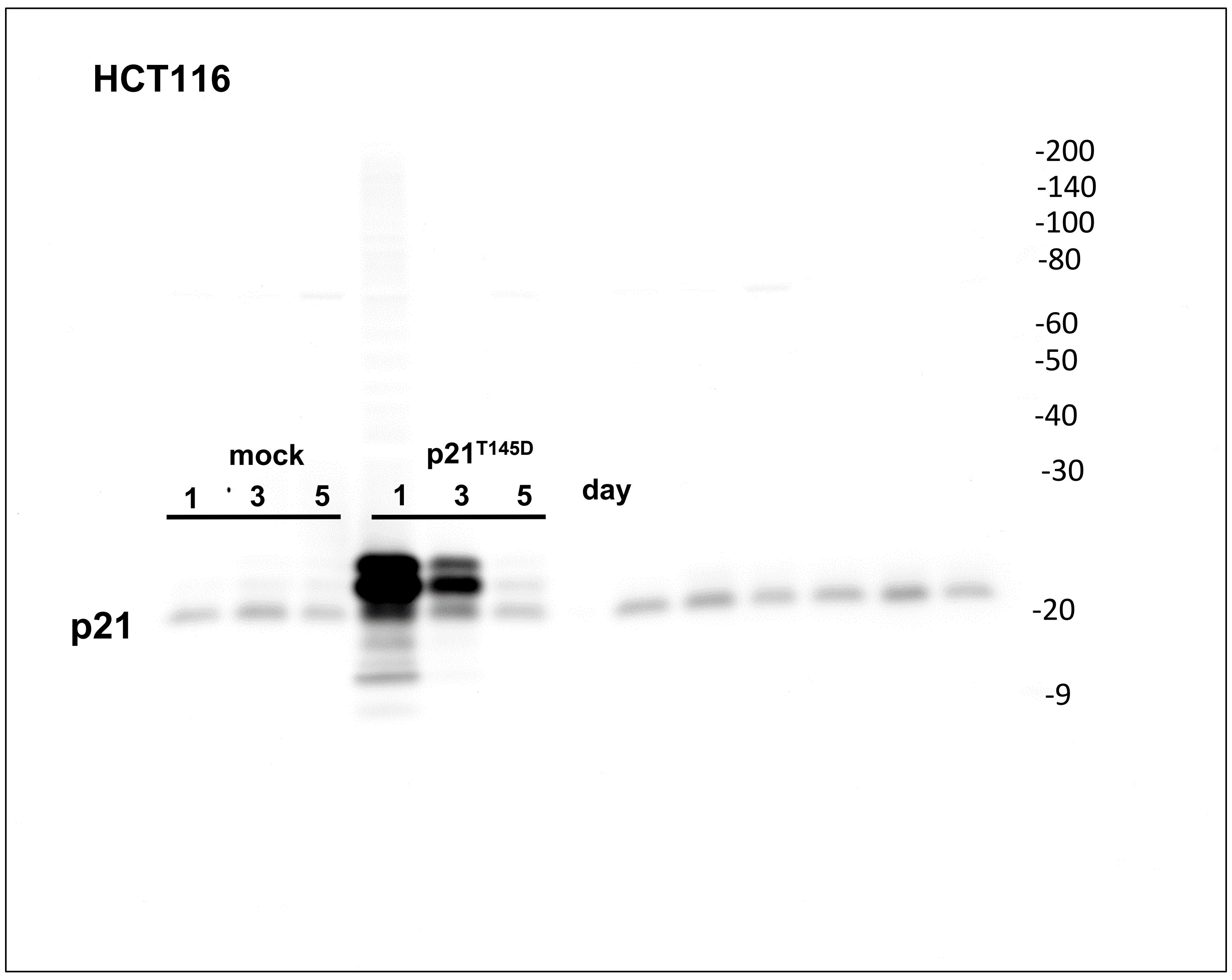

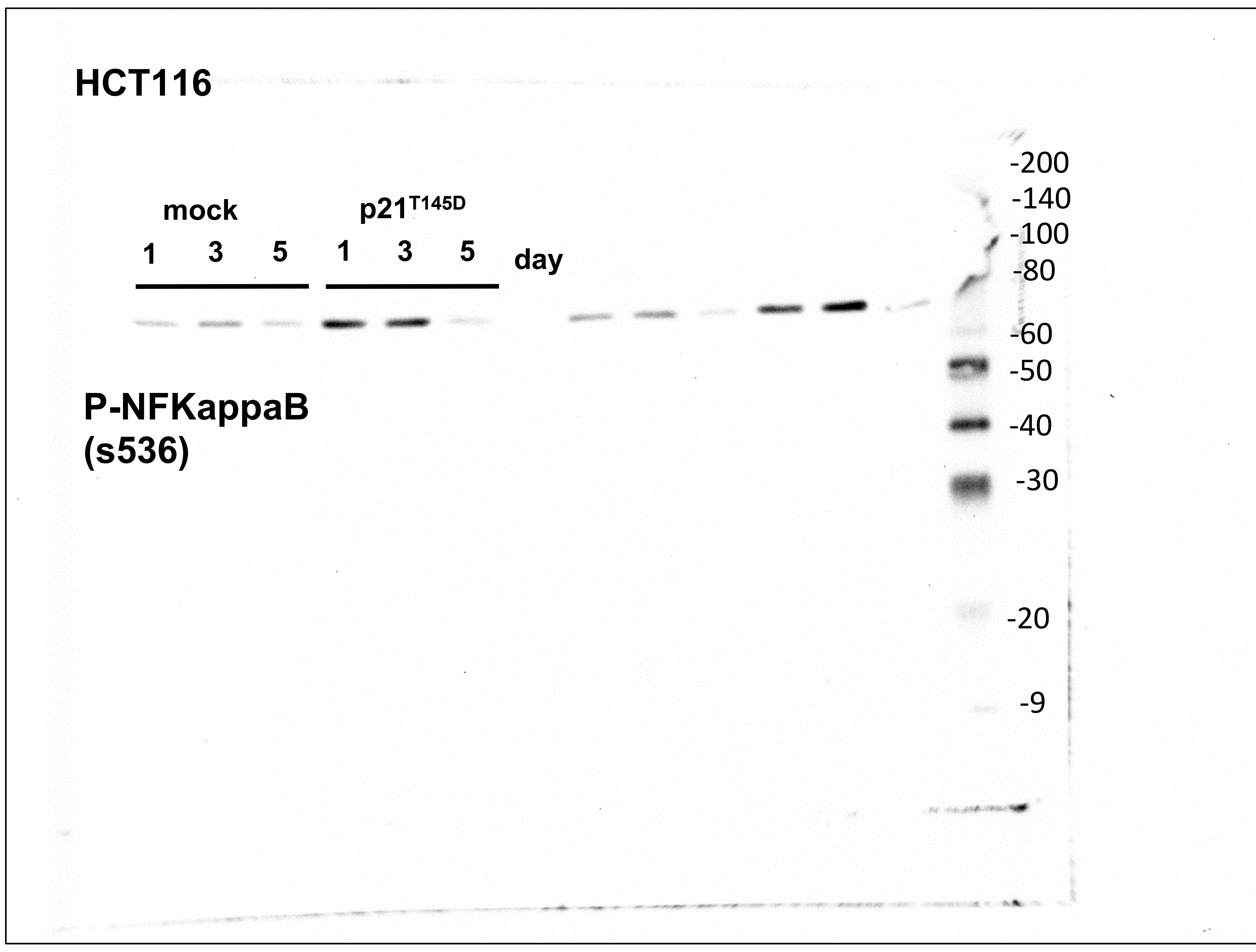

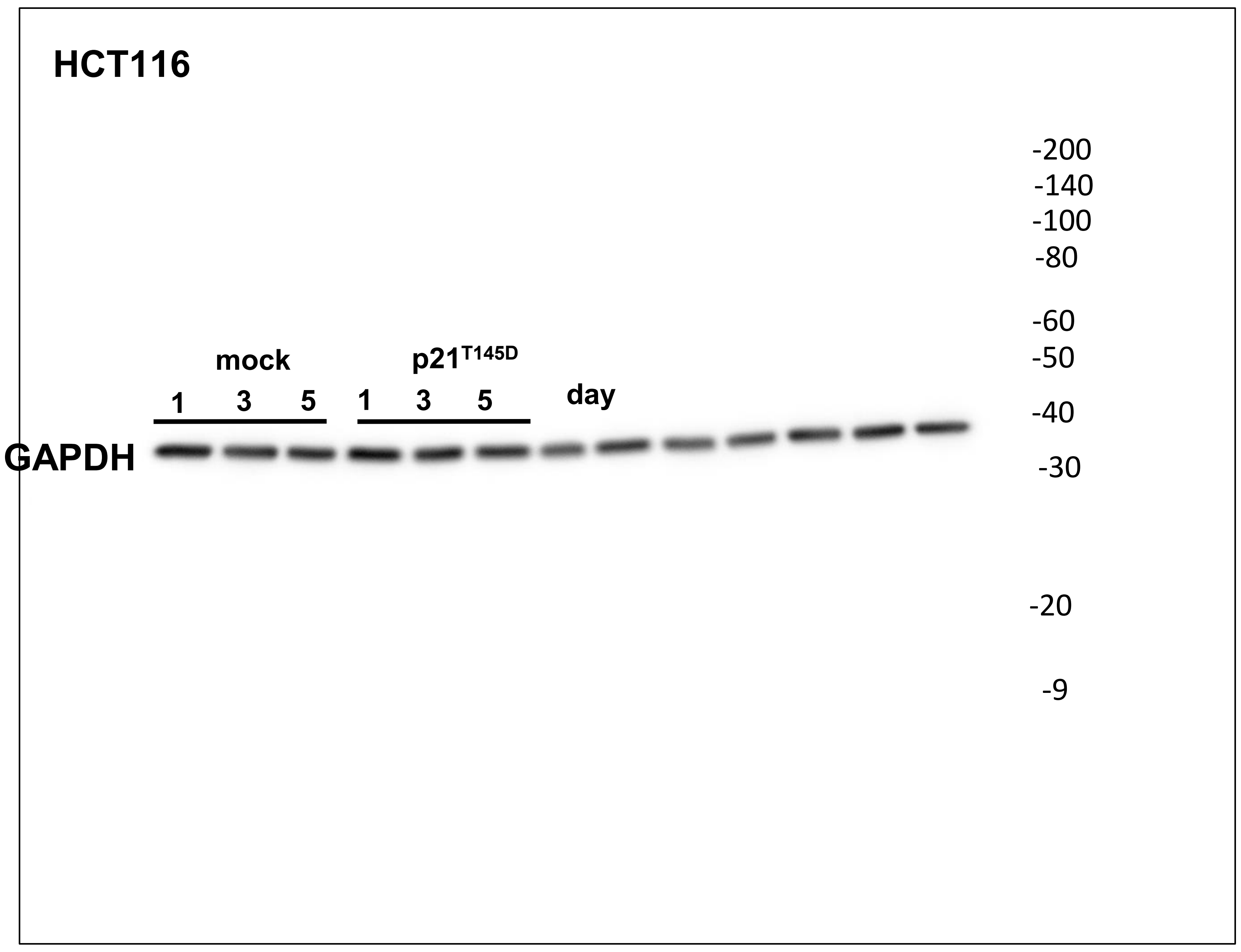

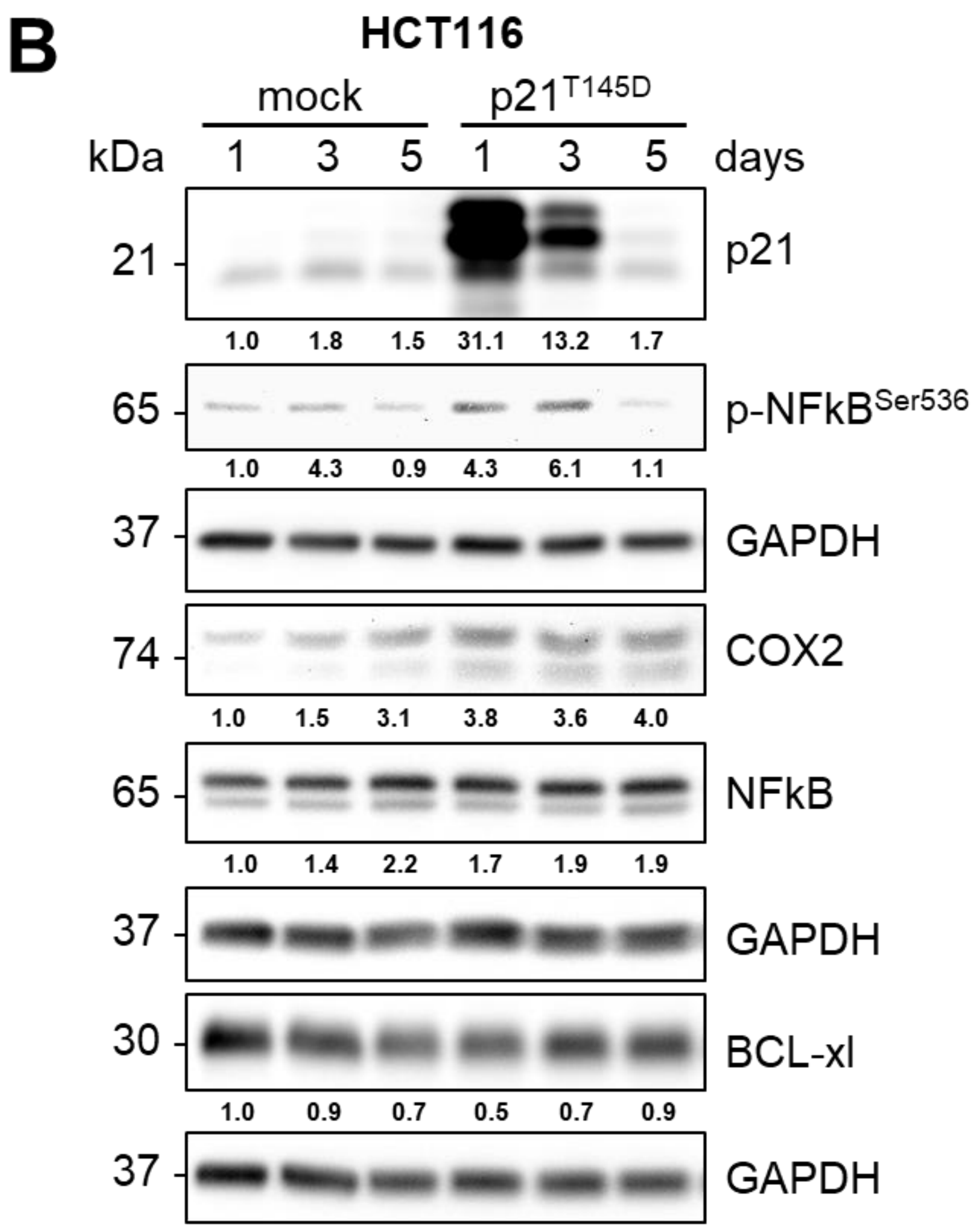

Fig 4B-western blot in manuscript 2/2

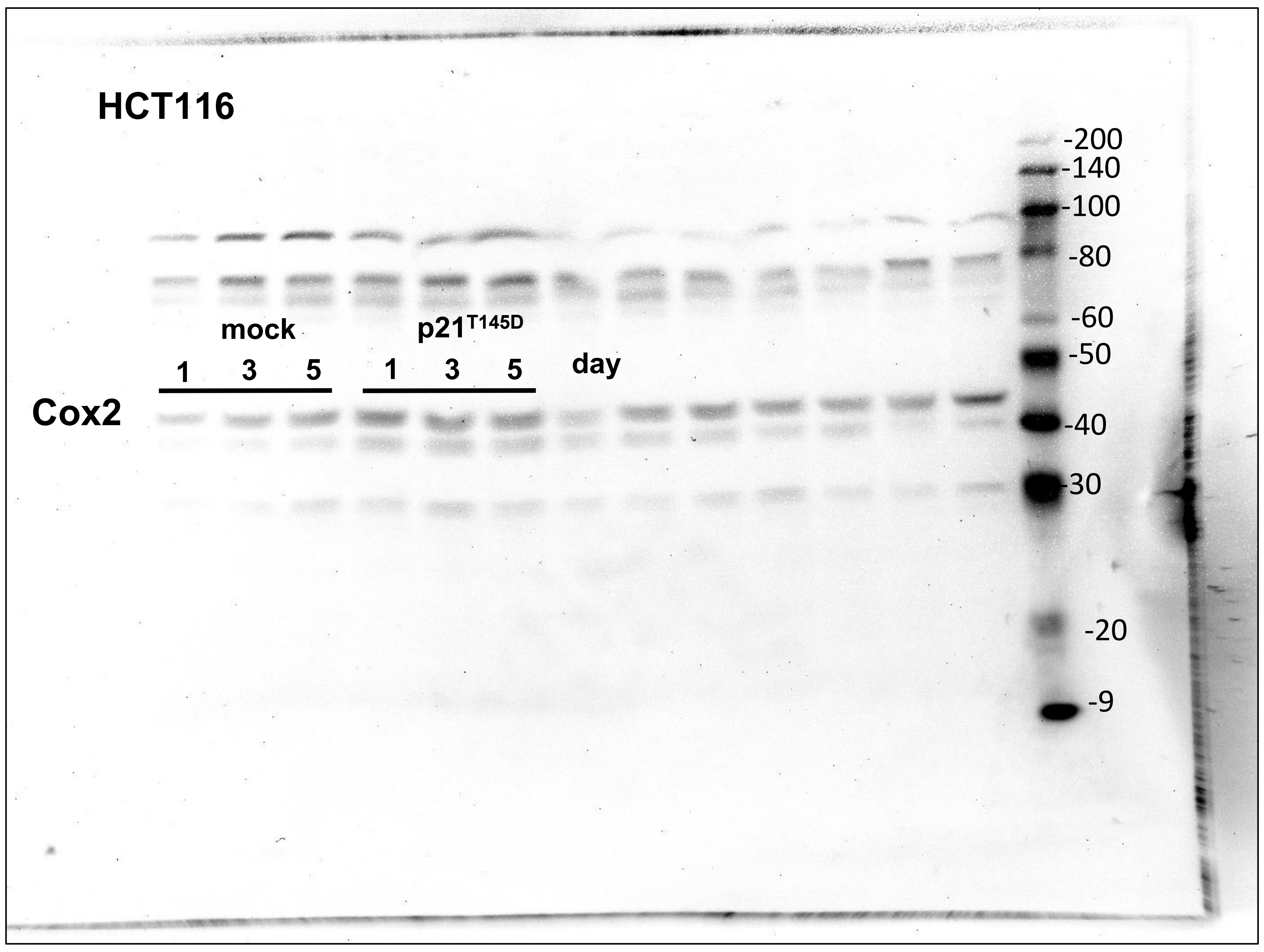

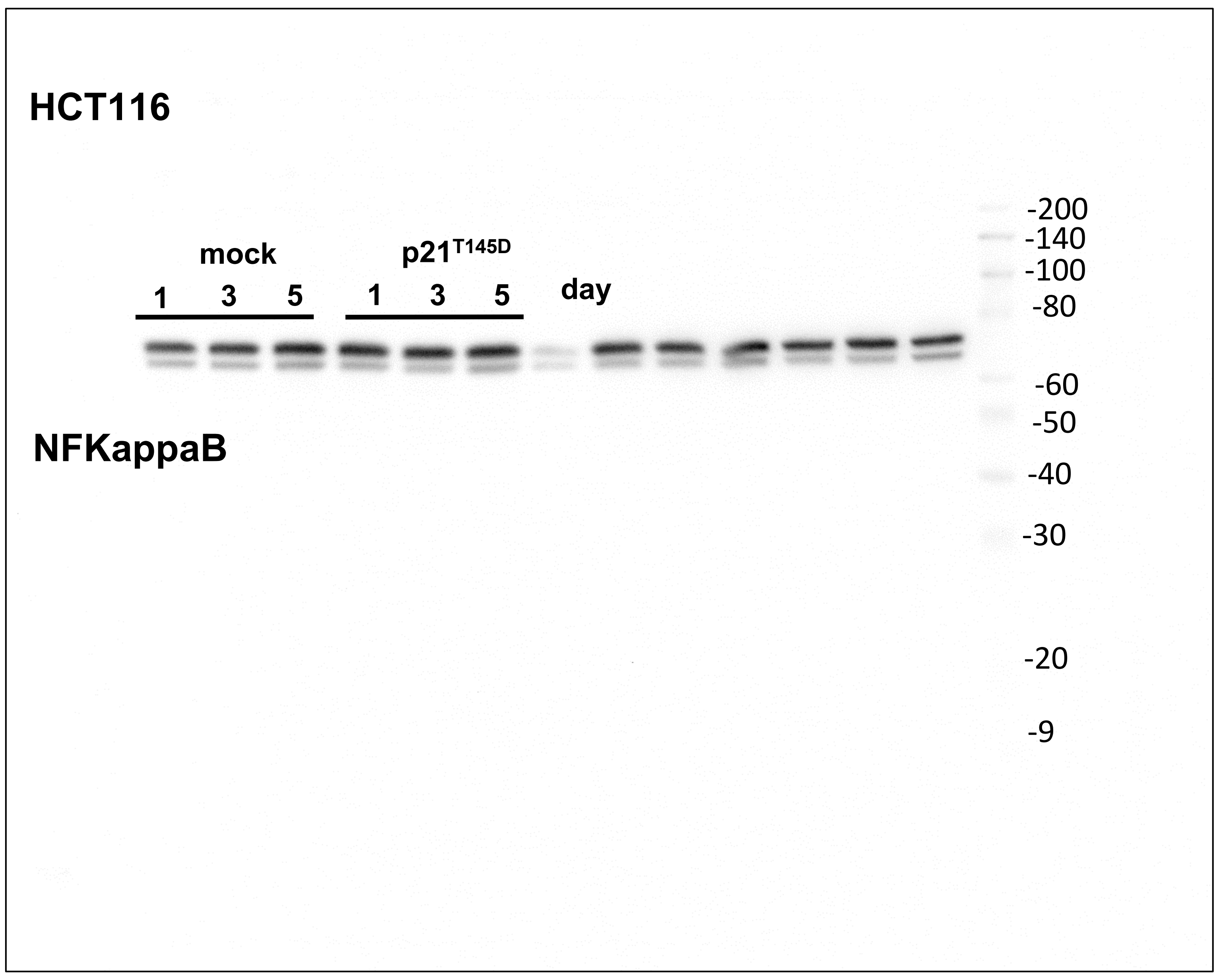

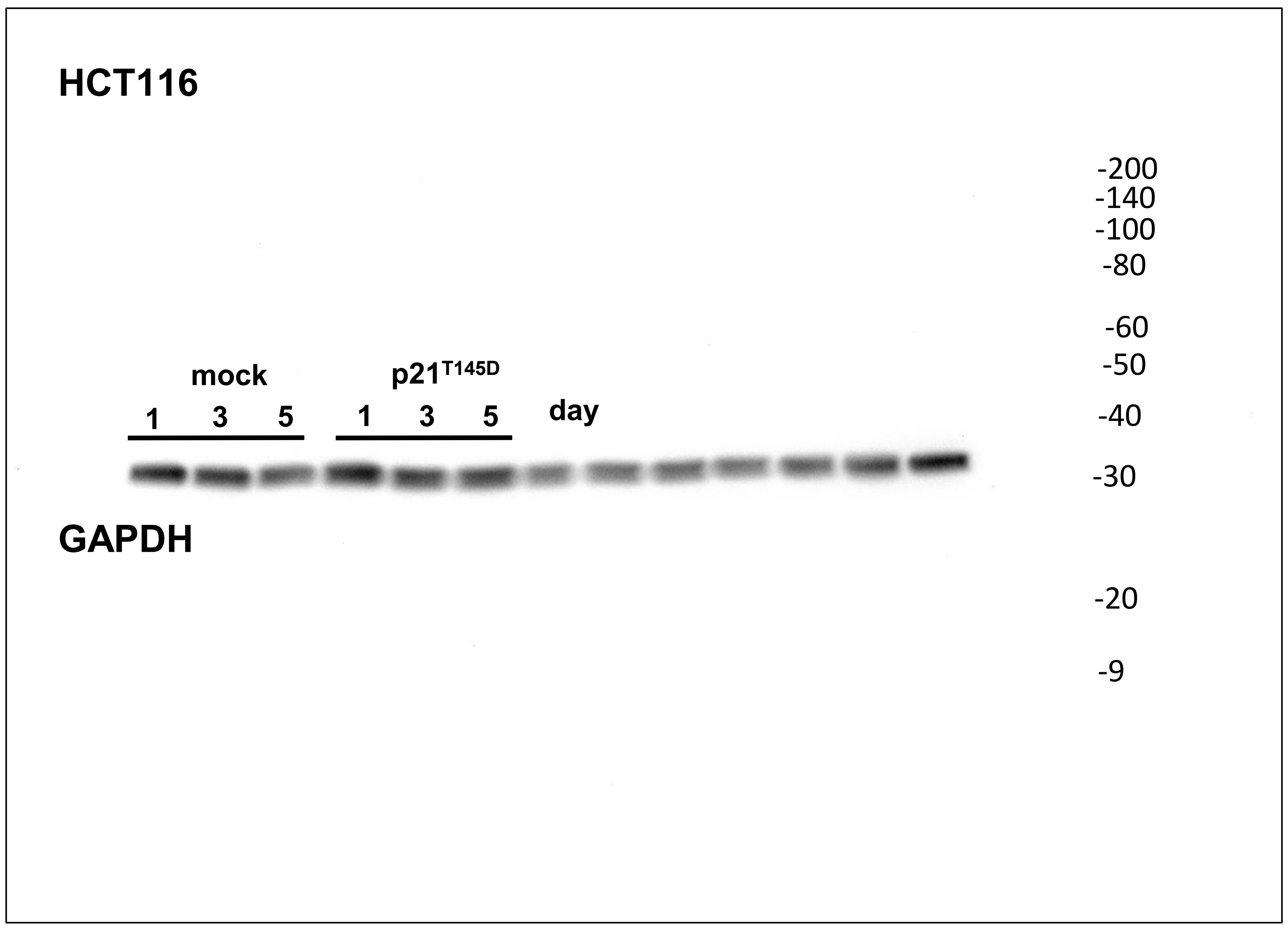

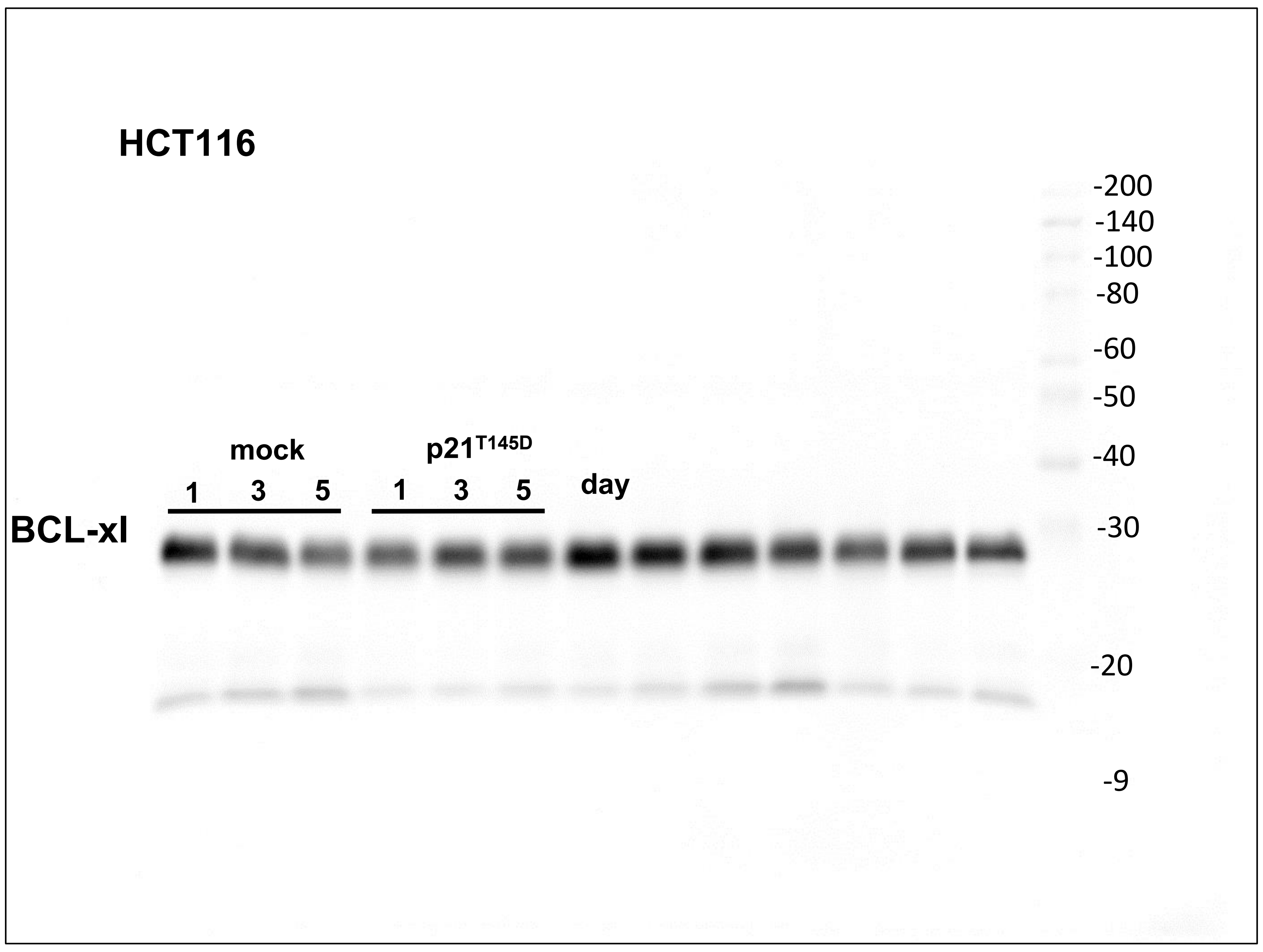

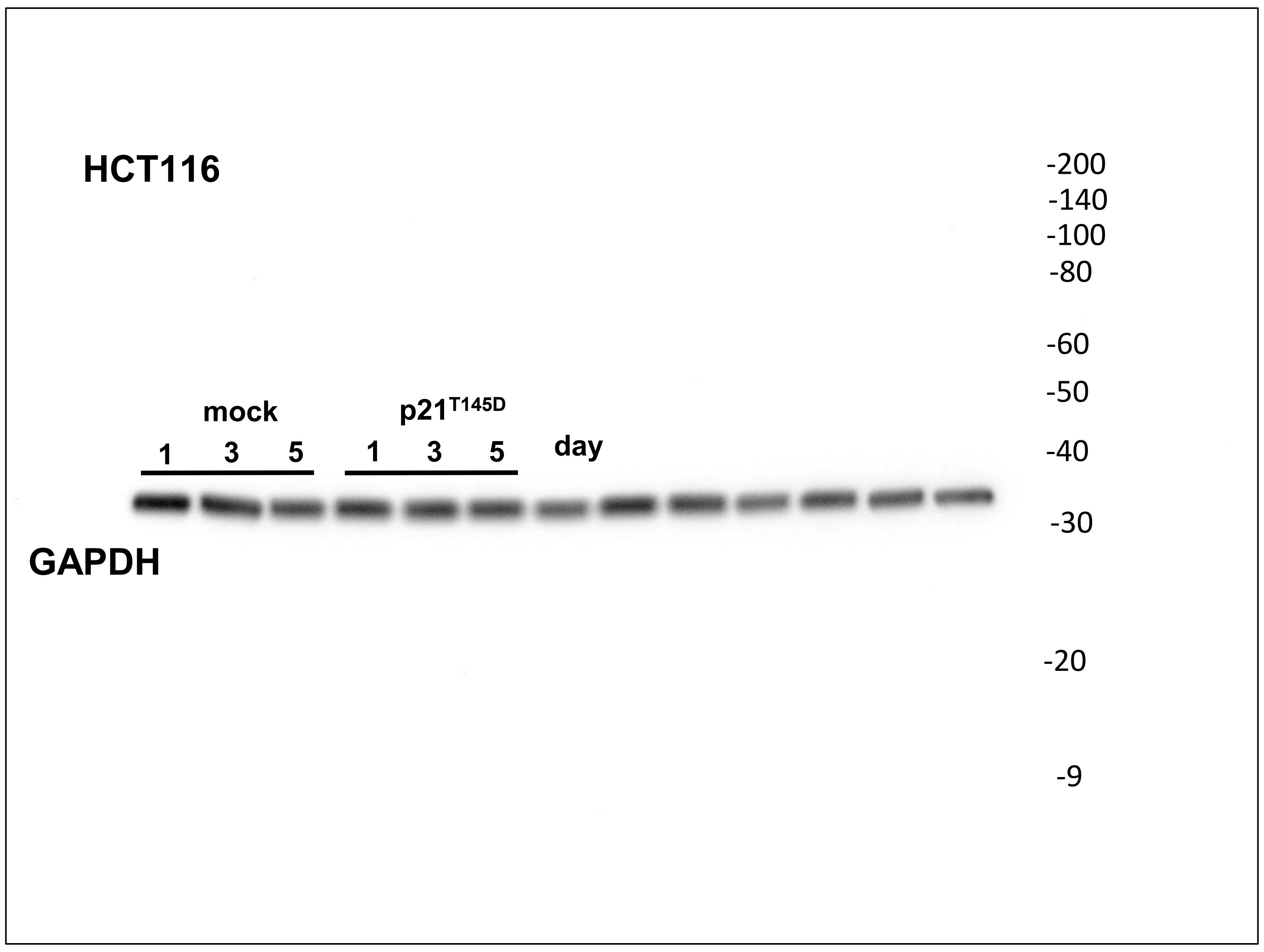

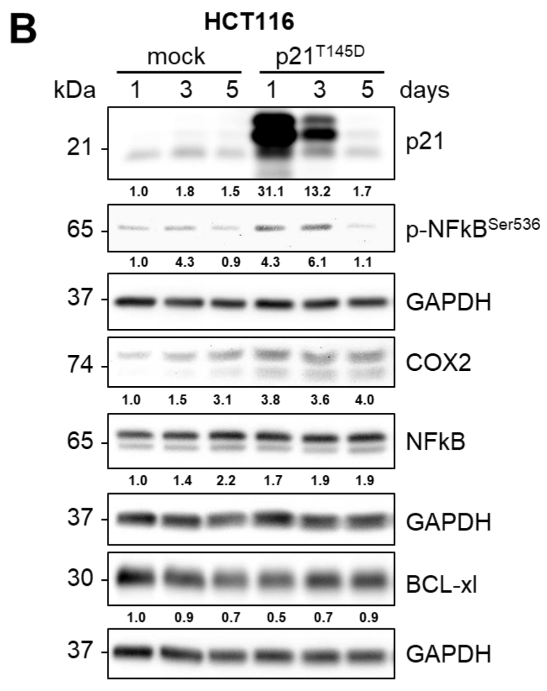

Fig 5C-western blot in manuscript

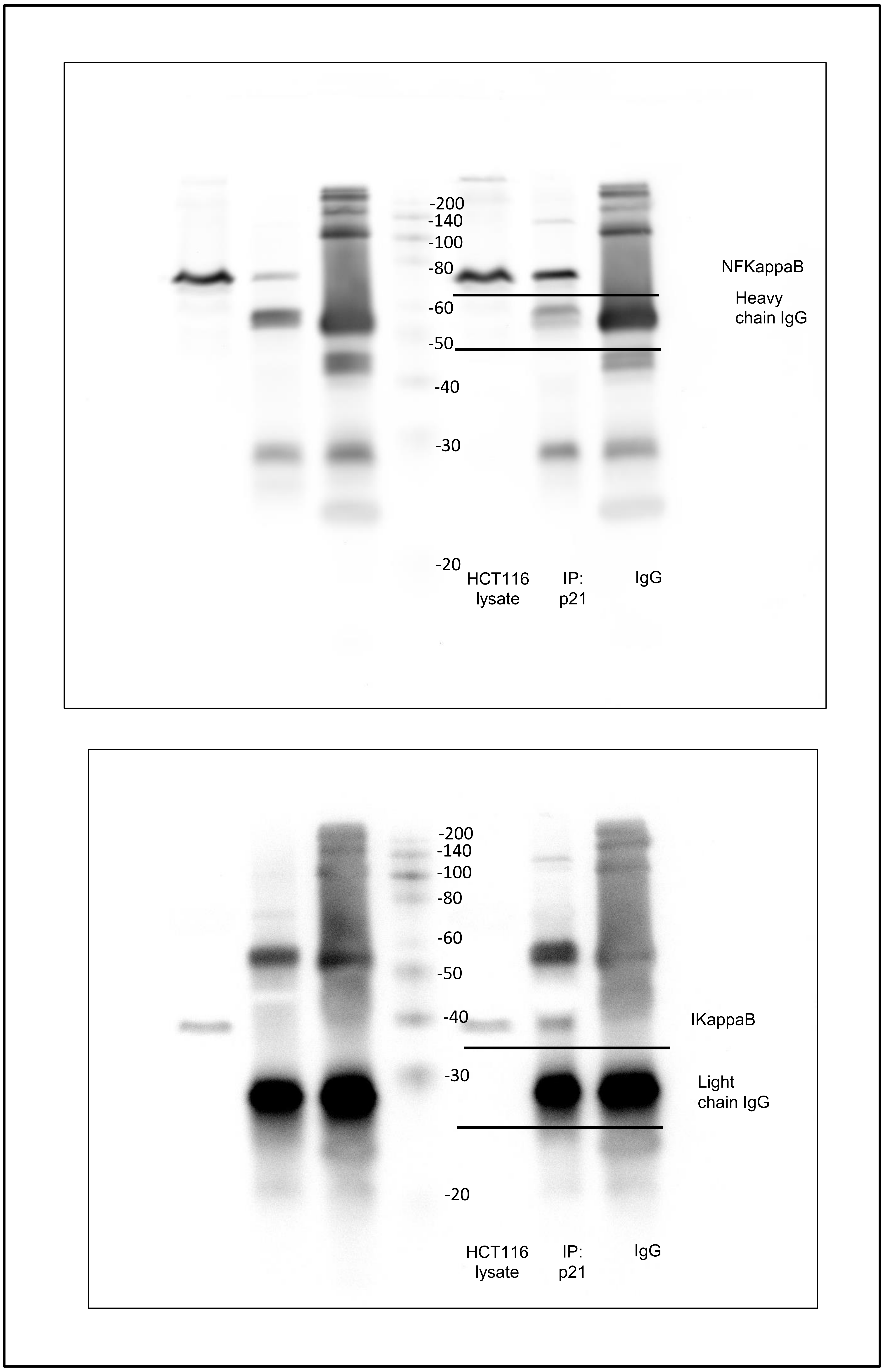

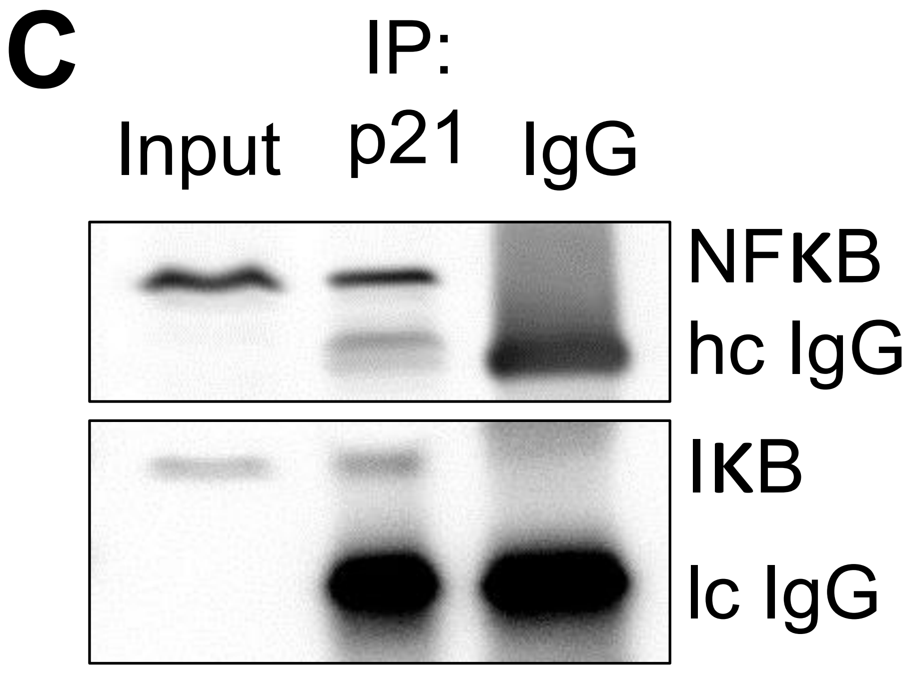

Fig 5D-western blot in manuscript

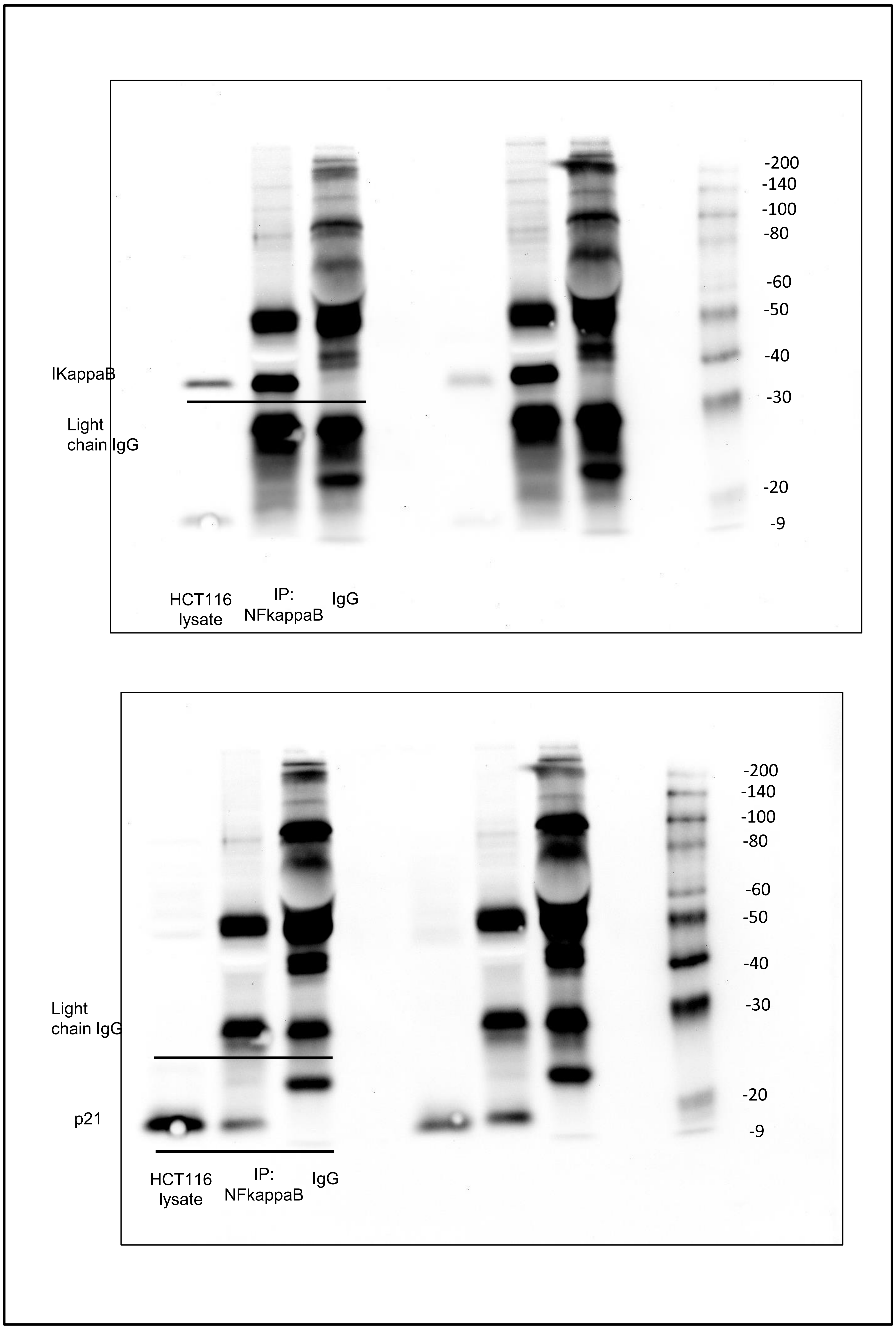

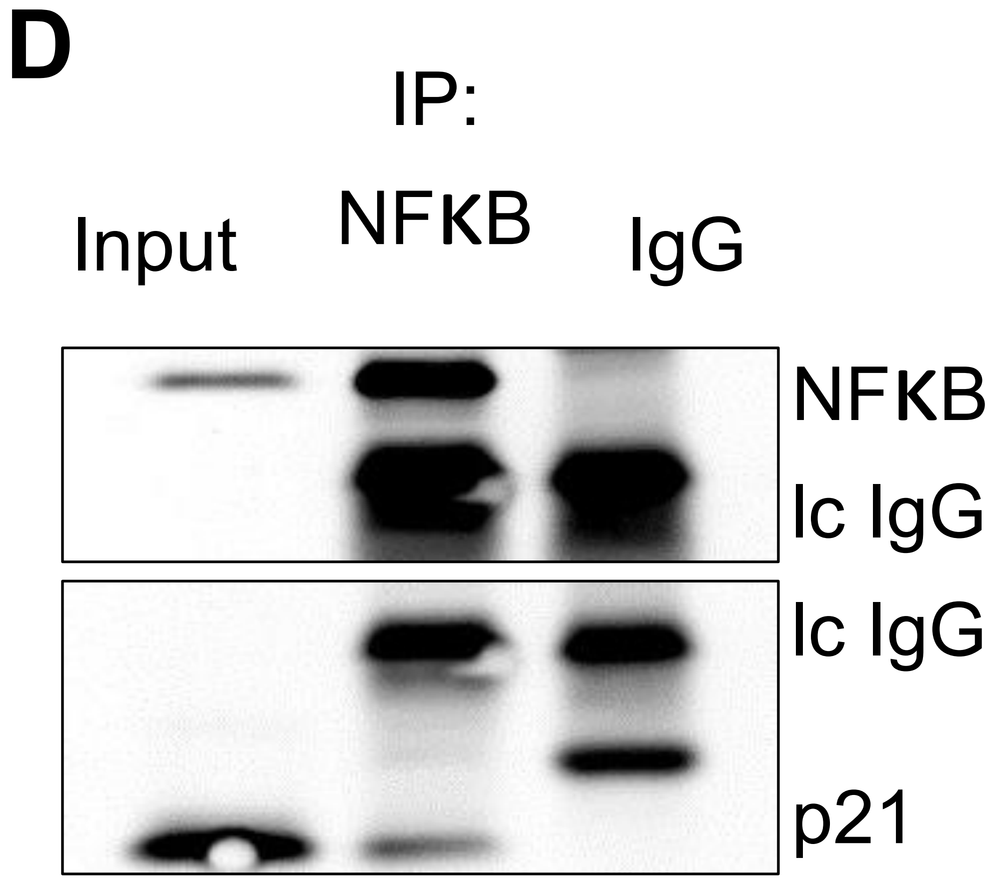

Fig 5E-western blot in manuscript

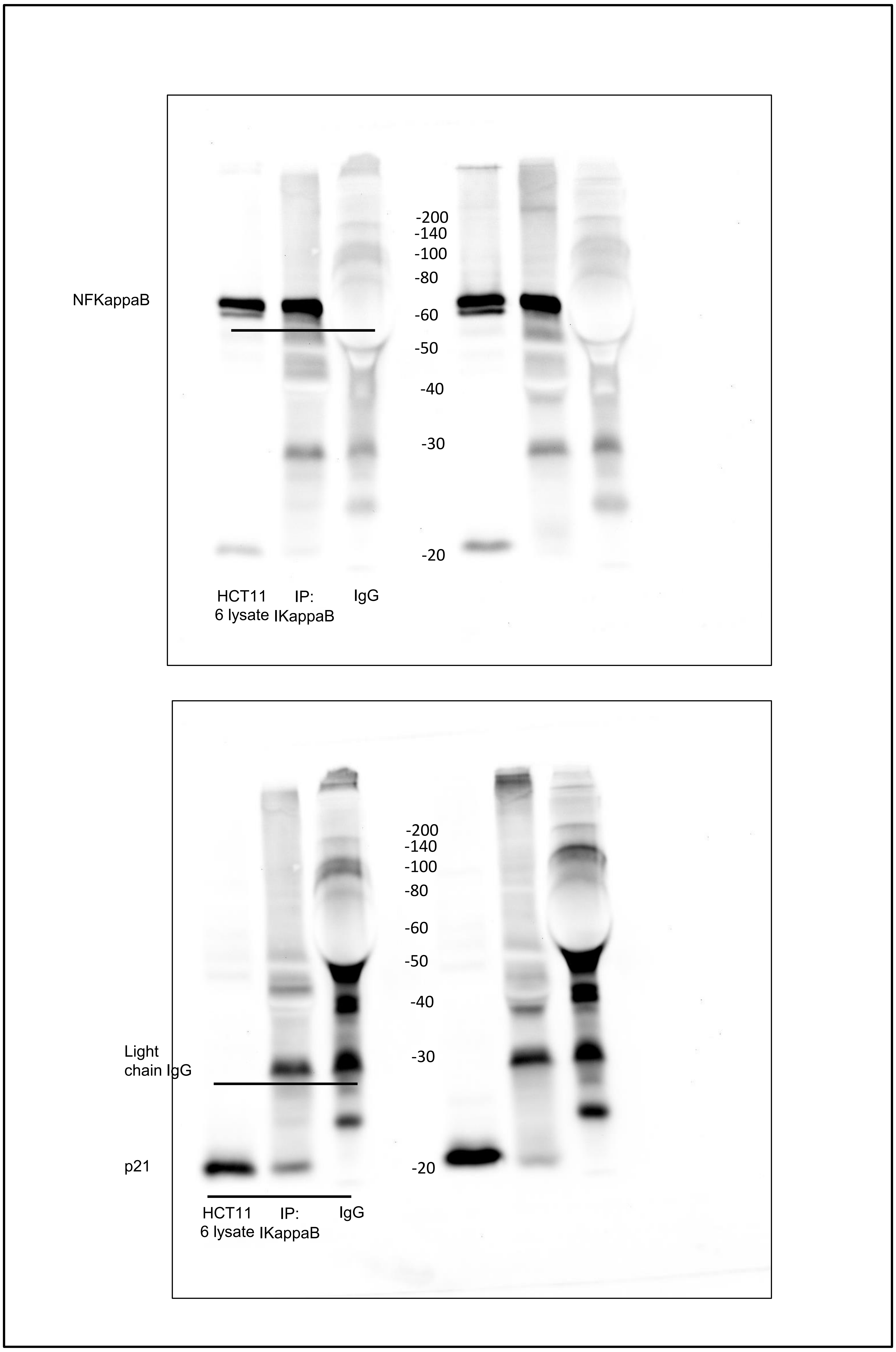

E

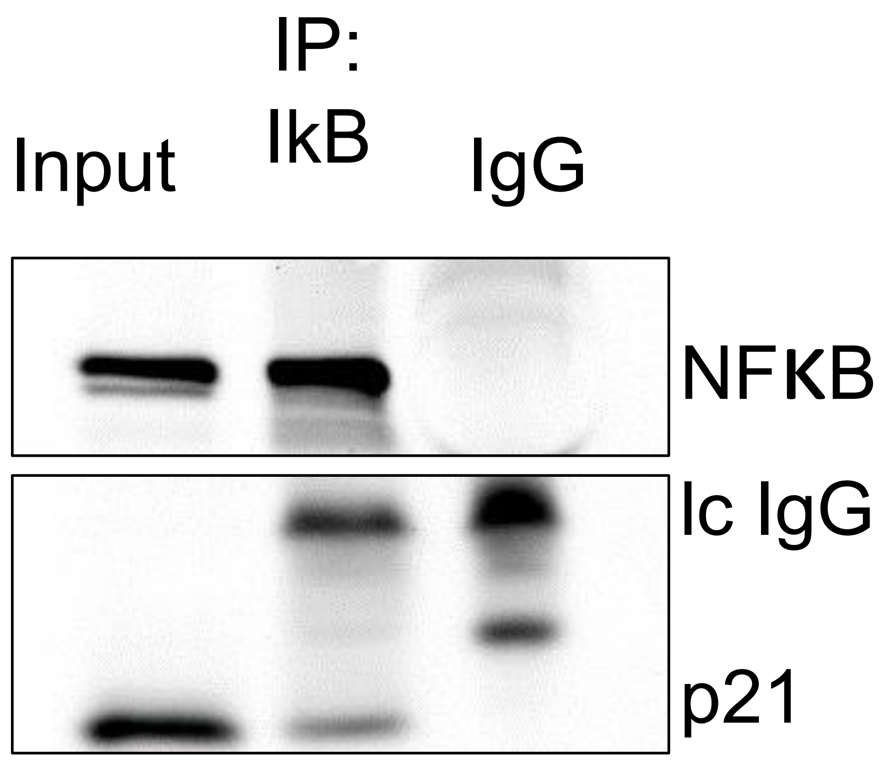

Fig 5H-western blot in manuscript

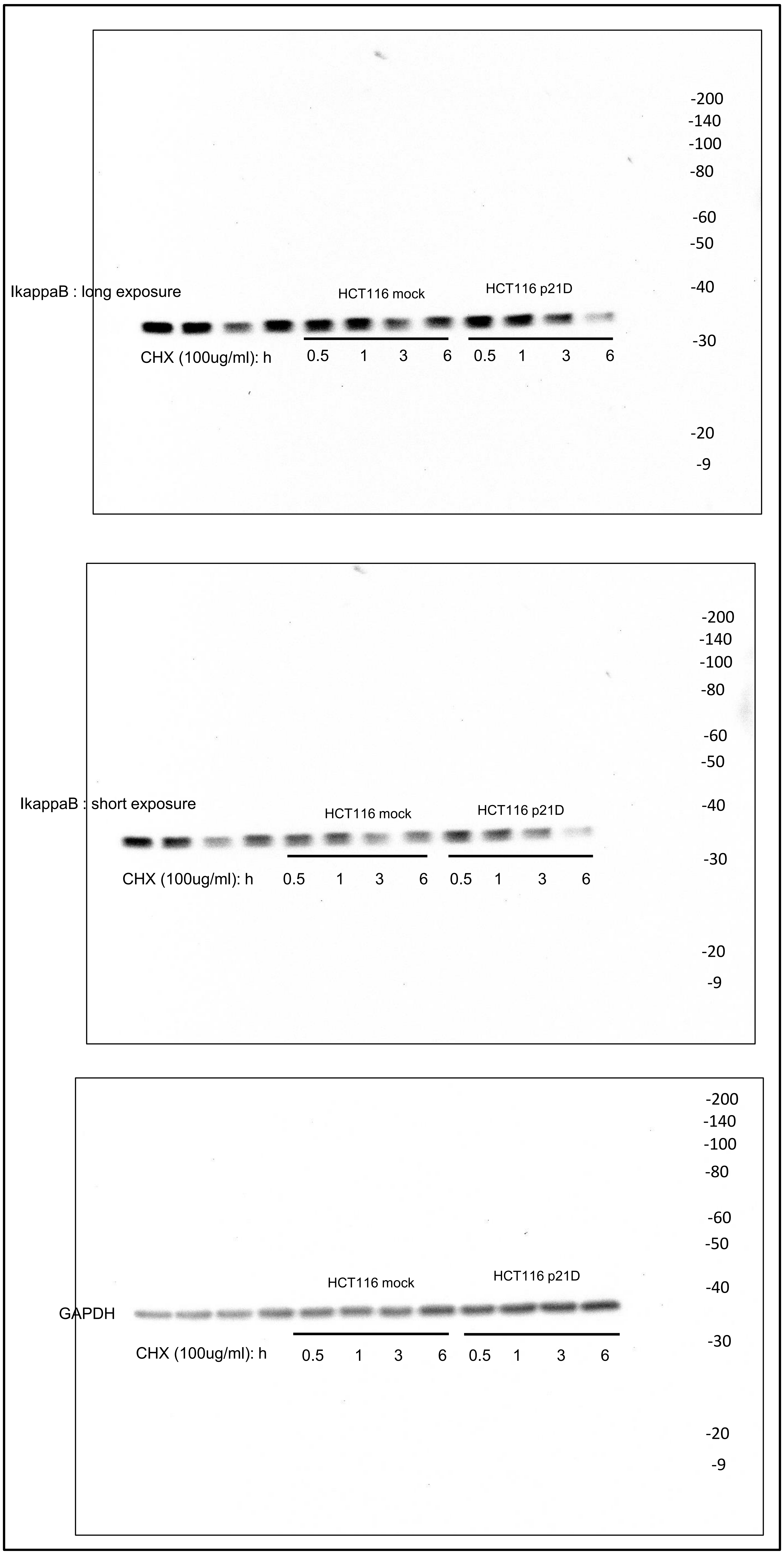

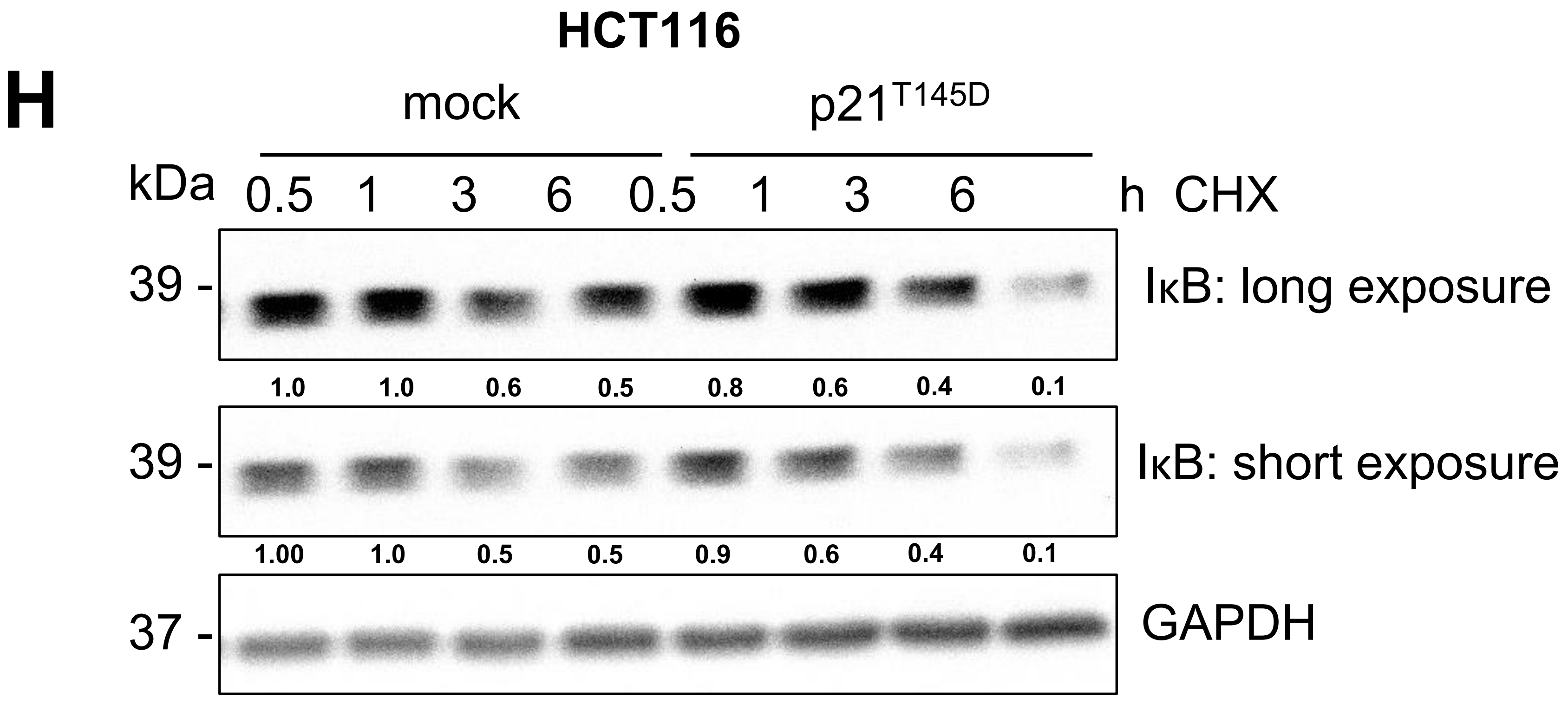

Fig 5I-western blot in manuscript

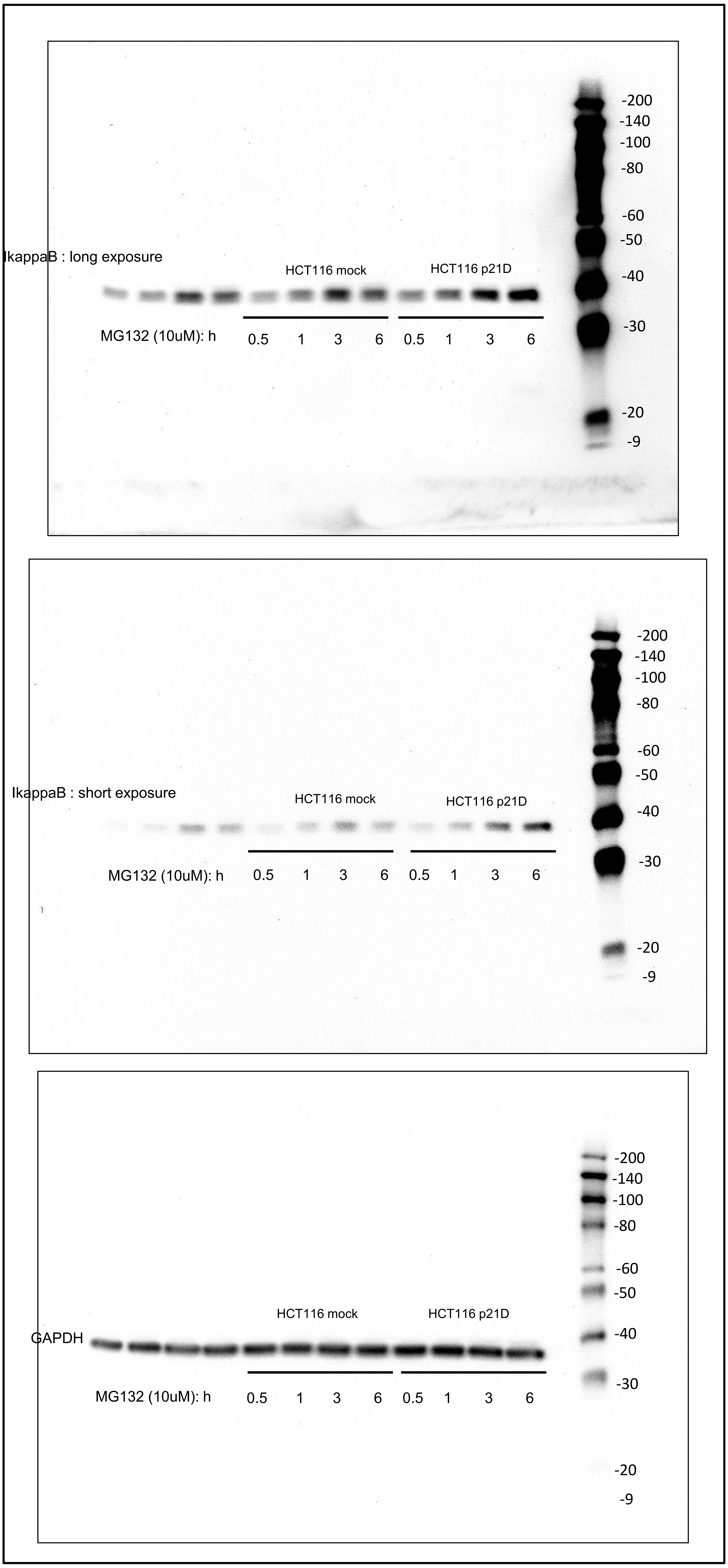

I

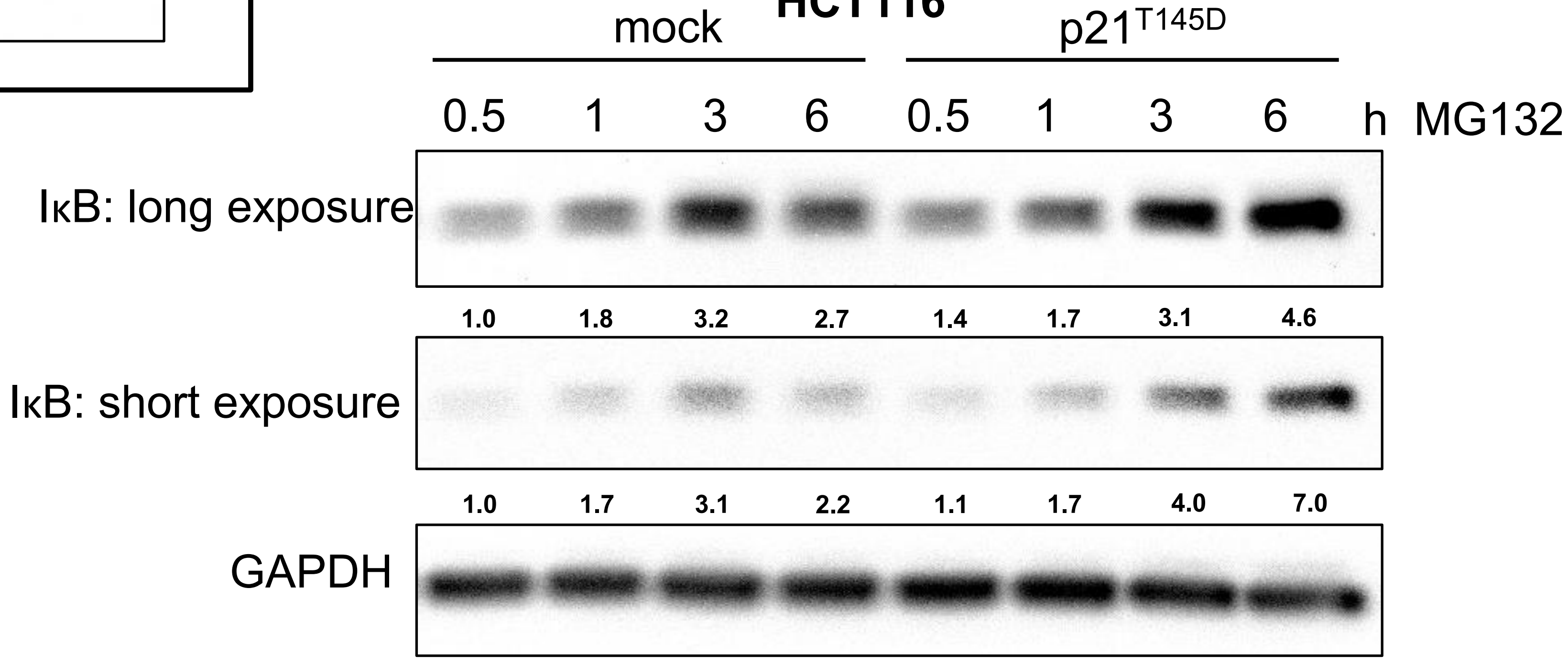

Fig S1-western blot in manuscript

selected bands

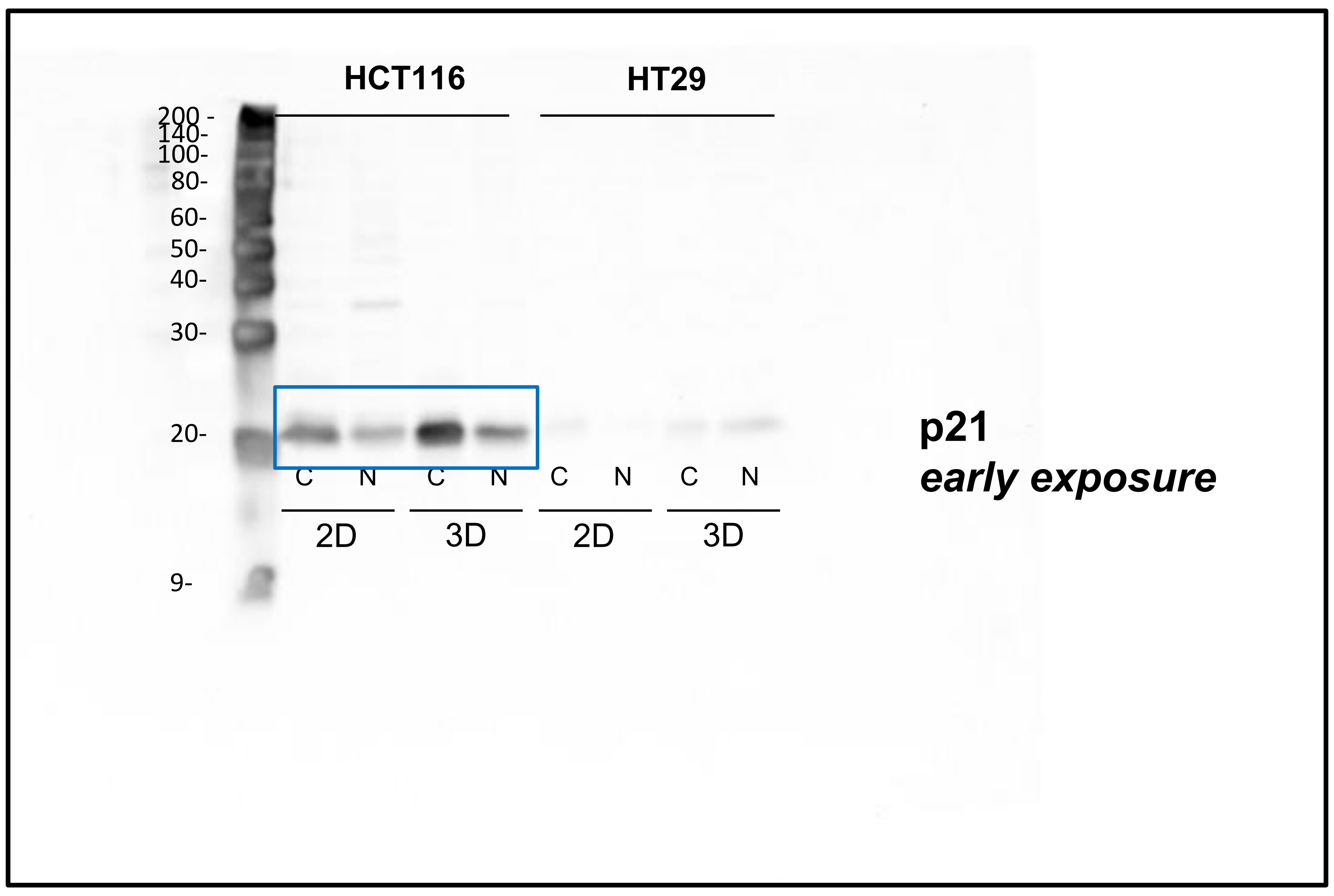

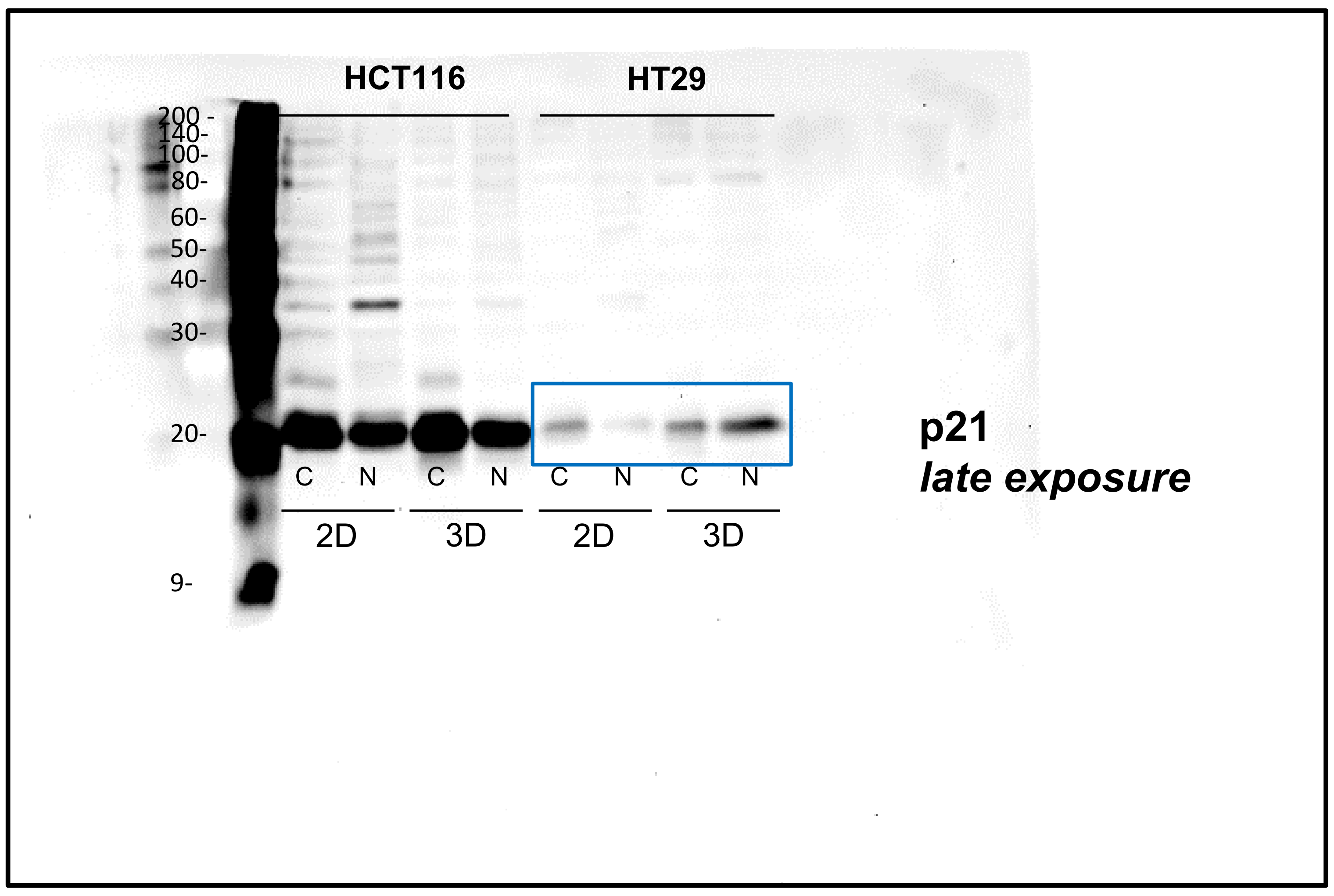

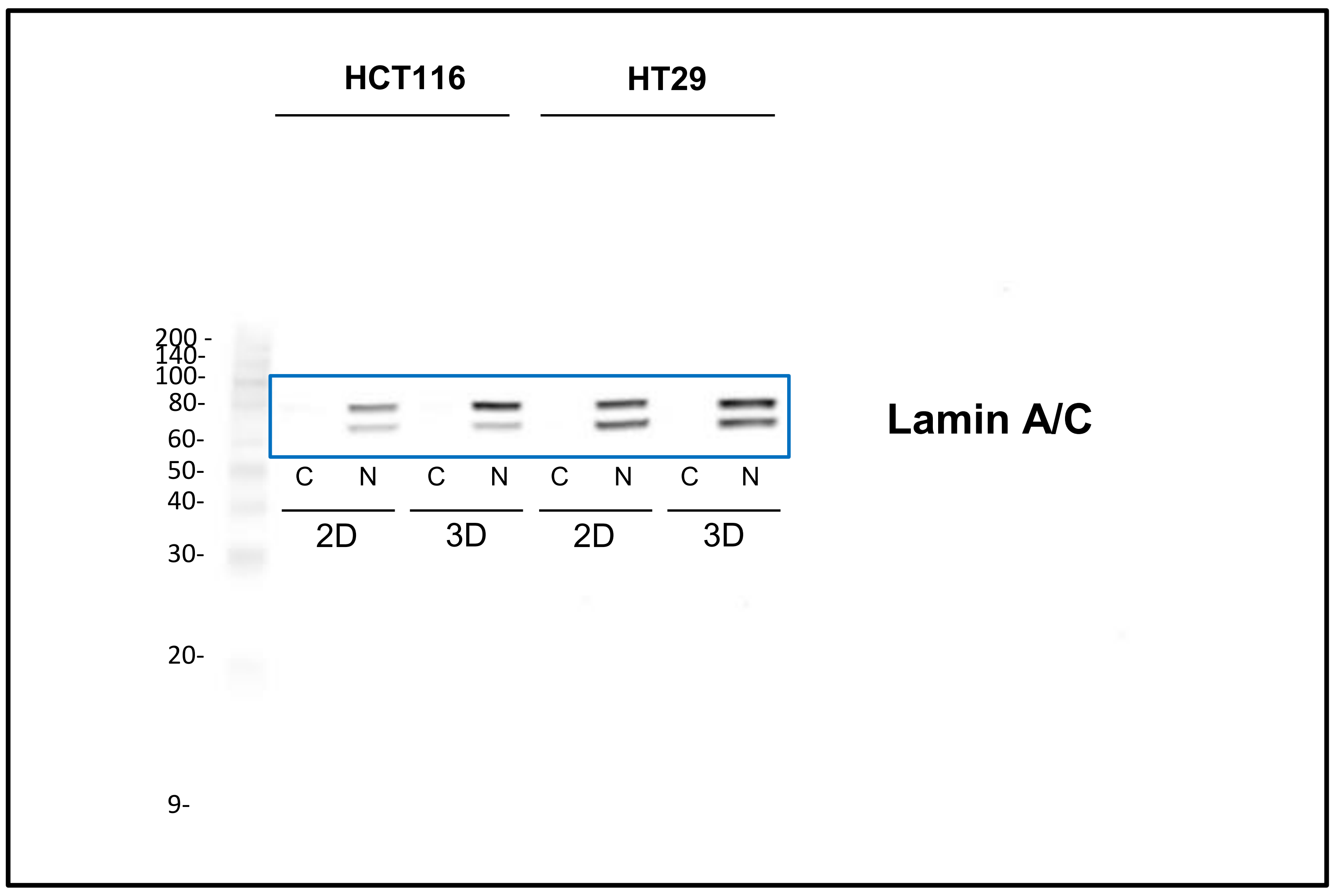

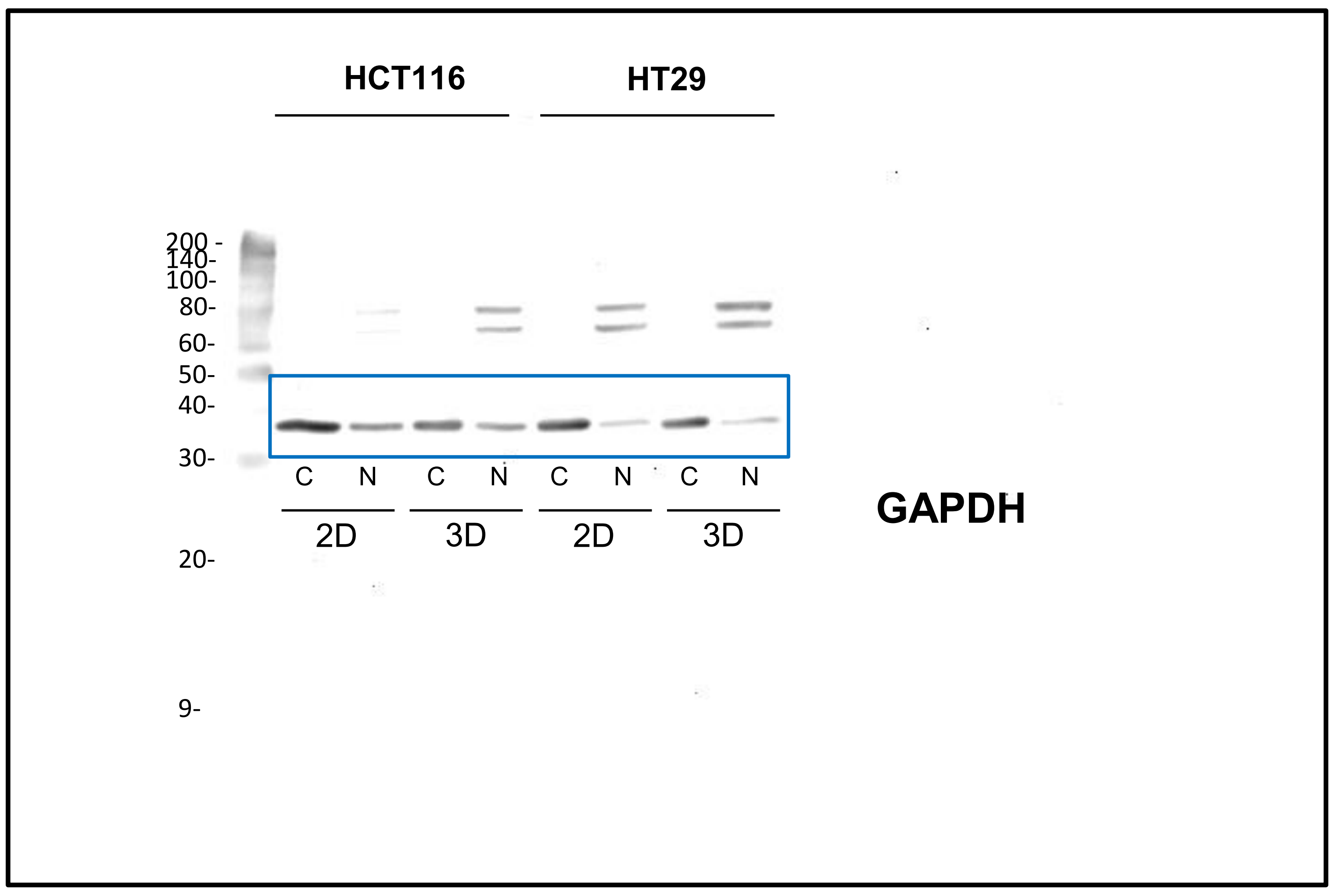

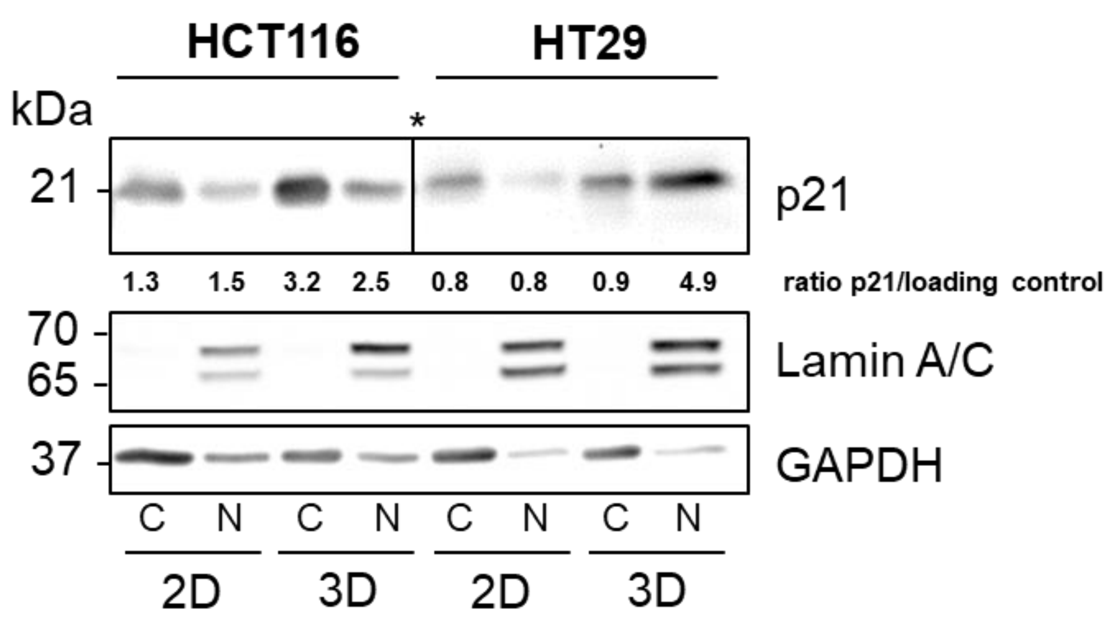

Fig S3-western blot in manuscript

selected bands

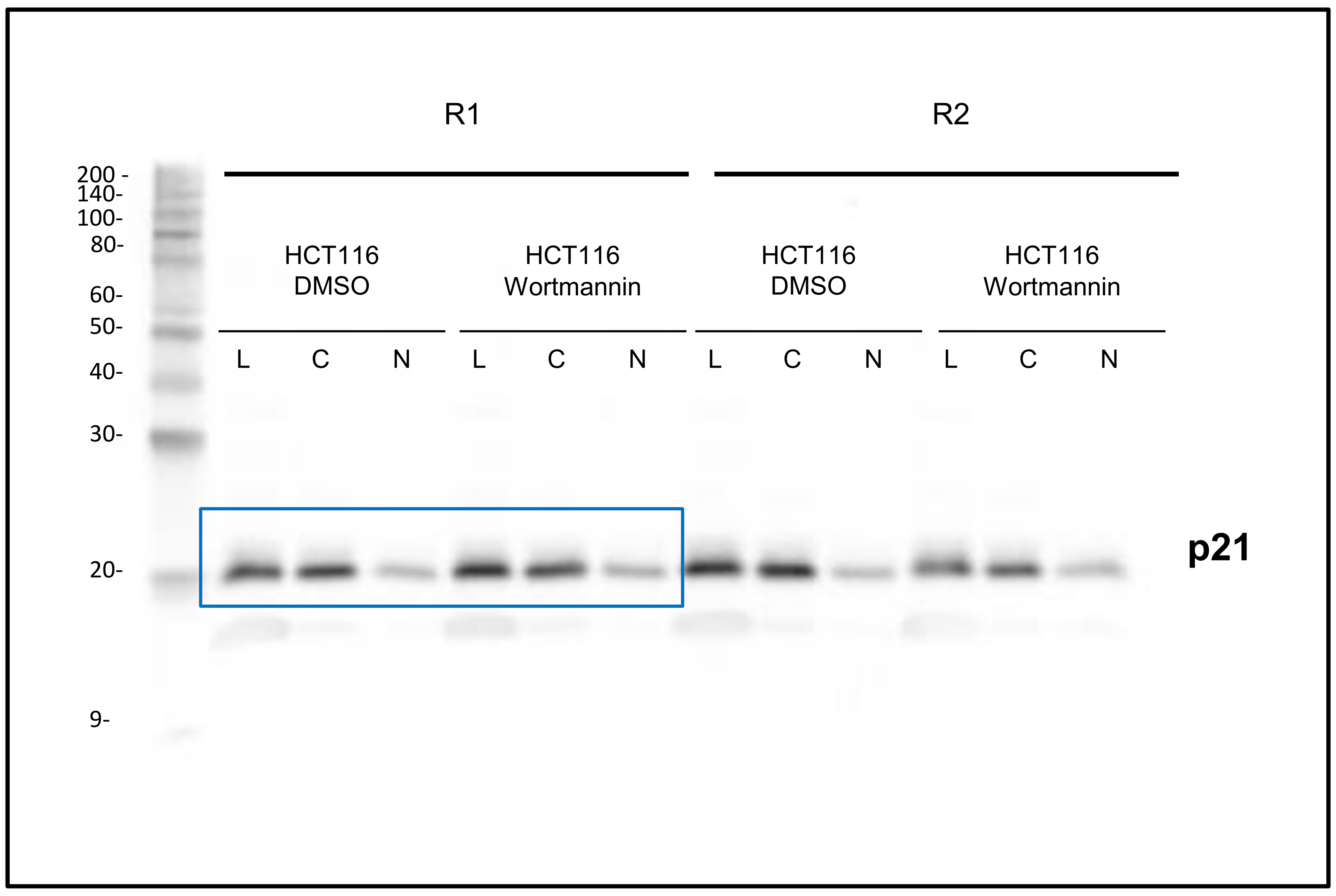

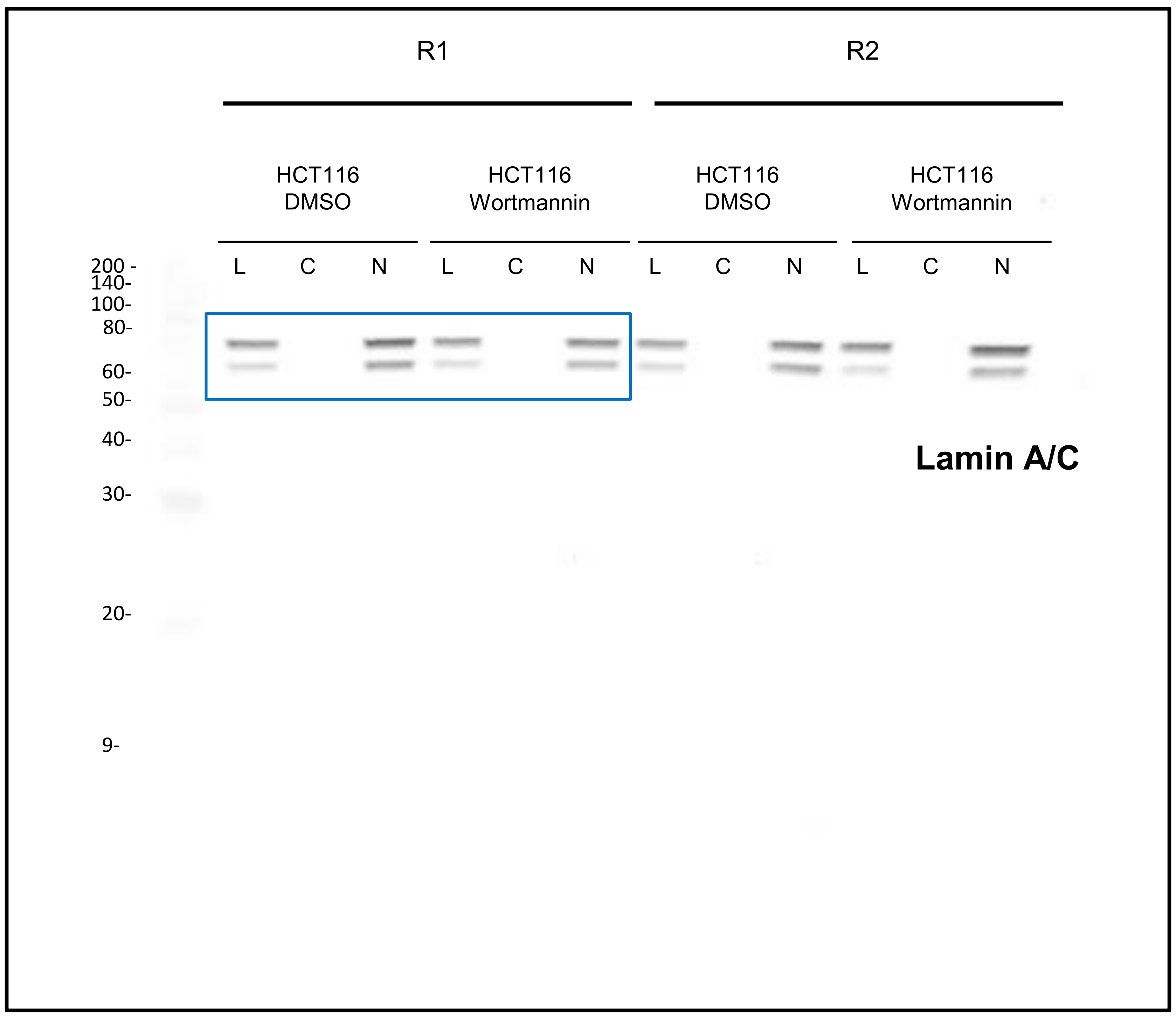

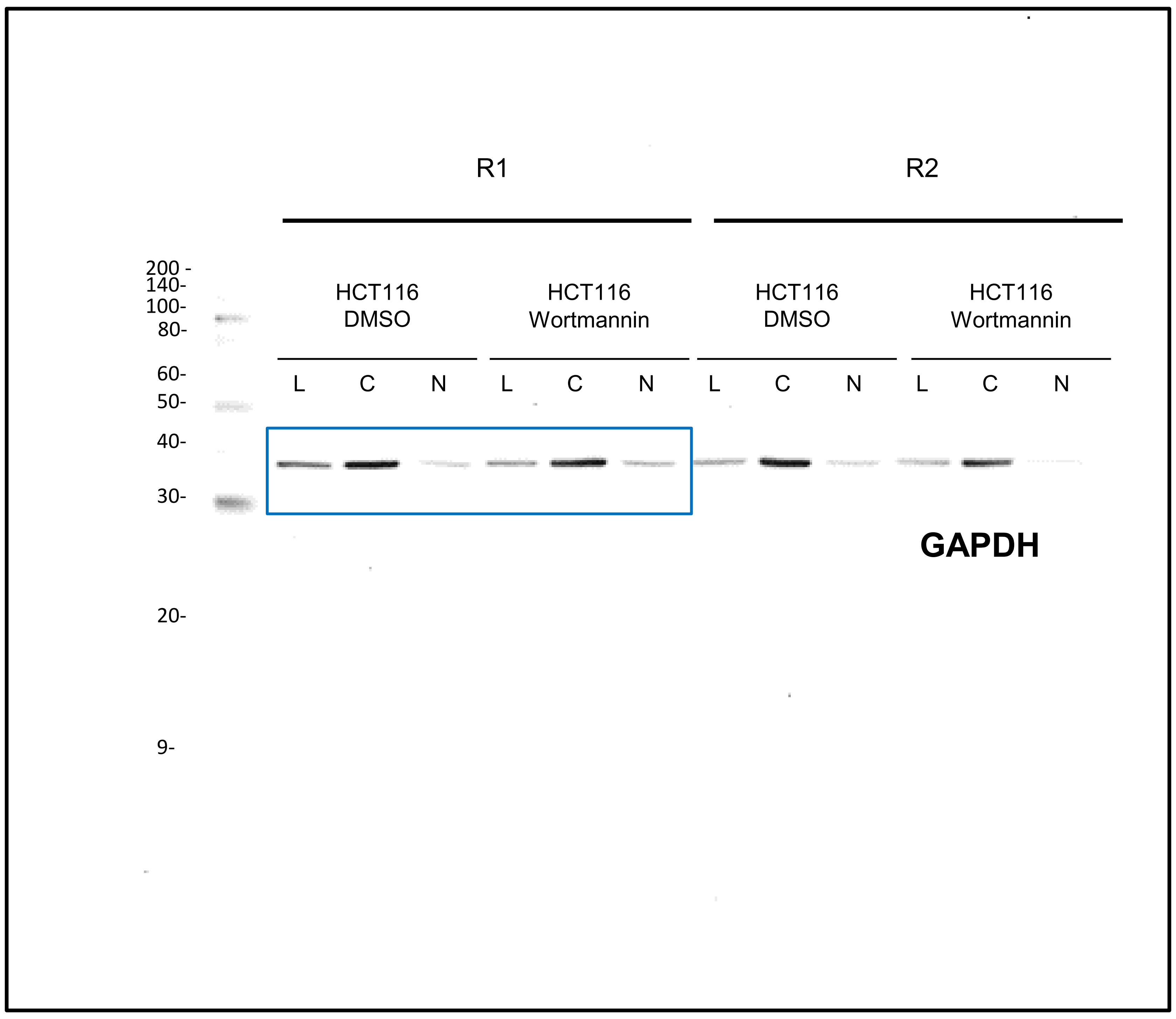

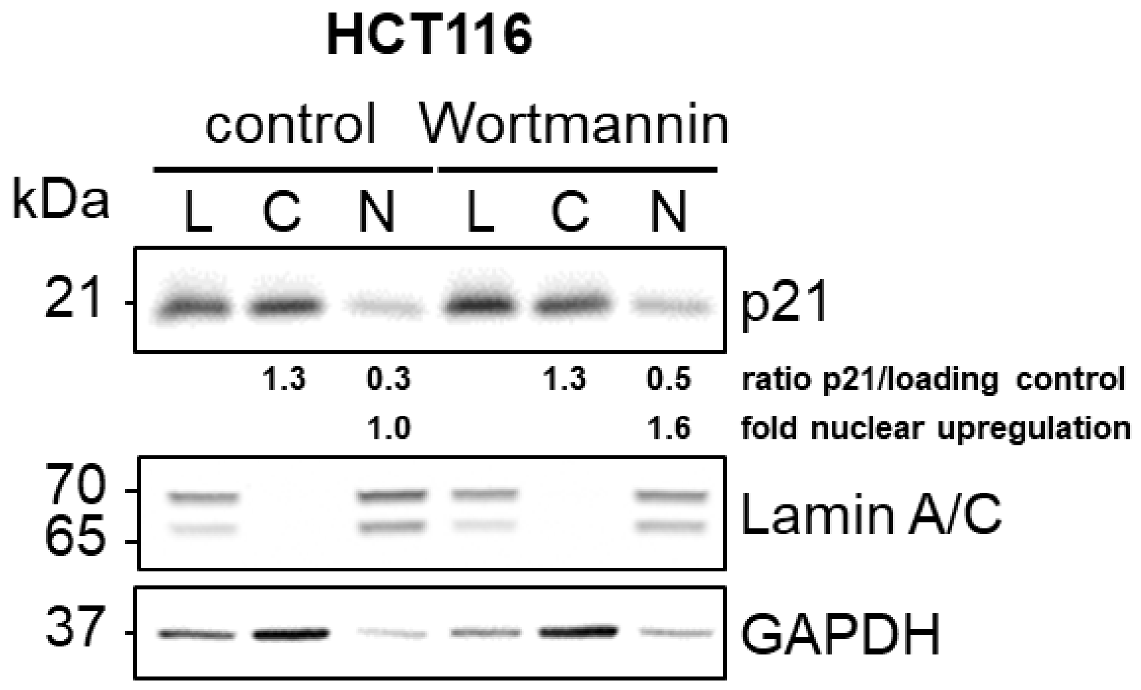

Fig S4-C-western blot in manuscript

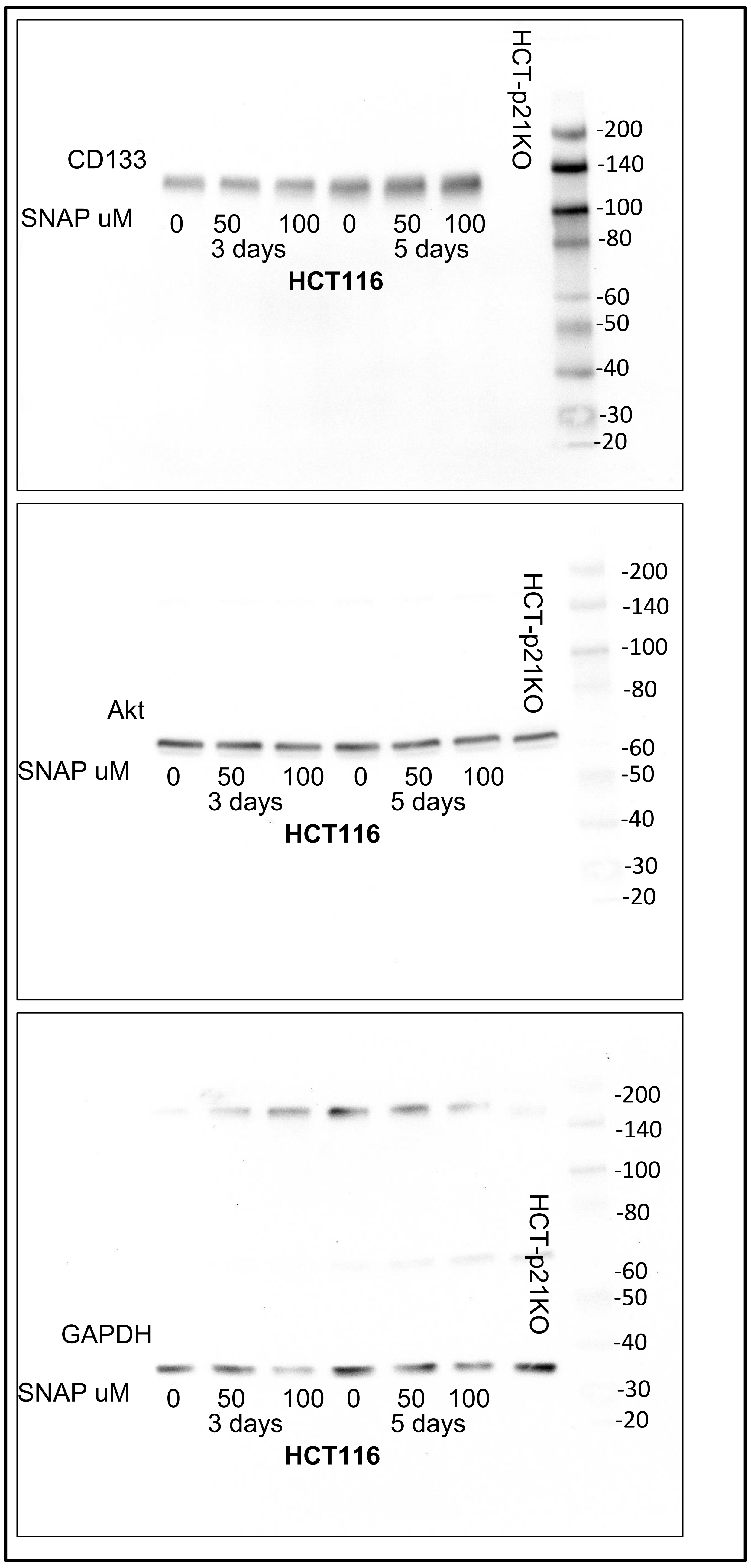

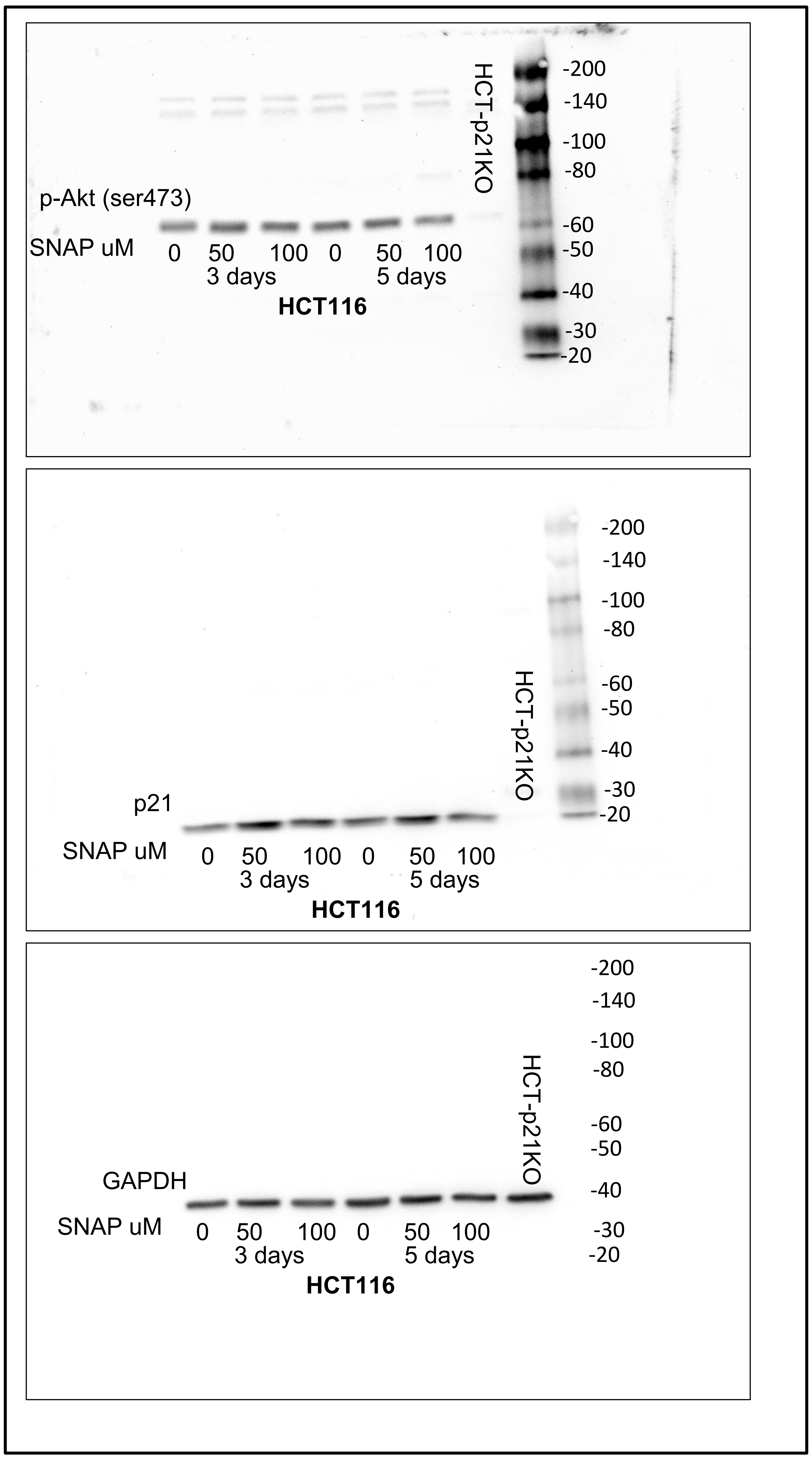

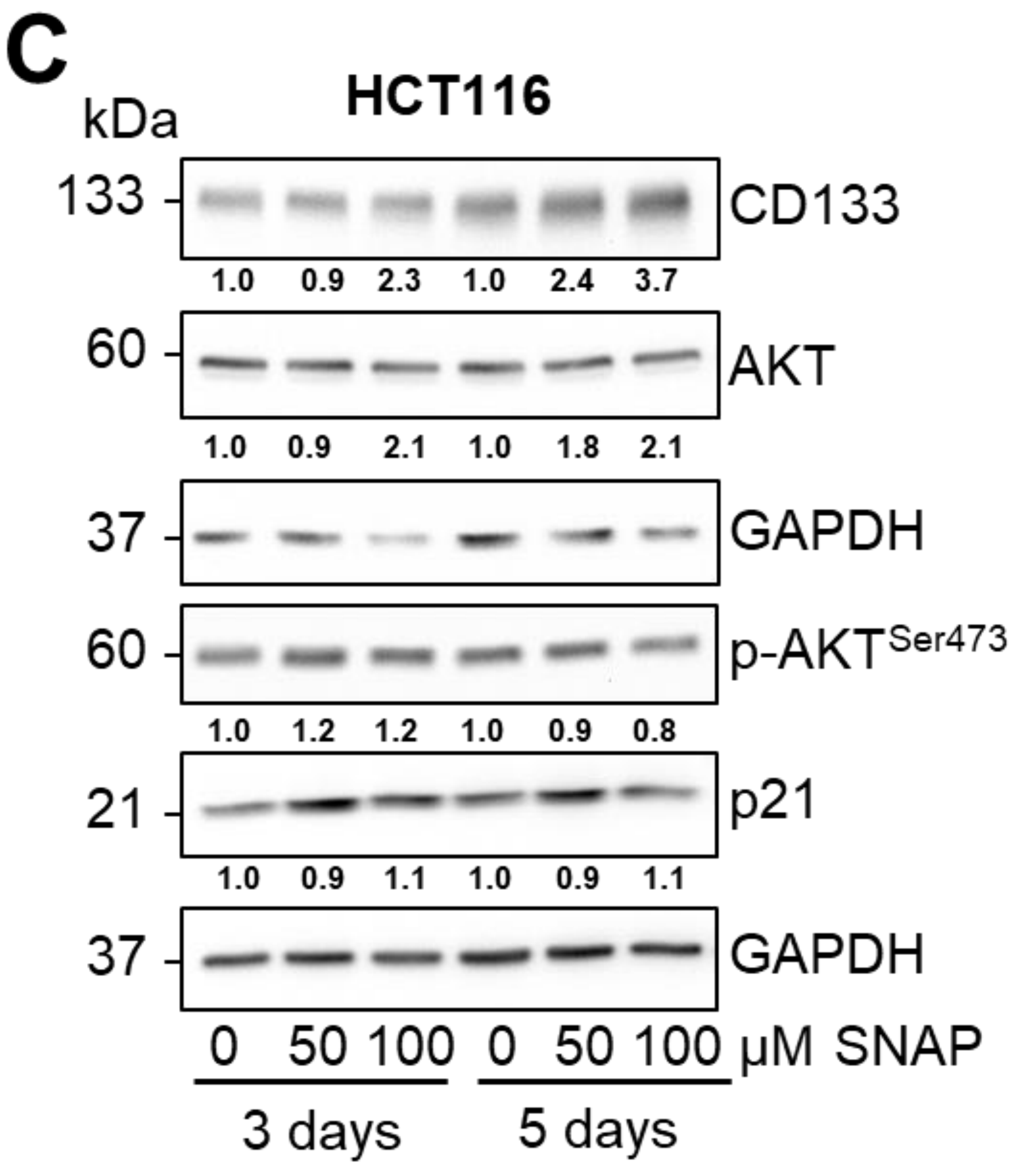

Fig S4-E-western blot in manuscript

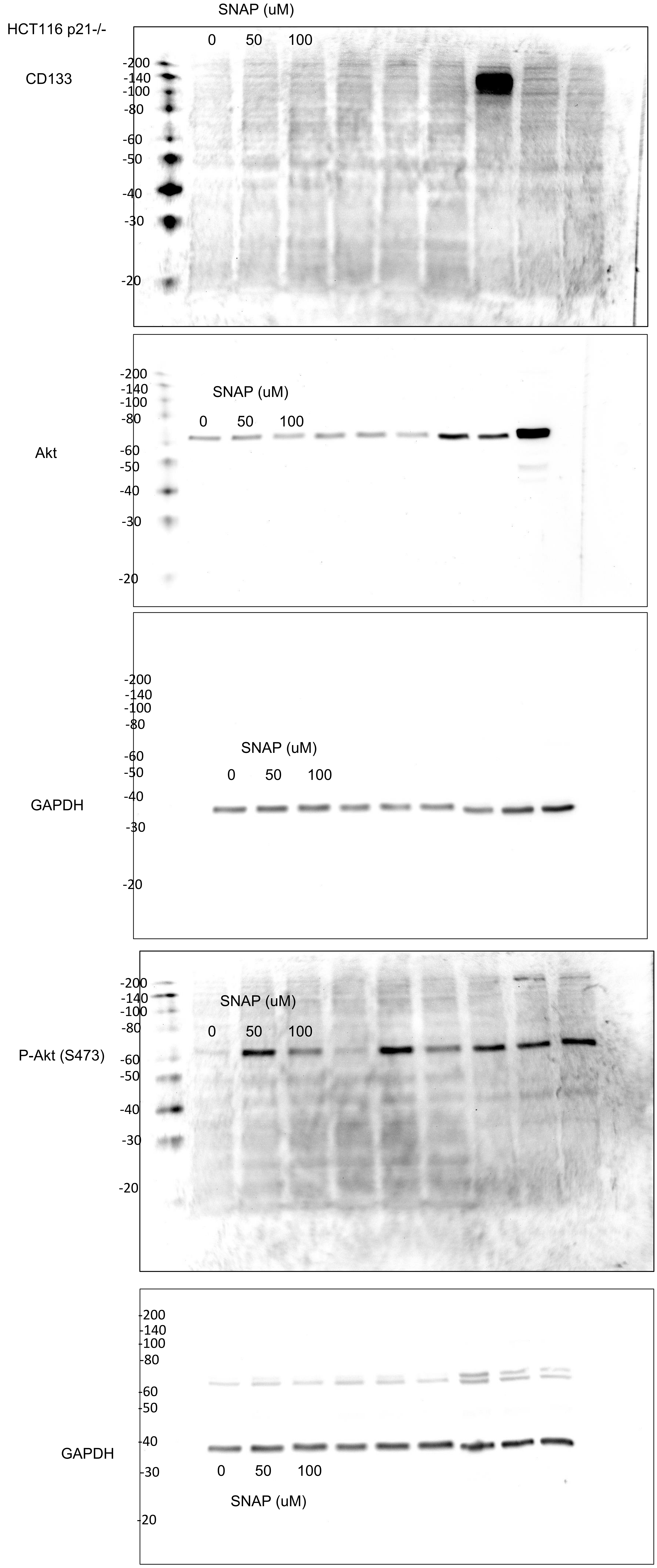

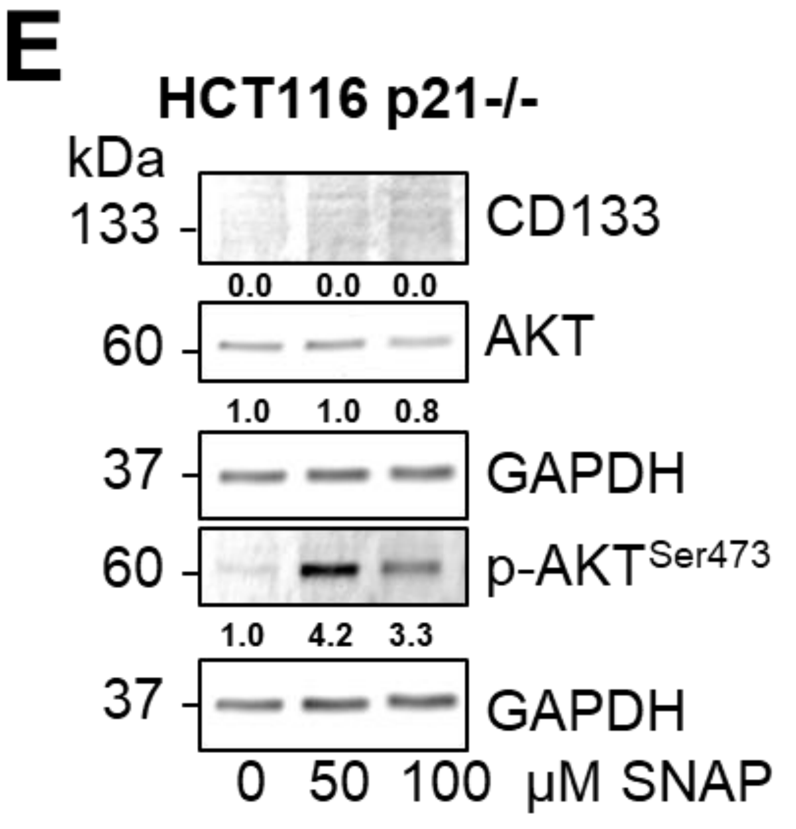

Fig S4-F-western blot in manuscript

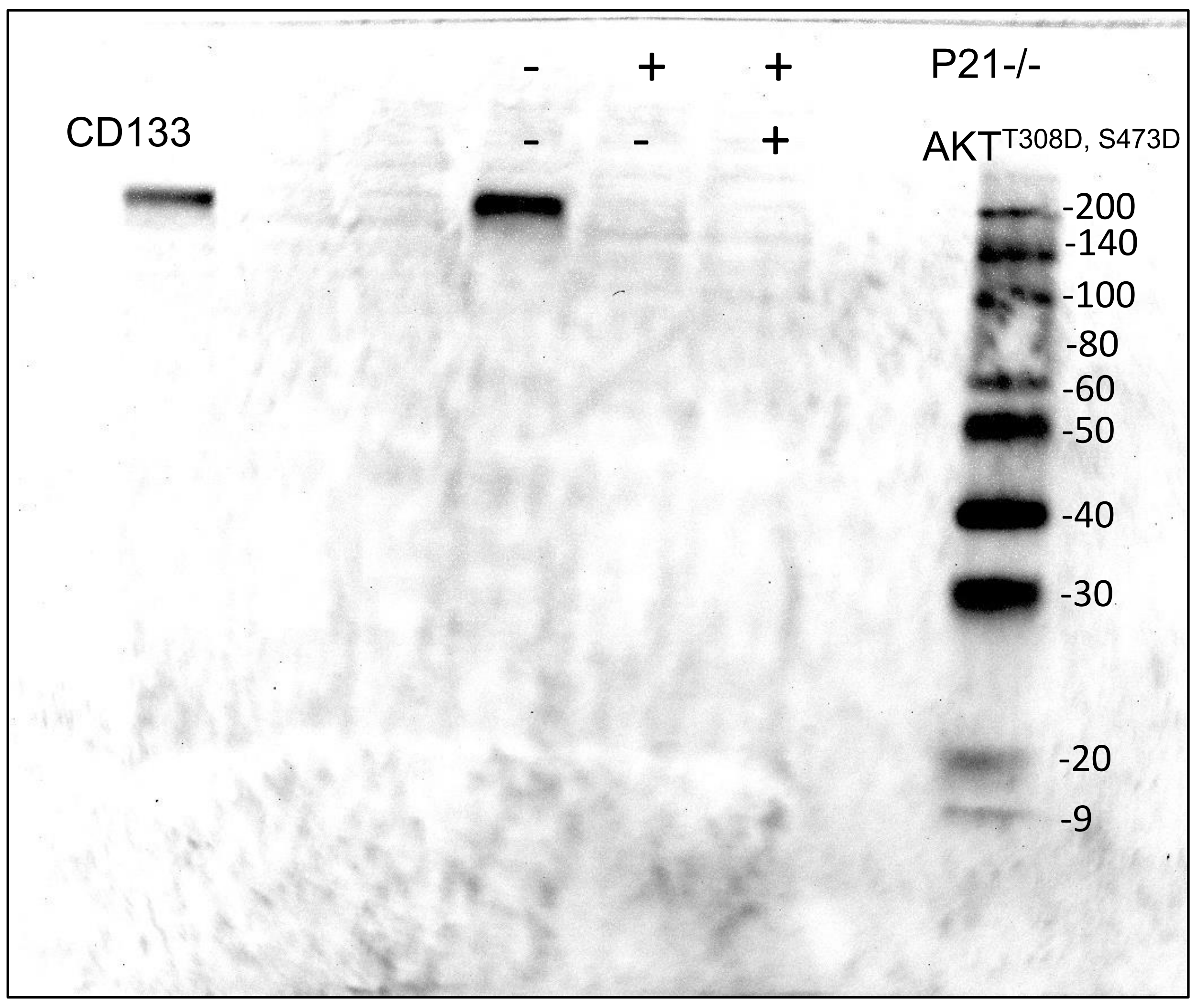

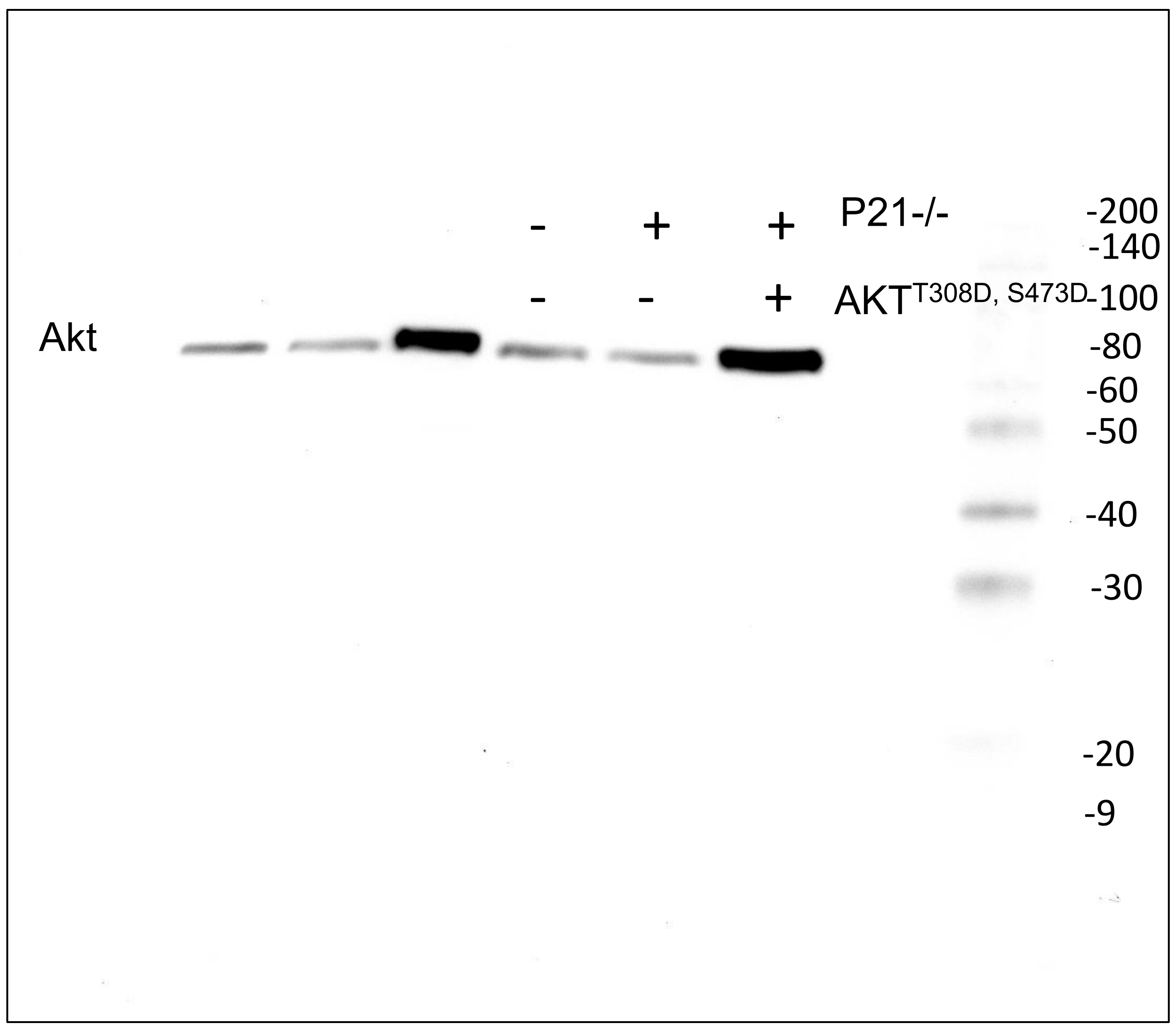

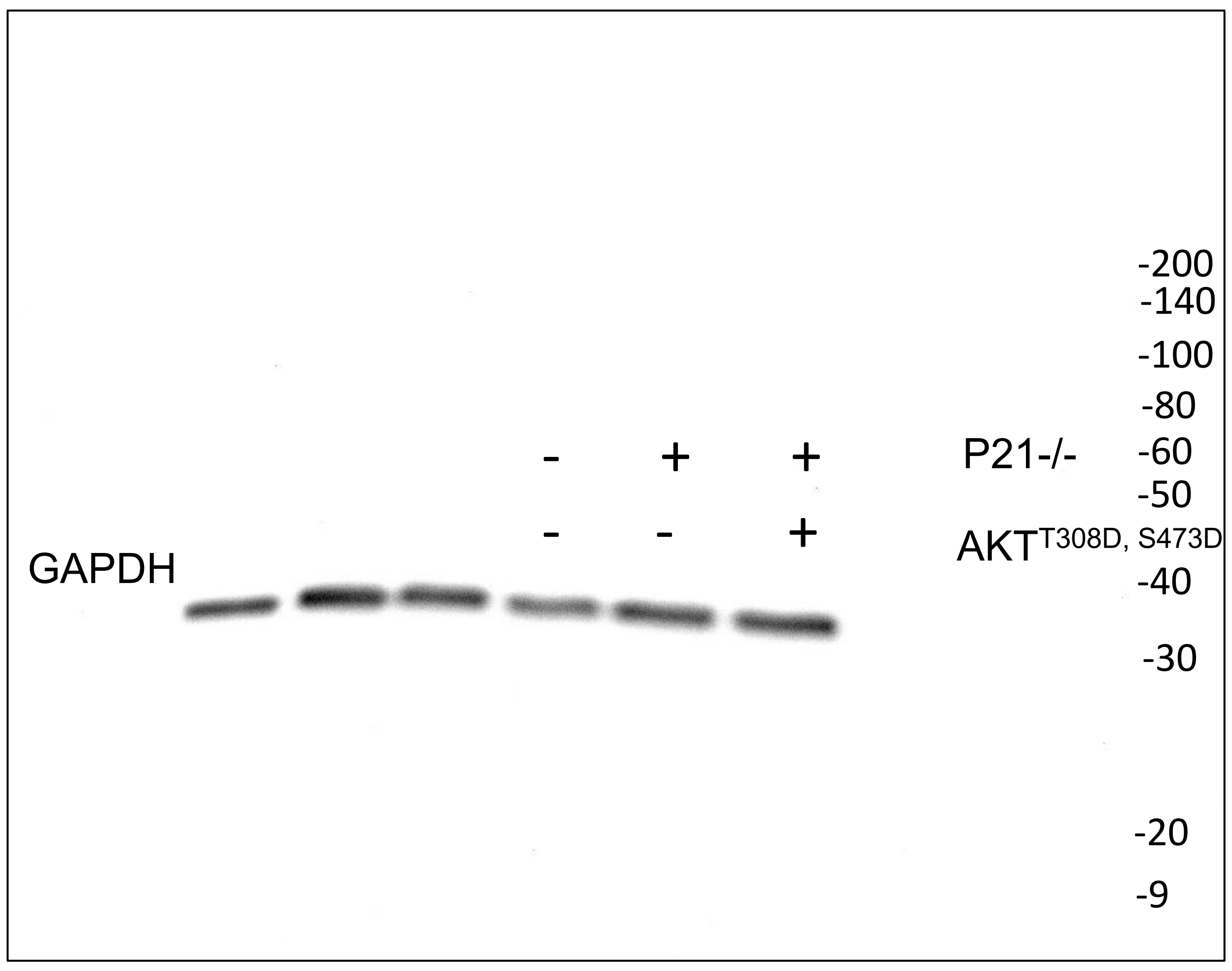

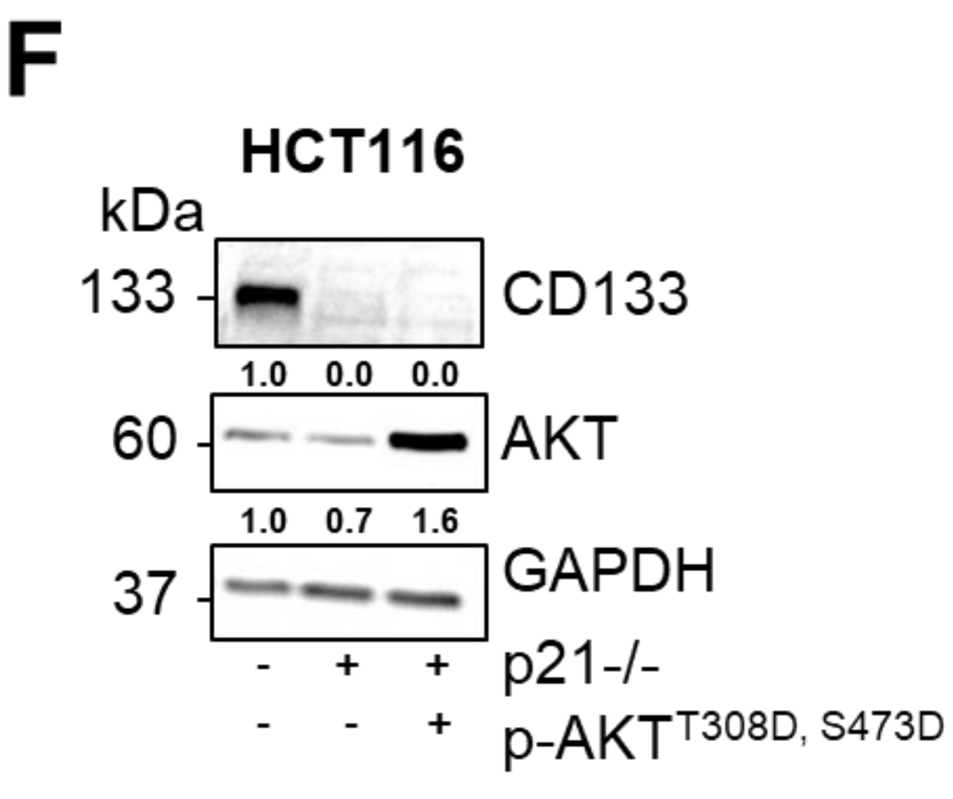

Fig S5-C-western blot in manuscript

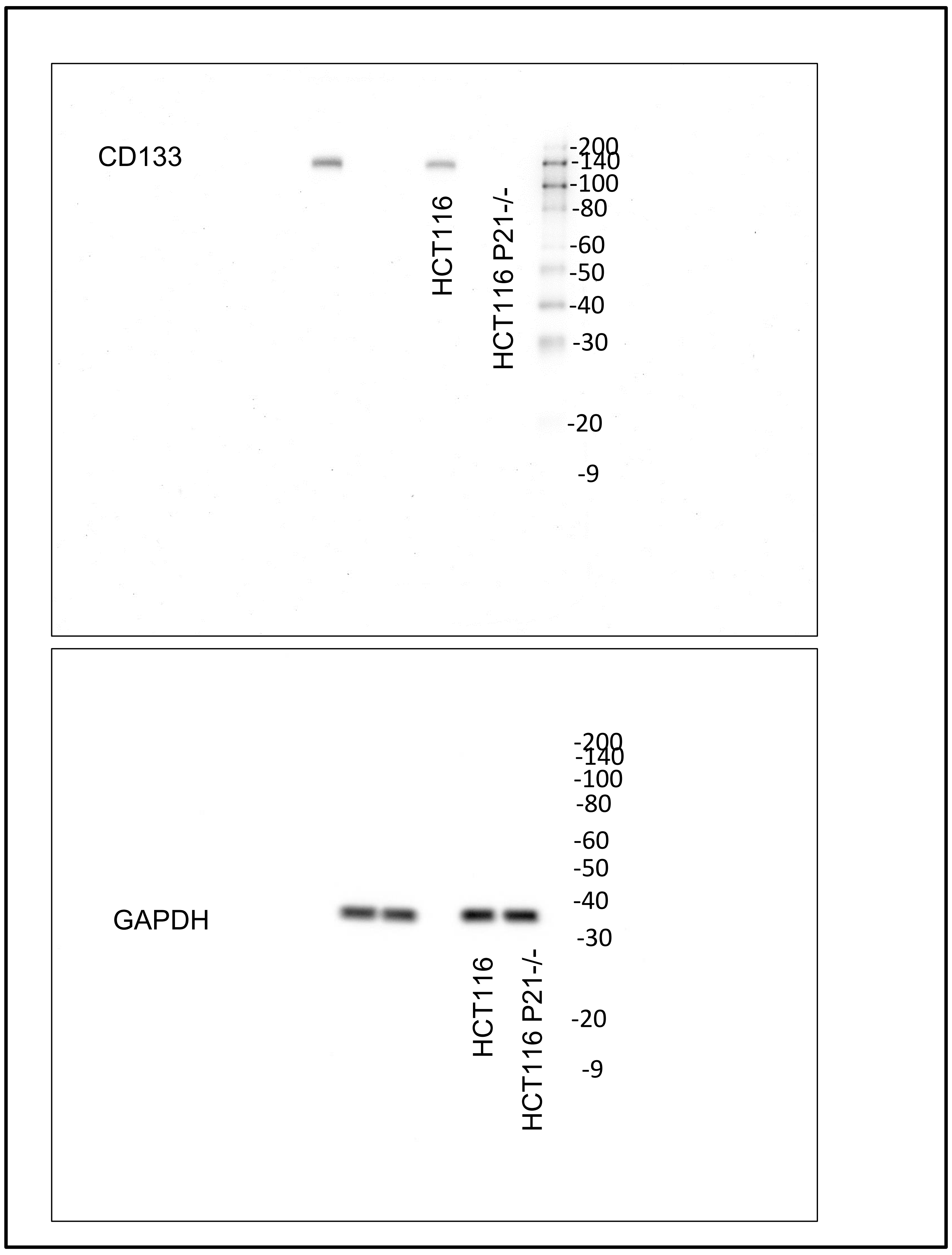

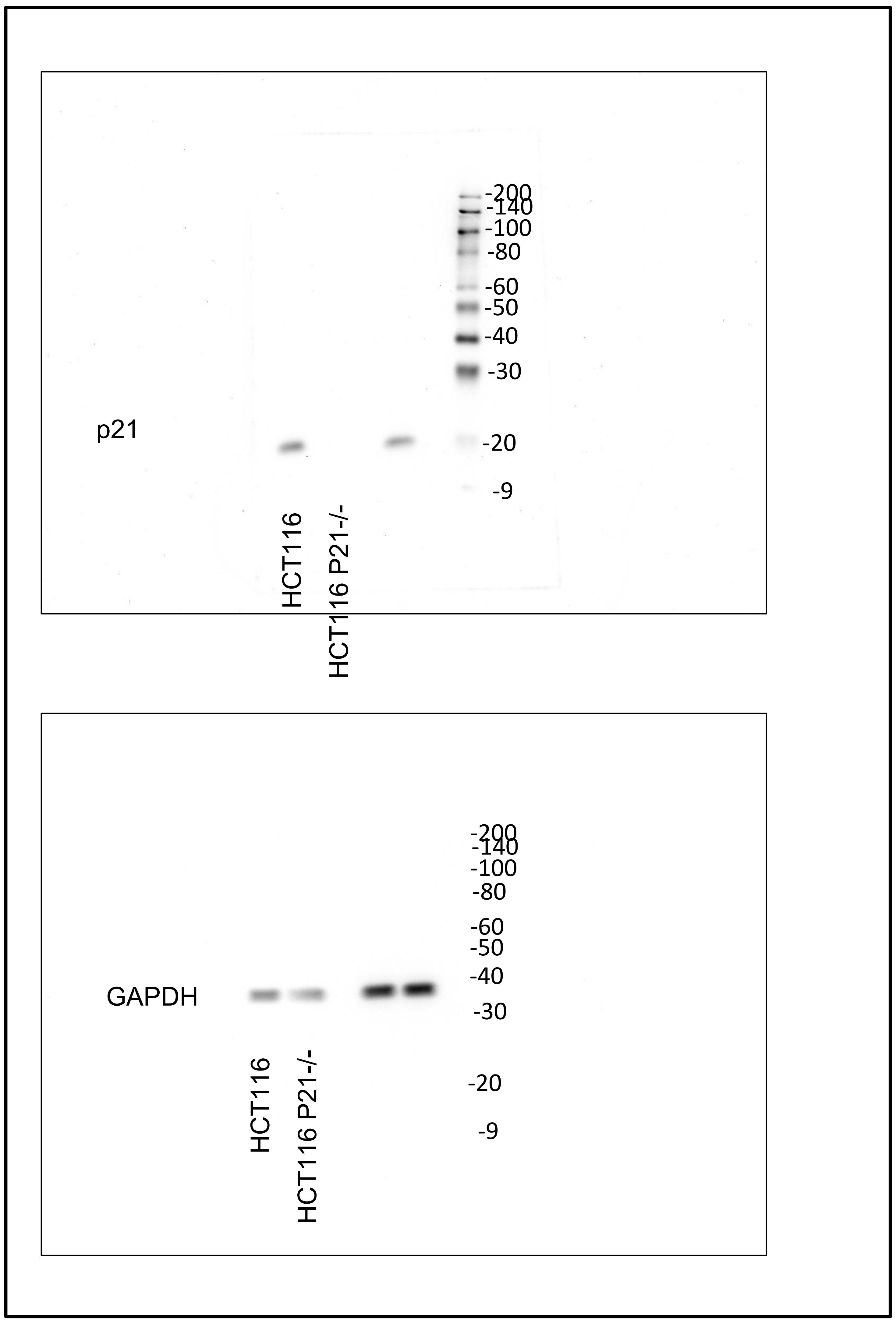

C

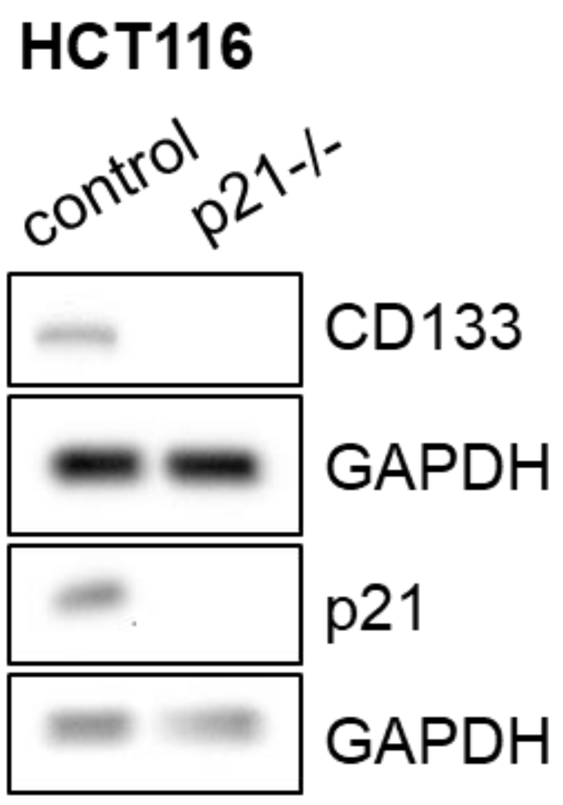

Fig S6-western blot in manuscript

selected bands

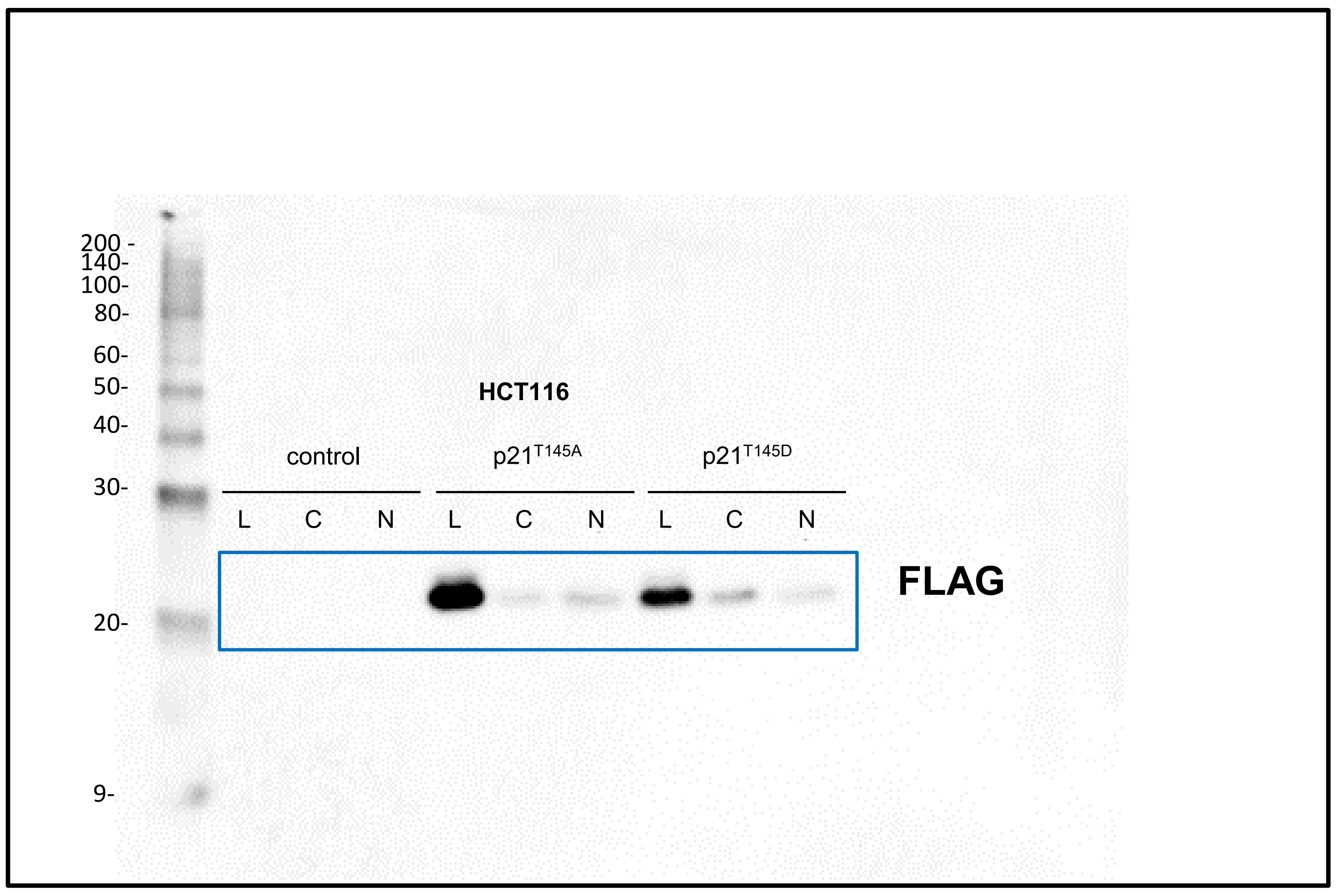

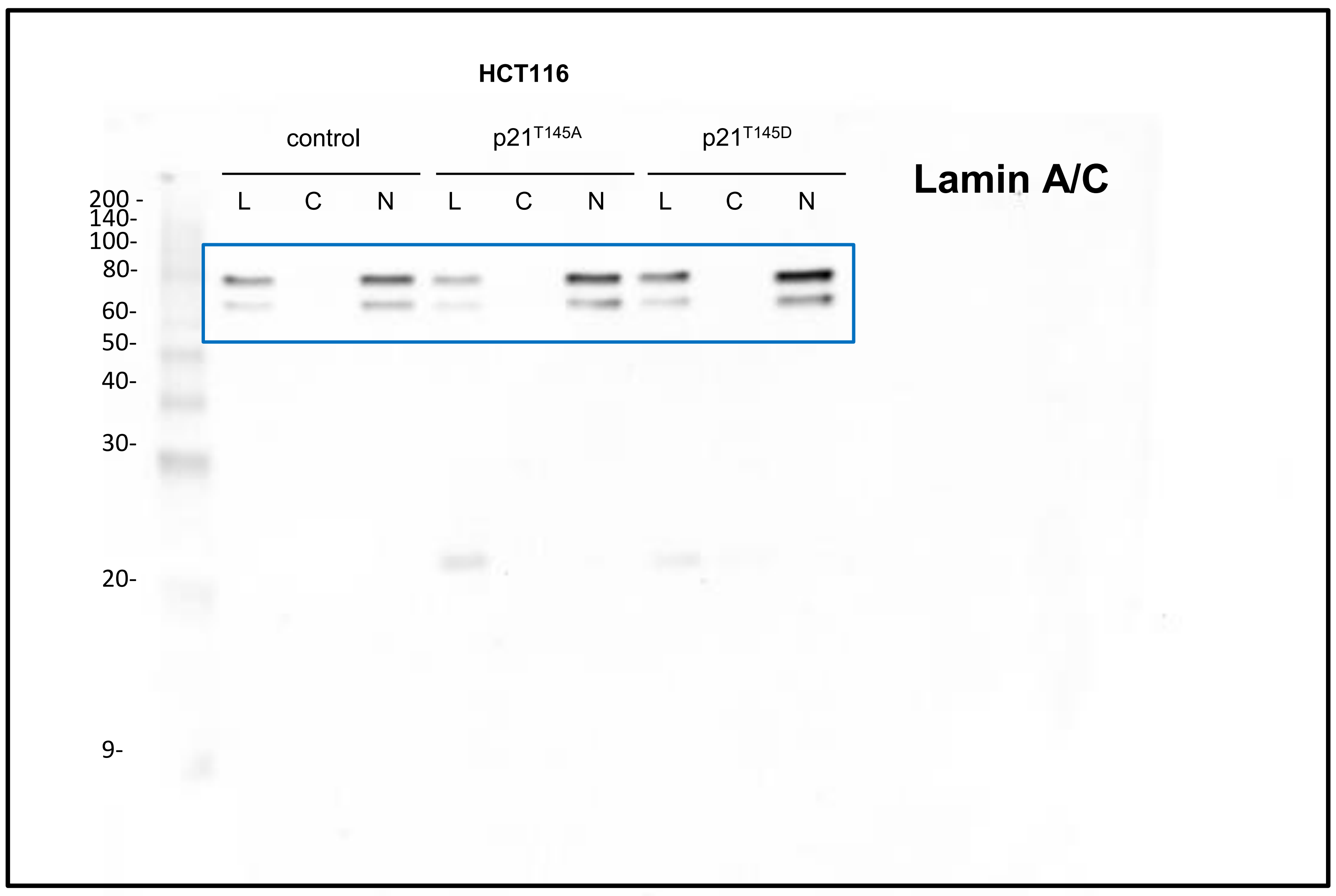

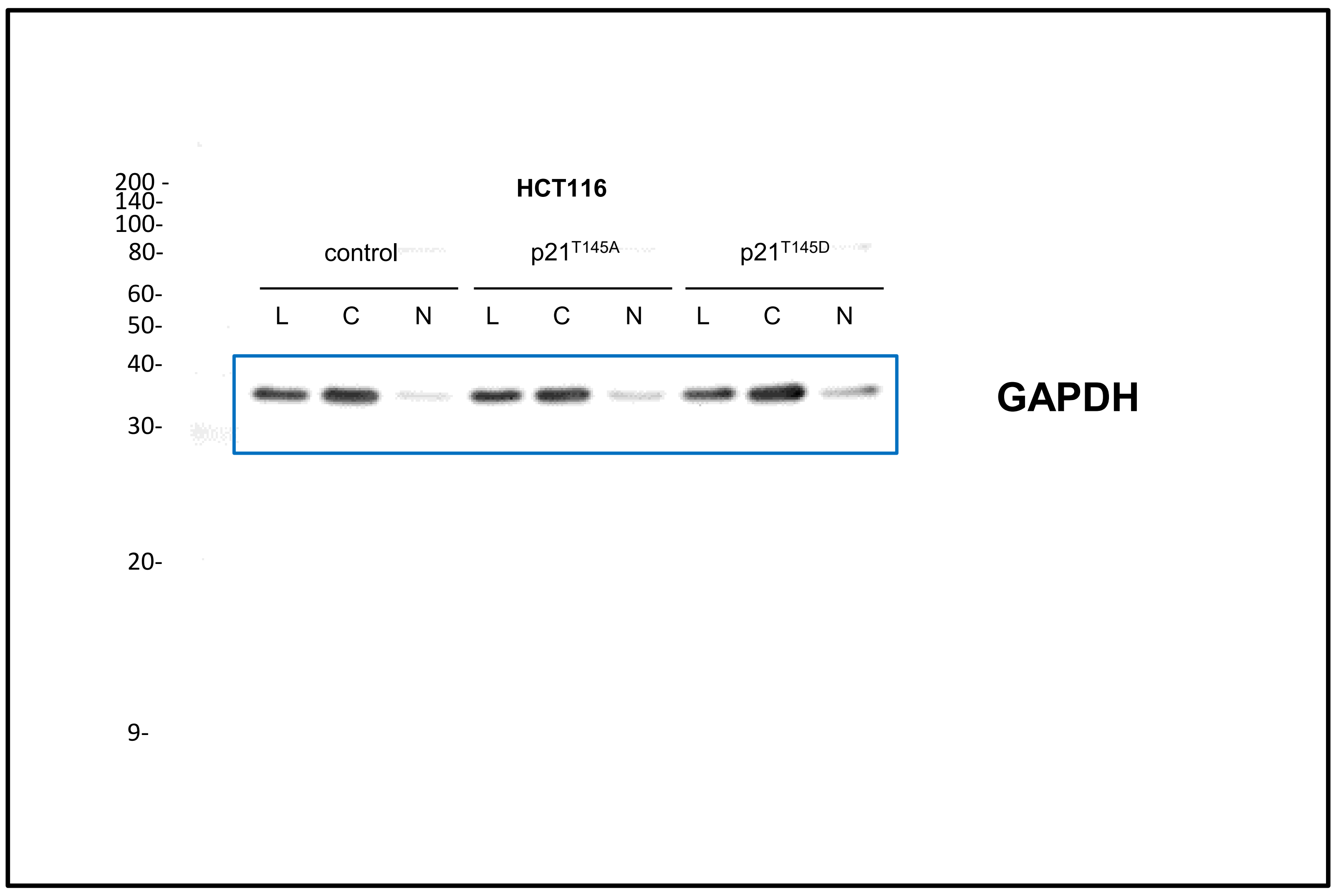

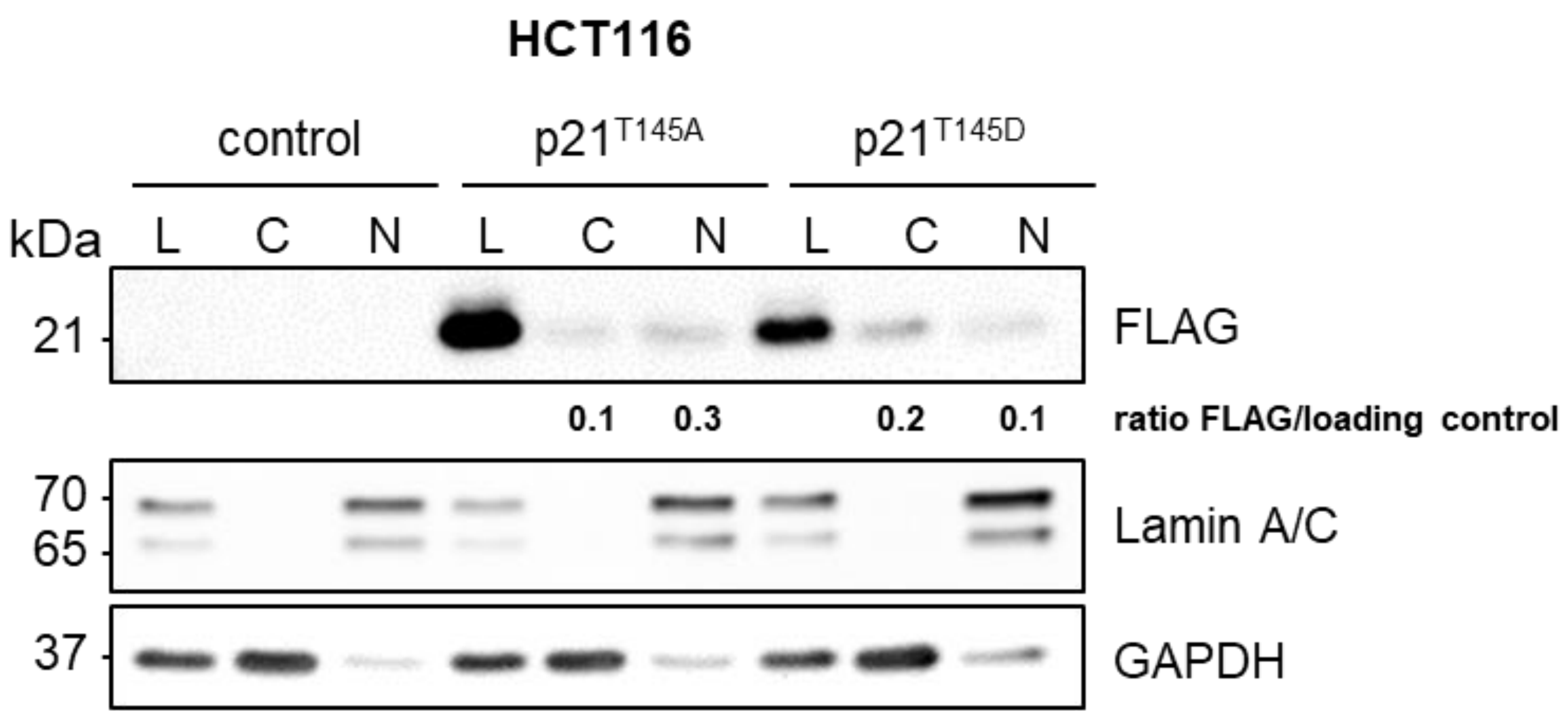

Supplement: Supplementary file 1 — Fig. S1. Subcellular fractionation of HCT116 and HT29 cells grown in 2D and 3D conditions. Fig. S2. Multicellular limiting dilution spheroid assay of transfected hyperphosphorylated AKT cell for 5 days of assay duration. Fig. S3. Wortmannin treatment increased nuclear p21. Fig. S4. Nitric oxide promoted cancer stem cell phenotypes in HCT116 cells in a p21‐dependent manner. Fig. S5. Cancer stem cell properties of HCT116 p21−/− cells. Fig. S6. Transfection of HCT116 cells with p21T145D and p21T145A induced cytoplasmic and nuclear localization of p21, respectively. Fig. S7. Effect of hyperphosphorylated p21T145D and unphosphorylated p21T145A on cancer stem cell properties. Fig. S8. Computational modelling of ERK2‐mediated phosphorylation of p21 and its interaction with IκB/NFκB p50/p65 complex. Table S1. Patient characteristics–comparison with cytoplasmic p21. [file MOL2-20-1022-s001.zip › misc/mol270150-sup-0010-WesternblotRQCR2.pdf]
