# Supplementary material for: Cytoplasmic p21 promotes stemness of colon cancer cells via activation of the NFκB pathway
Source: Mol Oncol. 2025 Nov 3;20(4):1022–40. doi: 10.1002/1878-0261.70150 (PMC13060645; doi:10.1002/1878-0261.70150)
Supplement: Supplementary file 1 — Fig. S1. Subcellular fractionation of HCT116 and HT29 cells grown in 2D and 3D conditions. Fig. S2. Multicellular limiting dilution spheroid assay of transfected hyperphosphorylated AKT cell for 5 days of assay duration. Fig. S3. Wortmannin treatment increased nuclear p21. Fig. S4. Nitric oxide promoted cancer stem cell phenotypes in HCT116 cells in a p21‐dependent manner. Fig. S5. Cancer stem cell properties of HCT116 p21−/− cells. Fig. S6. Transfection of HCT116 cells with p21T145D and p21T145A induced cytoplasmic and nuclear localization of p21, respectively. Fig. S7. Effect of hyperphosphorylated p21T145D and unphosphorylated p21T145A on cancer stem cell properties. Fig. S8. Computational modelling of ERK2‐mediated phosphorylation of p21 and its interaction with IκB/NFκB p50/p65 complex. Table S1. Patient characteristics–comparison with cytoplasmic p21. [file MOL2-20-1022-s001.zip › misc/Supplementary Figure Legends-R-UPDATE-02.docx]

**Supplementary Figure Legends**

**Figure S1: Subcellular fractionation of HCT116 and HT29 cells grown in 2D and 3D conditions.** HCT116 and HT29 cells were grown for 24 h (2D) and 10 days (3D, as spheroids) and subjected to subcellular fractionation to extract cytoplasmic (C) and nuclear (N) proteins. Protein expression levels for p21 were determined by Western blotting. Lamin A/C and GAPDH (1:200,000) served as nuclear and cytoplasmic loading controls, respectively. For ratio calculation, p21 levels were calculated against the corresponding loading controls. The asterisk (*) indicates that all samples were loaded onto the same gel, but membrane was spliced. Representative blots of two independent experiments are shown.

**Figure S2:** **Multicellular limiting dilution spheroid assay of transfected hyperphosphorylated AKT cell for 5 days of assay duration.**The cancer stem cell capacity of hyperphosphorylated AKT (AKT^T308D, S473D^) transfected HCT116, and SW837 cells was determined by a multicellular limiting dilution (MCLD) spheroid assay in serum-free medium. (**A, B**) HCT116 and (**C, D**) SW837 cells were transfected with hyperphosphorylated AKT^T308D,S473D^ and then subjected to a MCLD spheroid assay in serum-free medium for 5 days, after which the spheroid diameter was determined. The representative scale bars are 250 µm. Representative images and the data of two independent experiments are shown. Statistical testing using a two-way ANOVA revealed that AKT^T308D,S473D^ transfected HCT116 spheroids were only significantly larger at higher cell numbers (2,000 to 250 cells/well; p values were ≤ 0.05) while the sizes did not differ from mock transfected spheroids for all conditions in AKT^T308D,S473D^ transfected SW837 cells.

**Figure S3: Wortmannin treatment increased nuclear p21.** HCT116 cells were treated with 1 µM Wortmannin (and the corresponding DMSO concentration; control) for 72 h and subsequently subjected to subcellular fractionation to extract whole cell lysate (L), cytoplasmic (C) and nuclear (N) proteins. Protein expression levels for p21 were determined by Western blotting. Lamin A/C and GAPDH (1:100,000) served as nuclear and cytoplasmic loading controls, respectively. For ratio calculation, p21 levels were calculated against the corresponding loading controls and fold nuclear upregulation was obtained by dividing the normalized nuclear ratio by the normalized cytoplasmic ratio. Representative blots of two independent experiments are shown.

**Figure S4: Nitric oxide promoted cancer stem cell phenotypes in HCT116 cells in a p21-dependent manner.** (**A**) HCT116 cells treated with 50 µM SNAP (nitric oxide donor) for 5 days were subjected to a MCLD spheroid assay for 10 days in serum-free medium. The representative scale bars are 250 µm. Representative images of two independent experiments are shown. (**B**) The diameter and number of spheroids obtained in (**A**) were determined by ImageJ (n=2). Statistical testing using a two-way ANOVA revealed that NO-treated HCT116 spheroids were in general significantly larger in size (at 2,000 to 500 cells/well and at 125 cells/well) compared to control spheroids (p values were ≤ 0.05). (**C**) HCT116 cells treated with 50 µM or 100 µM SNAP for 3 days or 5 days were subjected to Western blot analysis of the protein expression levels of CD133, AKT, p-AKT^S473^, and p21. The blots were re-probed with GAPDH (1:50,000) to confirm equal loading of the samples. Representative blots of two independent experiments are shown. (**D**) HCT116 cells treated with 50 µM SNAP for 5 days were stained with rabbit anti-p21 antibody and mouse anti-CD133 antibody, followed by anti-rabbit Alexa Fluor 488-conjugated antibody and anti-mouse Alexa Fluor 555-conjugated antibody to visualize p21 (green) and CD133 (red). The cell nuclei were visualized by staining with Hoechst 33342 (blue). The representative scale bar is 100 µm. Representative images of two independent experiments are shown. (**E**) After HCT116 p21-/- cells were treated with SNAP (50 and 100 µM) for 5 days, the protein expression levels of CD133, AKT, and p-AKT^Ser473^ were determined by Western blotting. The blots were re-probed with GAPDH (1:50,000) to confirm equal loading of the samples. Representative blots of two independent experiments are shown. (**F**) HCT116 p21-/- cells were transfected with hyperphosphorylated AKT (AKT^T308D, S473D^), and the expression of CD133 and AKT was evaluated by Western blotting and compared between HCT116 and HCT116 p21-/- cells. The blot was re-probed with GAPDH (1:50,000) to confirm equal loading of the samples. Representative blots of two independent experiments are shown.

**Figure S5: Cancer stem cell properties of HCT116 p21-/- cells.**(**A**) HCT116 p21-/- protein expression level of CD133 and p21 were determined by Western blotting. The blots were re-probed with GAPDH (1:50,000) to confirm equal loading of the samples. Representative blots of two independent experiments are shown. (**B**) HCT116 p21-/- cells were stained with mouse anti-CD133 antibody and rabbit anti-F-actin antibody, then followed by anti-mouse Alexa Fluor 555-conjugated antibody and anti-rabbit Alexa Fluor 488-conjugated antibody to visualize CD133 (red) and p21 (green). The cell nuclei were visualized by staining with Hoechst 33342 (blue). The representative scale bar is 100 µm. Representative images of two independent experiments are shown. (**C, D**) The cancer stem cell capacity of p21-knockout HCT116 (HCT116 p21-/-) cells was determined by a multicellular limiting dilution (MCLD) spheroid assay in serum-free medium for 5 days, after which the spheroid diameter was determined. The representative scale bars are 250 µm. Representative images and the data of two independent experiments are shown. Statistical testing using a two-way ANOVA revealed that HCT116 spheroids were significantly larger in size compared to HCT116 p21-/- that did not form spheroids at all (p values were ≤ 0.05).

**Figure S6: Transfection of HCT116 cells with** **p21^T145D^ and p21^T145A^ induced cytoplasmic and nuclear localization of p21, respectively.** HCT116 cells were transfected with the phosphomimetic p21 form (p21^T145D^) and the unphosphorylated p21 form (p21^T145A^) for 24 h. Lipofectamine 3000 served as transfection control. After transfection, cells were subjected to subcellular fractionation to extract whole cell lysate (L), cytoplasmic (C) and nuclear (N) proteins. Protein expression levels for FLAG (transfected p21 only; marker present on both plasmids) were determined by Western blotting. Lamin A/C and GAPDH (1:200,000) served as nuclear and cytoplasmic loading controls, respectively. For ratio calculation, FLAG levels were calculated against the corresponding loading controls. Representative blots of two independent experiments are shown.

**Figure S7: Effect of hyperphosphorylated p21^T145D^ and unphosphorylated p21^T145A^ on cancer stem cell properties.**

(**A, B**) HCT119 and (**C, D**) SW837 colorectal cancer cell lines were transfected with hyperphosphorylated p21^T145D^ (cytoplasmic localized form of p21) or unphosphorylated p21^T145A^ (nuclear localized form of p21) and then subjected to a MCLD spheroid assay in serum-free medium for 5 days, after which the diameter and number of spheroids were determined. The representative scale bars are 250 µm. Representative images and the data of two independent experiments are shown. Statistical testing using a two-way ANOVA revealed that p21^T145D^ transfected HCT116 spheroids were only significantly larger at higher cell numbers (2,000 to 125 cells/well; p values were ≤ 0.05) while the sizes did not differ from mock transfected spheroids for all conditions in p21^T145^ transfected SW837 cells. (**E, F**) SW837 cells transfected with hyperphosphorylated AKT^T308D,S473D^ or hyperphosphorylated p21^T145D^ were subjected to evaluate the expression level of stemness related transcription factors by human pluripotent stem cell array (n=1). (**E**) Dot plot membrane and coordinates of the stem cell assay with two dots per marker. (**F**) The relative protein levels of stemness related transcription factors of the dot plot given in (**E**) of SW837 cells transfected with hyperphosphorylated AKT^T308D,S473D^ or hyperphosphorylated p21^T145D^ compared to the mock control. Dot plot signals were quantified by densitometry using ImageJ, and mean values were normalized to the mean of the mock control for each marker. Ctrl: control.

**Figure S8: Computational modelling of ERK2-mediated phosphorylation of p21 and its interaction with IκB/NFκB p50/p65 complex.** *In silico* analysis revealed that p21 phosphorylated at Thr57 and Ser130 by ERK2 localized to the periphery of the complex, suggesting no direct interaction between ERK2 and the IκB/NFκB pathway. Protein-protein interactions between IĸB/NFĸB and p21^T57^ and p21^S130^ showed an increase in the free energy of the NFĸB-IĸB complex from –201.05 kcal/mol to –154.75 kcal/mol and –160.47 kcal/mol, respectively, following interaction with the phosphorylated p21 molecule. Arrows indicate the corresponding phosphorylation sites of p21, Thr57 (*upper image*) and Ser130 (*lower image*).
