# Supplementary material for: Cytoplasmic p21 promotes stemness of colon cancer cells via activation of the NFκB pathway
Source: Mol Oncol. 2025 Nov 3;20(4):1022–40. doi: 10.1002/1878-0261.70150 (PMC13060645; doi:10.1002/1878-0261.70150)
Supplement: Supplementary file 1 — Fig. S1. Subcellular fractionation of HCT116 and HT29 cells grown in 2D and 3D conditions. Fig. S2. Multicellular limiting dilution spheroid assay of transfected hyperphosphorylated AKT cell for 5 days of assay duration. Fig. S3. Wortmannin treatment increased nuclear p21. Fig. S4. Nitric oxide promoted cancer stem cell phenotypes in HCT116 cells in a p21‐dependent manner. Fig. S5. Cancer stem cell properties of HCT116 p21−/− cells. Fig. S6. Transfection of HCT116 cells with p21T145D and p21T145A induced cytoplasmic and nuclear localization of p21, respectively. Fig. S7. Effect of hyperphosphorylated p21T145D and unphosphorylated p21T145A on cancer stem cell properties. Fig. S8. Computational modelling of ERK2‐mediated phosphorylation of p21 and its interaction with IκB/NFκB p50/p65 complex. Table S1. Patient characteristics–comparison with cytoplasmic p21. [file MOL2-20-1022-s001.zip › mol270150-sup-0008-FigureS8.pdf]

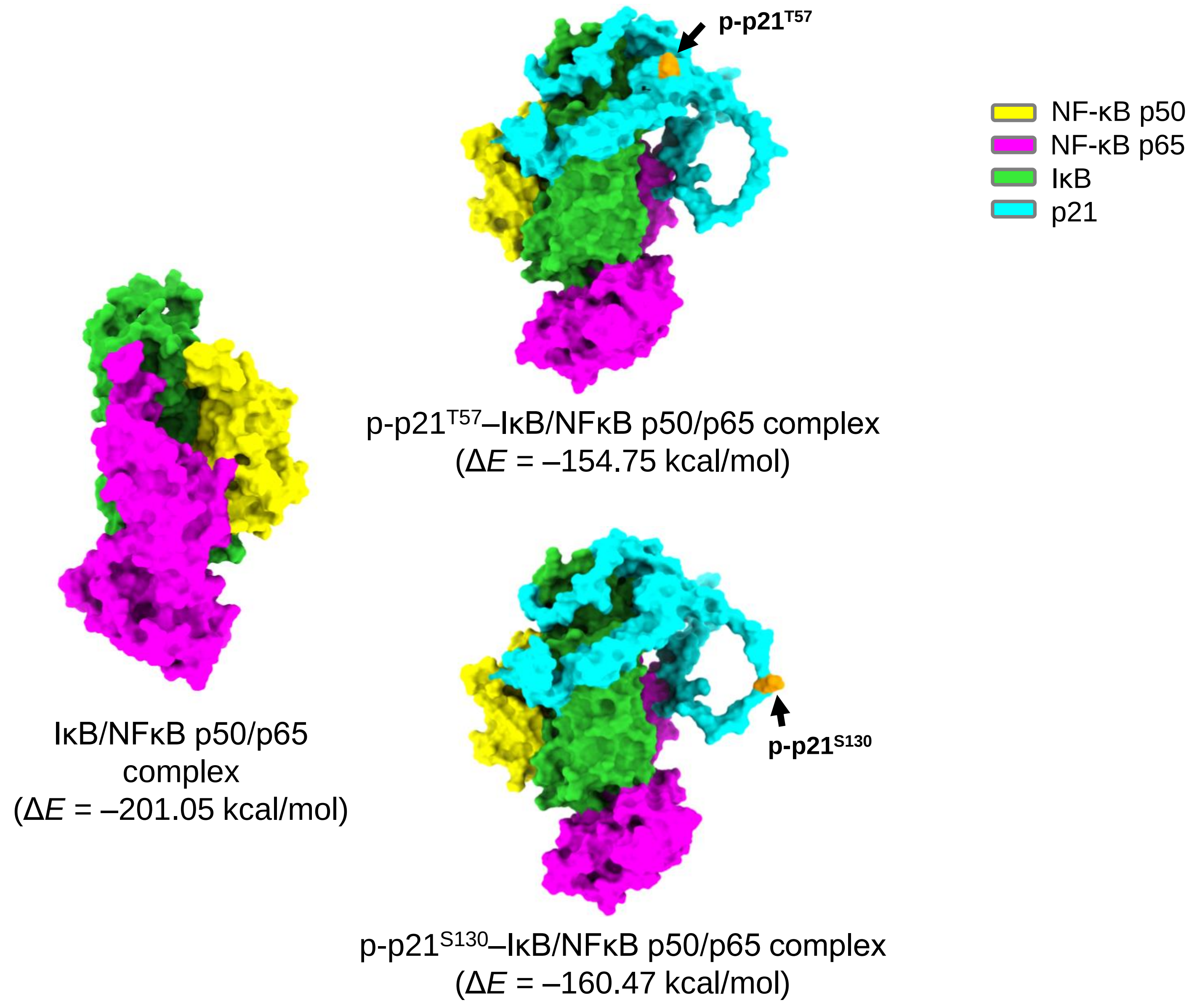
p-p21T57
NF-κB p50
NF-κB p65
IκB
p21
p-p21T57–IκB/NFκB p50/p65 complex
(ΔE = –154.75 kcal/mol)
IκB/NFκB p50/p65
complex
(ΔE = –201.05 kcal/mol)
p-p21S130
p-p21S130–IκB/NFκB p50/p65 complex
(ΔE = –160.47 kcal/mol)

Figure S8
